# Oncologic and Reproductive Outcomes of Fertility-Sparing Management in Early-Stage Endometrial Carcinoma: A Systematic Review and Meta-Analysis

**DOI:** 10.3390/cancers18030399

**Published:** 2026-01-27

**Authors:** Pál Sebok, Márton Keszthelyi, Balázs Vida, Lotti Lőczi, Barbara Sebők, Petra Merkely, Nándor Ács, Ferenc Bánhidy, Attila Keszthelyi, Szabolcs Várbíró, Balázs Lintner, Richárd Tóth

**Affiliations:** 1 Department of Obstetrics and Gynecology, Semmelweis University, 1082 Budapest, Hungary; sebok.pal.szabolcs@semmelweis.hu (P.S.); vida.balazs.lajos@semmelweis.hu (B.V.); keszthelyi.lotti.lucia@semmelweis.hu (L.L.); merkely.petra@gmail.com (P.M.); acs.nandor@semmelweis.hu (N.Á.); banhidy.ferenc@semmelweis.hu (F.B.); lintner.balazs.zoltan@semmelweis.hu (B.L.); toth.richard@semmelweis.hu (R.T.); 2 Workgroup of Research Management, Doctoral School, Semmelweis University, 1085 Budapest, Hungary; sebok.barbara@semmelweis.hu (B.S.); varbiro.szabolcs@semmelweis.hu (S.V.); 3 Department of Urology, Semmelweis University, 1082 Budapest, Hungary; keszthelyi.attila@semmelweis.hu; 4 Department of Obstetrics and Gynecology, University of Szeged, 6725 Szeged, Hungary

**Keywords:** endometrial carcinoma, fertility-sparing, progestin, hysteroscopic resection, levonorgestrel-releasing intrauterine device, premenopausal, GnRHa

## Abstract

**Background/Objectives**: Fertility-sparing treatment is an accepted option for selected women with early-stage endometrial carcinoma, yet comparative evidence on oncologic durability and reproductive success remains limited. This systematic review and meta-analysis aimed to evaluate oncologic and reproductive outcomes across available fertility-preserving strategies in early-stage endometrial carcinoma. **Methods**: A systematic search of MEDLINE, Embase, Web of Science, CENTRAL, and Scopus was conducted from inception to 13 April 2025. Eligible studies included women of reproductive age with FIGO stage IA endometrial carcinoma undergoing fertility-sparing treatment. Studies enrolling mixed populations were included only if carcinoma-specific outcomes could be clearly identified; cases of atypical endometrial hyperplasia or endometrial intraepithelial neoplasia were not included in the quantitative synthesis. Outcomes included the rates of complete response, recurrence, partial response, no response, pregnancy, and live birth. Meta-analyses with random-effect models were performed, with subgroup analyses by treatment strategy. Risk of bias was assessed using ROBINS-I and RoB 2 tools. The review was preregistered in PROSPERO. Meta-analyses were conducted using R software, v4.4.2 (R Foundation for Statistical Computing, Vienna, Austria). **Results**: Seventy-six studies involving 2507 women were included. The pooled complete response rate was 74% (95% CI: 69–79%), while recurrence occurred in 35% (95% CI: 28–42%) of complete responders. Strategies incorporating hysteroscopic resection, alongside other combination regimens, consistently demonstrated higher complete response rates (85%) and lower recurrence (14–16%) compared with oral progestin or LNG-IUD monotherapy. Early treatment failure, with pooled partial and no response rates of 6% and 16%, respectively, particularly affecting monotherapy regimens. The pooled pregnancy and live birth rates were 48% (95% CI: 41–54%) and 36% (95% CI: 29–43%), with more consistent reproductive outcomes observed with combination strategies. **Conclusions**: Fertility-sparing treatment in early-stage endometrial carcinoma is associated with high initial remission rates; however, the durability of response remains limited, while overall reproductive success is moderate. Accumulating evidence suggests that combination strategies, particularly those incorporating hysteroscopic resection, are associated with more favorable oncologic control and reproductive outcomes compared with hormonal monotherapy. Accordingly, fertility preservation should be regarded as a carefully selected, time-limited approach that requires close surveillance and individualized patient counseling.

## Simple Summary

Early-stage endometrial cancer is increasingly diagnosed in young women who wish to preserve their fertility, even though standard treatment involves removal of the uterus. Conservative, fertility-sparing treatments using hormonal therapy or minimally invasive surgery have therefore been developed, but their long-term effectiveness and reproductive success remain uncertain. We reviewed and analyzed data from 76 studies including more than 2500 women with early-stage endometrial cancer treated conservatively. Overall, approximately 70–75% of women achieved an initial remission with fertility-sparing treatment, although cancer recurrence occurred in about 30–35% of those who responded. Strategies combining hormonal therapy with hysteroscopic removal of visible disease were associated with higher remission durability and lower rates of early treatment failure compared with hormonal treatment alone. Following remission, conception occurred in approximately 45–50% of women attempting pregnancy, while 35–40% resulted in live births. Together, these findings illustrate both the potential benefits and the inherent limitations of fertility-sparing treatment and may help support realistic counseling and individualized clinical decision-making.

## 1. Introduction

Endometrial carcinoma (EC) is the leading gynecologic malignancy in high-income countries, with a consistent upward trend in incidence observed among younger women, particularly in those younger than 50 years [1]. An estimated 5–7% of endometrial carcinoma diagnoses occur in women under 45 years of age, reflecting the rising burden of obesity and polycystic ovary syndrome (PCOS) in this population [2]. In disease stages I–II, standard management consists of total hysterectomy with bilateral salpingectomy or salpingo-oophorectomy, depending on patient age and disease stage; however, this approach poses a major clinical dilemma, as it results in the irreversible loss of reproductive potential [3].

Atypical endometrial hyperplasia (AEH) and endometrial intraepithelial neoplasia (EIN) represent premalignant endometrial lesions characterized by cytologic atypia and architectural glandular abnormalities, with an increased risk of progression to carcinoma. In contrast, early-stage invasive endometrial cancer refers to histologically confirmed endometrioid carcinoma confined to the endometrium, corresponding to FIGO stage IA disease without myometrial invasion. In the present review, the term “early-stage endometrial carcinoma” is used to denote early-stage invasive endometrial cancer and does not include premalignant conditions.

Over the past two decades, fertility-preserving strategies have been developed to address this dilemma, aiming to preserve uterine function while maintaining oncologic safety [4]. Current international guidelines, including those of ESGO/ESTRO/ESP, NCCN, and ESMO, support fertility-sparing treatment in carefully selected patients with early-stage, low-grade endometrial carcinoma who wish to preserve fertility [5,6]. These approaches most commonly involve oral progestins or levonorgestrel-releasing intrauterine devices (LNG-IUDs), often preceded by hysteroscopic tumor resection to optimize disease control and reduce relapse risk.

Progestin-based therapy acts through progesterone receptor activation in endometrial cells, inducing antiproliferative effects, decidualization, glandular atrophy, and apoptosis, while also suppressing estrogen receptor signaling and downstream pathways such as p21 and Bcl-2 [7]. LNG-IUDs achieve high local hormone exposure with minimal systemic effects, but share similar cellular mechanisms with oral progestins [8]. Emerging evidence suggests that combination regimens may further enhance therapeutic efficacy. Therapy including metformin increases progesterone receptor expression and inhibits PI3K/AKT/mTOR–mediated proliferation, while gonadotropin-releasing hormone (GnRH) analogs reduce estrogen production and directly promote cell-cycle arrest and apoptosis via GnRH receptors [9]. Hysteroscopic resection complements medical therapy by mechanically removing visible lesions, reducing tumor burden, and improving responsiveness to subsequent hormonal treatment.

In patients with early-stage endometrial carcinoma, reported complete response rates reach 70–80% within 6–12 months of conservative treatment; however, disease recurrence occurs in approximately 30% of cases [10]. Although adjunctive agents such as metformin and GnRH analogs have been explored [11], most recommendations still rely on monotherapy or limited pharmacological combinations, and robust comparative evidence remains scarce. Key challenges include defining the optimal duration of therapy and improving the durability of oncologic response while preserving reproductive outcomes.

Importantly, this review focuses exclusively on endometrial carcinoma and does not include outcomes related to atypical endometrial hyperplasia or endometrial intraepithelial neoplasia. This review therefore aims to provide a comprehensive, EC-specific evaluation of fertility-preserving treatments, focusing exclusively on oncologic and reproductive outcomes in endometrial carcinoma. By synthesizing data on complete response, recurrence, pregnancy, and live birth rates across hormonal, surgical, and combined approaches, we seek to address critical evidence gaps and inform future guideline development tailored specifically to young women with EC.

## 2. Objectives

The objective of this review is to identify the most effective fertility-preserving treatment strategy for women with early-stage endometrial cancer, defined as the approach that achieves an optimal balance between oncologic safety and reproductive outcomes, with the highest rates of complete response, pregnancy, and live birth, and the lowest recurrence rate.

## 3. Materials and Methods

### 3.1. Study Selection Criteria

The population–intervention–control–outcome (PICO) framework applied in this study was as follows:

- Population: Women of reproductive age diagnosed with FIGO stage IA endometrial carcinoma who received conservative, fertility-preserving management.
- Intervention: Fertility-sparing interventions included systemic and intrauterine progestins, as well as multimodal regimens such as the addition of metformin or gonadotropin-releasing hormone (GnRH) analogs, and hysteroscopic tumor resection. In the available literature, hysteroscopic resection was combined with either oral or intrauterine progestins, while no study reported its use together with both modalities concurrently.
- Control: Other fertility-sparing treatment strategies drawn from the same predefined intervention set, enabling pairwise and subgroup comparisons across the full spectrum of conservative treatment modalities.
- Outcomes: Oncologic outcomes included complete response (CR) rate and recurrence rate. Reproductive outcomes encompassed pregnancy and live birth rates (LBR). Secondary endpoints were partial response (PR) rate, no response (NR) rate.

All included studies primarily enrolled women with histologically confirmed endometrioid endometrial carcinoma confined to the uterus, corresponding to FIGO stage IA, grade 1 disease, which represents the accepted indication for fertility-sparing management. Studies that included mixed populations, such as atypical endometrial hyperplasia alongside carcinoma, were eligible only if outcome data for carcinoma patients could be clearly identified or if carcinoma-specific outcomes were reported separately. A small number of studies reported the inclusion of isolated grade 2 endometrioid tumors; however, these cases represented a very limited minority of the overall population, were not the focus of treatment protocols, and could not be analyzed separately due to aggregated reporting. As such, they are unlikely to have materially influenced the meta-analytic estimates. Importantly, no included study involved patients with myometrial invasion beyond stage IA or non-endometrioid histology.

### 3.2. Search Sources and Strategy

A comprehensive literature search was conducted in Embase, Web of Science, CENTRAL, MEDLINE (via PubMed), and Scopus up to 13 April 2025, without restrictions on the start date. The search strategy combined Medical Subject Headings (MeSH) with relevant free-text keywords to ensure comprehensive identification of eligible studies. The specific search terms used are detailed below. The search strategy included the following terms: (“endometrium” OR “endometrial” OR “endometrioid” OR “endometr*”) AND (“carcinoma” OR “carcino*” OR “cancer*”) AND (“early stage” OR “stage I” OR “FIGO I” OR “low-risk” OR “fertility-sparing” OR “fertility preserv*” OR “conservative management”) AND (“gestagen” OR “gest*” OR “progesterone” OR “progest*” OR “medroxyprogesterone acetate” OR “medroxyprogesterone” OR “progesterone derivative” OR “megestrol acetate” OR “dienogest derivative” OR “levonorgestrel” OR “hydroxyprogesterone” OR “medrogestone” OR “megestrol” OR “desogestrel derivative” OR “drospirenone” OR “dydrogesterone” OR “intrauterine device” OR “IUD” OR “metformin” OR “GnRHa” OR “gonadotropin-releasing hormone agonist” OR “GnRH analogue” OR “hysteroscopy” OR “hysteros*” OR “hysteroscopic resect*”).

This review was developed in accordance with the PRISMA framework [12] (Supplementary Tables S2 and S3), with methodological guidance drawn from the Cochrane Handbook for Systematic Reviews of Interventions [13], and was formally submitted to PROSPERO on 13 April 2025 (CRD420251032161) [14].

### 3.3. Study Selection Process

All retrieved citations were imported into EndNote v21 (Clarivate Analytics, Philadelphia, PA, USA, 2025) for reference management and duplicate removal. Following de-duplication, records were uploaded to Rayyan (v1.5.3; Rayyan Systems Inc., Cambridge, MA, USA). Two reviewers (P.S.; B.V.) independently screened titles and abstracts according to the predefined eligibility criteria, after which, full texts of potentially relevant studies were assessed. Any disagreements were resolved through discussion or, when necessary, by consultation with a third reviewer (M.K.). Data extraction was performed independently by two reviewers (P.S.; B.V.) using a standardized Excel spreadsheet (Microsoft Excel, Office 365; Microsoft Corporation, Redmond, WA, USA, 1985), with discrepancies resolved by consensus or arbitration by a third reviewer (M.K.). (Supplementary Table S4).

### 3.4. Variables and Outcome Measures

A predefined data extraction form was used to ensure consistency across studies. Extracted study-level variables included first author, year of publication, study design (e.g., randomized controlled trial or cohort study), and sample size. Intervention-related data encompassed the type of fertility-sparing therapy, comparators when applicable, and duration of follow-up. Baseline patient characteristics, including mean or median age and body mass index (BMI), were also collected.

Outcomes of interest included the complete response rate, defined as the absence of endometrial carcinoma or atypical hyperplasia on follow-up endometrial assessment; the partial response rate, defined as regression of carcinoma with persistence of endometrial hyperplasia with or without atypia; the recurrence rate, defined as histologically confirmed disease reappearance after an initial CR; and the no response rate, indicating persistence or progression of disease despite conservative treatment. Follow-up evaluation was most commonly performed by dilatation and curettage, while endometrial sampling or hysteroscopic-guided sampling or resection was also used in some studies, depending on institutional practice. In several reports, the specific follow-up sampling method was not distinguished at the individual patient level; however, histologic confirmation of remission was required in all studies declaring CR. Additional outcomes included time to response, defined as the interval from treatment initiation to documented CR, as well as reproductive endpoints. Pregnancy and live birth rates were extracted preferentially among women who achieved complete response and were explicitly reported to have actively attempted conception. In retrospective studies, verification relied on study-level reporting of reproductive intent, fertility attempts, or follow-up restricted to women pursuing pregnancy. When the number of women attempting conception was not reported, reproductive outcomes were calculated using the number of complete responders as the denominator. In cases of missing, incomplete, or unclear information, corresponding authors were contacted for clarification.

### 3.5. Assessment of Methodological Quality and Bias

Risk of bias was assessed independently by two reviewers (M.K. and P.M.). For non-randomized studies, the ROBINS-I tool was applied, covering confounding, selection, intervention classification, deviations from intended interventions, outcome data completeness, outcome measurement, and selective reporting. For randomized controlled trials, the Cochrane Risk of Bias 2 (RoB 2) tool was used to evaluate randomization, adherence to interventions, integrity of outcome data, measurement, and reporting bias. Disagreements were resolved by discussion or adjudication by a third reviewer (R.T.).

Potential meta-biases were also examined. Publication bias and selective reporting were assessed through visual inspection of funnel plots when at least ten studies reported an outcome. Funnel plot asymmetry was further tested using Egger’s and Begg’s tests (*p* < 0.10). Reported outcomes were compared with trial registries or published protocols, where available to detect selective non-reporting. Subgroup and sensitivity analyses were conducted to examine the robustness of findings in the presence of possible bias.

### 3.6. Statistical Analysis and Evidence Synthesis

In accordance with the preregistered protocol, all quantitative syntheses were conducted using a random-effects meta-analytic framework. Meta-analyses were performed when at least three studies reported sufficiently comparable outcome data. For dichotomous outcomes, risk ratios (RRs) with 95% confidence intervals (CIs) were calculated using the Mantel–Haenszel method, applying the exact variant in the presence of zero-event cells. In parallel, event proportions were pooled separately within each treatment group. For continuous outcomes, mean differences (MDs) or differences between medians (MedDs) were reported depending on data availability. When studies reported only quartiles, means and standard deviations (SDs) were estimated assuming normal or lognormal distributions; when such assumptions were not justifiable, medians were synthesized directly. When only aggregate data were available, pooled RRs and MDs were estimated using the inverse variance method.

To enhance the robustness of statistical inference, confidence intervals were adjusted using the Hartung–Knapp method when this approach yielded more conservative estimates. Between-study heterogeneity was assessed using Higgins’ I^2^ statistic, with τ^2^ estimated by restricted maximum likelihood (REML) and corresponding confidence intervals derived using the Q-profile method. Prediction intervals were reported when at least three studies contributed to an analysis. Statistical significance was defined by confidence intervals that did not cross the null value. Results were presented in forest plots, and model diagnostics included both visual inspection and formal influence analyses to identify potential outlying studies.

For trials with multiple intervention arms, three-level multivariate random-effects models were applied to account for correlated outcomes without imposing arbitrary correlation coefficients. Conventional two-level random-effects models were also fitted to evaluate the robustness of the findings. Sensitivity analyses were conducted by sequentially excluding studies with very large sample sizes or extreme effect estimates. Subgroup analyses were performed when at least three studies contributed to a given intervention–outcome comparison. Patient age and body mass index were consistently reported across studies, primarily as aggregated summary measures. Although these variables were reviewed descriptively, no formal stratified analyses according to age or body mass index were performed, as their between-study distributions largely overlapped and did not permit meaningful separation into clinically distinct subgroups. In contrast, predefined subgroup analyses were conducted according to treatment strategy, with interventions analyzed separately for each outcome when supported by at least three studies.

Potential small-study effects and publication bias were evaluated using funnel plots, supplemented by Peters’ regression or Begg’s test when at least ten studies were available. For time-to-event outcomes, such as time to complete response or time to recurrence, reported medians and ranges or interquartile ranges were reviewed to assess the feasibility of time-to-event synthesis. However, due to substantial heterogeneity in reporting and follow-up metrics, these outcomes were not subjected to formal pooled time-to-event analyses.

Pooled meta-analyses were conducted irrespective of study design when data were available; however, due to outcome-specific data availability, randomized controlled trials contributed only to the analyses of complete response, pregnancy, and live birth outcomes, while recurrence, partial response, and no response analyses were based exclusively on cohort studies.

All statistical analyses were conducted in R (v4.4.2; R Core Team, Vienna, Austria), using the meta package (v7.0.0) for primary analyses and forest plots, dmetar (v0.1.0) for influence diagnostics, metafor (v4.6.0) for multivariate modeling, and ggplot2 (v3.5.1) for data visualization.

### 3.7. Evaluation of the Strength of Evidence

The certainty of evidence for each outcome is assessed using the GRADE approach, which considers risk of bias, inconsistency, indirectness, imprecision, and publication bias. Outcomes are rated as high, moderate, low, or very low certainty. Evidence from randomized trials will start as high, but may be downgraded if concerns are identified, while evidence from non-randomized studies will begin as low, and may be upgraded in cases of large effects, dose–response relationships, or when confounding would likely reduce the observed effect. Findings and certainty ratings will be presented in Summary of Findings tables generated with GRADEpro GDT, with all rating decisions clearly justified [15] (Supplementary Table S1).

## 4. Results

### 4.1. Study Identification and Selection

**Figure 1:**
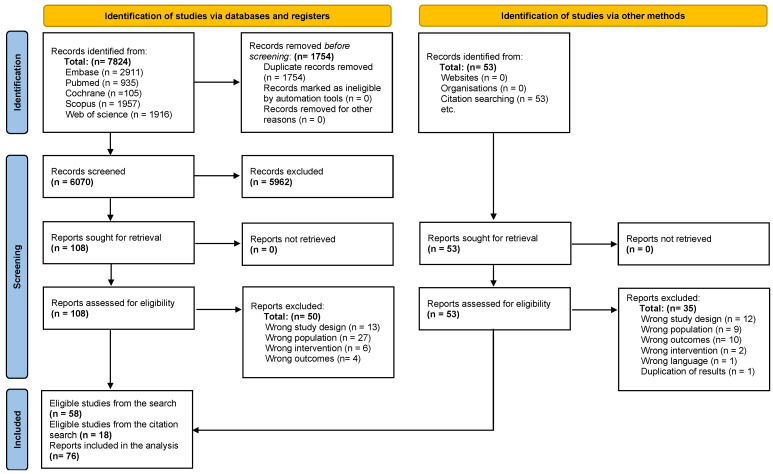
PRISMA 2020 flow diagram of study selection.

The systematic search across electronic databases retrieved 7824 records. After removal of duplicate entries, 6070 unique publications were subjected to title and abstract screening. Of these, 108 articles were reviewed in full text for eligibility, and 49 were excluded at the data extraction stage due to failure to meet inclusion criteria. The final dataset comprised 56 cohort studies and 2 randomized controlled trials [16,17]. Manual screening of reference lists yielded an additional 18 eligible cohort studies. In total, 76 studies (28 prospective [18,19,20,21,22,23,24,25,26,27,28,29,30,31,32,33,34,35,36,37,38,39,40,41,42,43,44,45] and 46 retrospective [46,47,48,49,50,51,52,53,54,55,56,57,58,59,60,61,62,63,64,65,66,67,68,69,70,71,72,73,74,75,76,77,78,79,80,81,82,83,84,85,86,87,88,89,90,91] cohort studies, 2 randomized controlled trials [16,17]) [16,17,18,19,20,21,22,23,24,25,26,27,28,29,30,31,32,33,34,35,36,37,38,39,40,41,42,43,44,45,46,47,48,49,50,51,52,53,54,55,56,57,58,59,60,61,62,63,64,65,66,67,68,69,70,71,72,73,74,75,76,77,78,79,80,81,82,83,84,85,86,87,88,89,90,91] involving 2507 patients were included in the qualitative and quantitative synthesis (Figure 1). Detailed clinical characteristics of the included studies and study populations are provided in Table 1 and Table 2.

### 4.2. Assessment of the Risk of Bias

Because the evidence base was largely composed of non-randomized studies, risk of bias was primarily assessed using the ROBINS-I framework, which was applied to 74 observational investigations. The two randomized controlled trials were evaluated separately using the Cochrane Risk of Bias 2 (RoB 2) tool. Overall, the majority of observational studies were judged to be at moderate risk of bias, reflecting the inherent methodological challenges of retrospective fertility-sparing research; a subset demonstrated comparatively strong methodological rigor and a low likelihood of bias, while a limited number were assessed as having more substantial risk in specific domains. Both randomized trials were prospectively registered in publicly accessible clinical trial registries prior to participant enrolment. Risk of bias assessments were conducted according to pre-specified criteria and are presented graphically using the robvis visualization platform, with detailed judgments reported in Supplementary Figure S1.

### 4.3. Synthesis of Results

#### 4.3.1. Complete Response Rates

**Figure 2:**
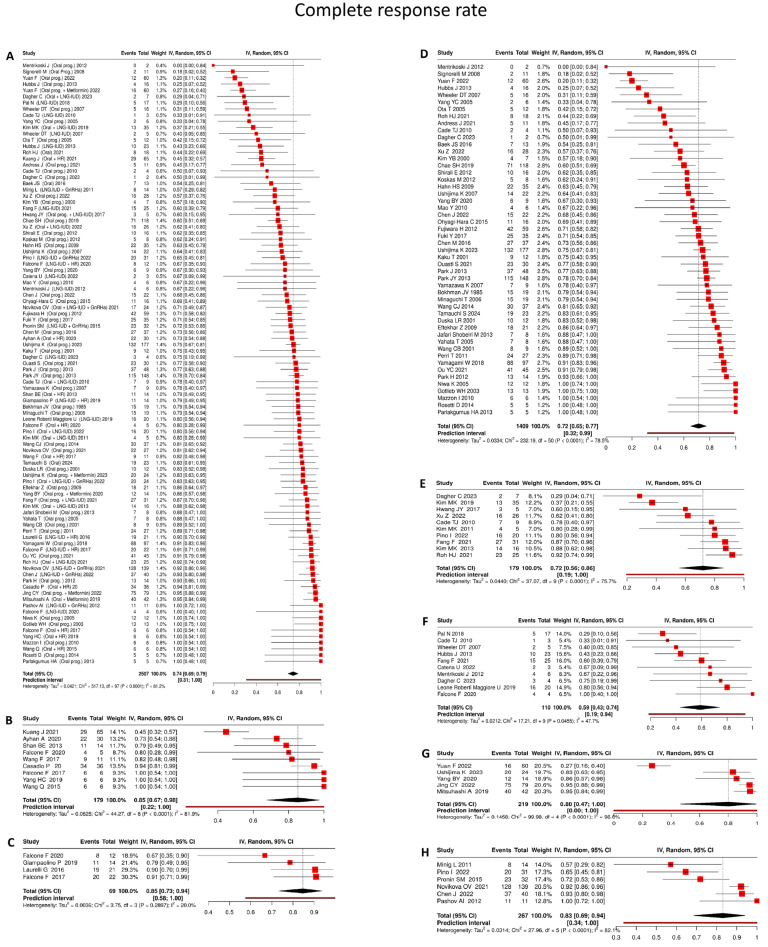
(**A**) Overall, (**B**) Oral progestin plus hysteroscopic resection, (**C**) LNG-IUD plus hysteroscopic resection, (**D**) Oral progestin, (**E**) Oral progestin plus LNG-IUD, (**F**) LNG-IUD, (**G**) Oral progestin plus metformin, (**H**) LNG-IUD plus GnRHa. Forest plots showing complete response rates in women with early stage endometrial cancer treated with fertility-sparing interventions. Pooled analyses are based on observational cohort studies and randomized controlled trials. Red squares: Study-specific complete response rate; square size represents study weight. Horizontal lines: 95% confidence intervals for each study. Black diamond: Pooled effect estimate with its 95% confidence interval. Vertical dotted line: Overall pooled effect estimate (reference line). Heterogeneity statistics: I^2^, τ^2^, and *p*-value describe between-study variability [16,17,18,19,20,21,22,23,24,25,26,27,28,29,30,31,32,33,34,35,36,37,38,39,40,41,42,43,44,45,46,47,48,49,50,51,52,53,54,55,56,57,58,59,60,61,62,63,64,65,66,67,68,69,70,71,72,73,74,75,76,77,78,79,80,81,82,83,84,85,86,87,88,89,90,91].

Across all included studies [16,17,18,19,20,21,22,23,24,25,26,27,28,29,30,31,32,33,34,35,36,37,38,39,40,41,42,43,44,45,46,47,48,49,50,51,52,53,54,55,56,57,58,59,60,61,62,63,64,65,66,67,68,69,70,71,72,73,74,75,76,77,78,79,80,81,82,83,84,85,86,87,88,89,90,91] evaluating fertility-sparing treatments for early-stage endometrial carcinoma, the pooled complete response (CR) rate was 74% (95% CI: 69–79%), based on 2507 patients, with substantial heterogeneity (I^2^ = 81.2%) (Figure 2A). This finding indicates that conservative management is generally effective in achieving histologic remission, although response rates vary considerably across treatment strategies and clinical settings.

Oral progestin

Based on 50 studies [16,17,22,26,29,30,31,35,36,38,39,40,42,43,44,47,48,49,54,55,57,59,61,65,67,68,70,71,73,74,75,76,78,79,80,82,83,85,86,87,88,89,91] encompassing 1409 patients, oral progestin monotherapy was associated with a pooled complete response rate of 72% (95% CI: 65–77%), accompanied by considerable heterogeneity (I^2^ = 78.5%) (Figure 2D).

LNG-IUD

In analyses of 10 studies [19,53,56,71,72,76,79,83,89,90] including 110 patients, LNG-IUD monotherapy achieved a pooled complete response rate of 59% (95% CI: 43–74%), with moderate between-study heterogeneity (I^2^ = 47.7%) (Figure 2F).

Oral progestin + LNG-IUD

Among 10 studies [17,19,23,28,45,55,71,77,84,89] comprising 179 patients, combined treatment with oral progestins and LNG-IUD produced a pooled complete response rate of 72% (95% CI: 56–86%), although substantial heterogeneity was observed (I^2^ = 75.7%) (Figure 2E).

Oral progestin + hysteroscopic resection

Across 9 studies [18,27,37,41,46,50,56,58,60] involving 179 patients, hysteroscopic tumor resection followed by oral progestin therapy resulted in a pooled complete response rate of 85% (95% CI: 67–98%), despite marked heterogeneity (I^2^ = 81.9%) (Figure 2B).

LNG-IUD + hysteroscopic resection

In 4 studies [24,27,51,56] including 69 patients, LNG-IUD insertion after hysteroscopic resection yielded a pooled complete response rate of 85% (95% CI: 73–94%), with low heterogeneity across studies (I^2^ = 20%) (Figure 2C).

LNG-IUD + GnRHa

Data from 6 studies [20,21,29,32,33,84] comprising 267 patients indicated that combined LNG-IUD and GnRHa therapy was associated with a pooled complete response rate of 83% (95% CI: 69–94%), in the context of very high heterogeneity (I^2^ = 82.1%) (Figure 2H).

Oral progestin + metformin

Across 5 studies [16,36,64,69,82] including 219 patients, the addition of metformin to oral progestin therapy resulted in a pooled complete response rate of 80% (95% CI: 47–100%); however, this estimate was characterized by extreme heterogeneity (I^2^ = 96%) (Figure 2G).

#### 4.3.2. Recurrence Rate

**Figure 3:**
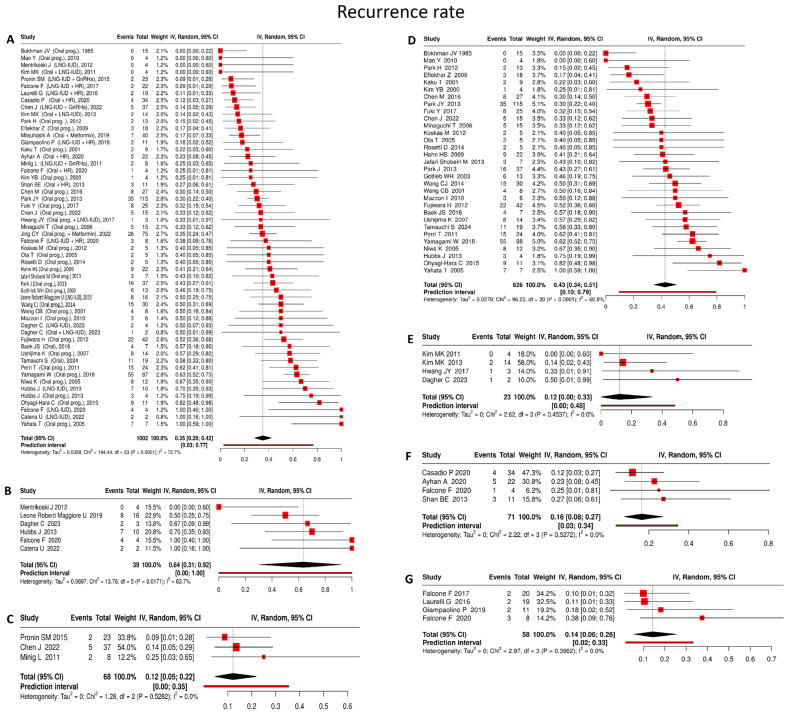
(**A**) Overall, (**B**) LNG-IUD, (**C**) LNG-IUD plus GnRHa, (**D**) Oral progestin, (**E**) Oral progestin plus LNG-IUD, (**F**) Oral progestin plus hysteroscopic resection, (**G**) LNG-IUD plus hysteroscopic resection. Forest plots showing recurrence rates in women with early stage endometrial cancer treated with fertility-sparing interventions. Pooled analyses are based exclusively on cohort studies. Red squares: Study-specific complete response rate; square size represents study weight. Horizontal lines: 95% confidence intervals for each study. Black diamond: Pooled effect estimate with its 95% confidence interval. Vertical dotted line: Overall pooled effect estimate (reference line). Heterogeneity statistics: I^2^, τ^2^, and *p*-value describe between-study variability [18,20,22,24,25,27,28,29,31,32,34,35,39,40,42,45,46,47,49,50,51,52,53,54,56,57,61,62,63,66,67,68,70,72,74,75,76,77,78,79,80,81,85,86,88,89,91].

Among 1002 patients with available follow-up data who achieved an initial complete response, the pooled recurrence rate was 35% (95% CI: 28–42%), with high heterogeneity (I^2^ = 72.7%) (Figure 3A):

Oral progestin

Drawing on 31 studies [22,25,29,31,34,35,39,40,42,47,49,52,54,57,61,62,63,66,67,68,70,74,75,76,78,80,81,85,86,88,91] comprising 626 patients, oral progestin therapy was associated with a pooled recurrence rate of 43% (95% CI: 34–51%), with substantial between-study heterogeneity (I^2^ = 68.8%) (Figure 3D).

LNG-IUD

In 6 studies [53,56,72,76,79,89] including 39 patients, LNG-IUD monotherapy showed a pooled recurrence rate of 64% (95% CI: 31–92%), accompanied by considerable heterogeneity (I^2^ = 63.7%) (Figure 3B).

Oral progestin + LNG-IUD

Across 4 studies [28,45,77,89] involving 23 patients, the combination of oral progestins and LNG-IUD was associated with a pooled recurrence rate of 12% (95% CI: 0–33%), with no evidence of heterogeneity (I^2^ = 0%) (Figure 3E).

Oral progestin + hysteroscopic resection

Based on 4 studies [18,46,50,56] comprising 71 patients, oral progestin therapy administered after hysteroscopic resection yielded a pooled recurrence rate of 16% (95% CI: 8–27%), with no observed heterogeneity (I^2^ = 0%) (Figure 3F).

LNG-IUD + hysteroscopic resection:

In 4 studies including [24,27,51,56] 58 patients, this treatment approach resulted in a pooled recurrence rate of 14% (95% CI: 6–26%), again with no detectable heterogeneity across studies (I^2^ = 0%) (Figure 3G).

LNG-IUD + GnRHa

Across 3 studies [20,29,32] encompassing 68 patients, combined LNG-IUD and GnRHa therapy was associated with a pooled recurrence rate of 12% (95% CI: 5–22%), with no statistical heterogeneity observed (I^2^ = 0%) (Figure 3C).

#### 4.3.3. Pregnancy Rate

**Figure 4:**
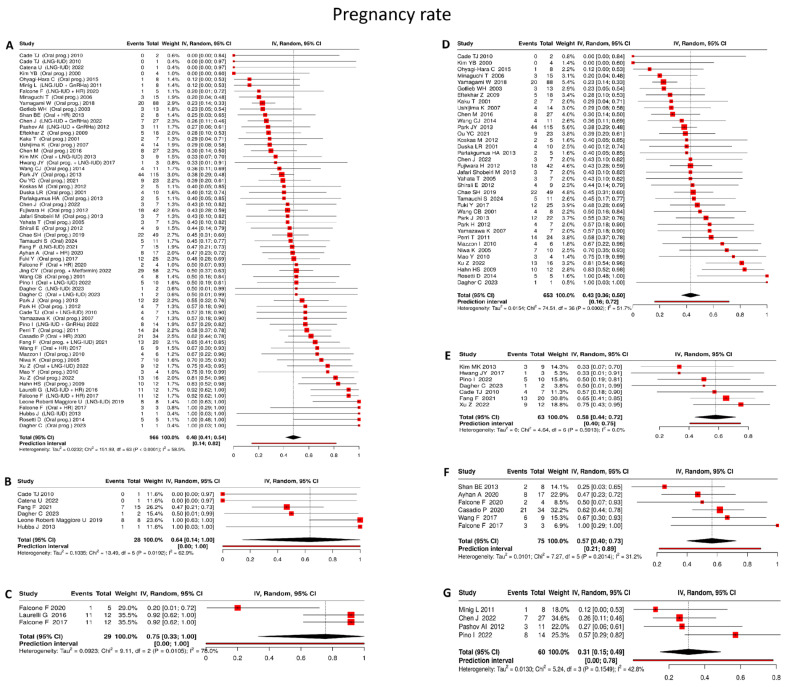
(**A**) Overall, (**B**) LNG-IUD, (**C**) LNG-IUD plus hysteroscopic resection, (**D**) Oral progestin, (**E**) Oral progestin plus LNG-IUD, (**F**) Oral progestin plus hysteroscopic resection, (**G**) LNG-IUD plus GnRHa. Forest plots showing pregnancy rates in women with early stage endometrial cancer treated with fertility-sparing interventions. Pooled analyses are based on observational cohort studies and a randomized controlled trial. Red squares: Study-specific complete response rate; square size represents study weight. Horizontal lines: 95% confidence intervals for each study. Black diamond: Pooled effect estimate with its 95% confidence interval. Vertical dotted line: Overall pooled effect estimate (reference line). Heterogeneity statistics: I^2^, τ^2^, and *p*-value describe between-study variability [17,18,19,20,21,22,23,24,25,26,27,28,29,31,35,39,40,42,43,46,47,49,50,52,53,56,57,59,60,61,62,63,65,66,67,68,70,71,72,73,74,75,76,77,78,80,84,85,86,87,88,89,91].

Across all studies reporting reproductive outcomes following fertility-sparing management of early-stage endometrial carcinoma, the pooled pregnancy rate was 48% (95% CI: 41–54%), based on 966 women, with moderate heterogeneity (I^2^ = 58.5%) (Figure 4A).

Oral progestin

Based on 37 studies [17,22,25,26,29,31,35,39,40,42,43,47,49,52,57,59,61,62,63,65,66,67,68,70,71,73,74,75,78,80,85,86,87,88,89,91] encompassing 653 women, oral progestin monotherapy was associated with a pooled pregnancy rate of 43% (95% CI: 36–50%), with moderate between-study heterogeneity (I^2^ = 51.7%) (Figure 4D).

LNG-IUD

In analyses of 6 studies [19,53,71,72,76,89] including 28 patients, LNG-IUD monotherapy resulted in a pooled pregnancy rate of 64% (95% CI: 14–100%), accompanied by substantial heterogeneity (I^2^ = 62.9%) (Figure 4B).

Oral progestin + LNG-IUD

Across 7 studies [17,19,28,71,77,84,89] involving 63 patients, combined treatment with oral progestins and LNG-IUD yielded a pooled pregnancy rate of 58% (95% CI: 44–72%), with no evidence of heterogeneity (I^2^ = 0%) (Figure 4E).

Oral progestin + hysteroscopic resection

Drawing on 6 studies [18,27,46,50,56,60] comprising 75 women, oral progestin therapy following hysteroscopic resection achieved a pooled pregnancy rate of 57% (95% CI: 40–73%), with moderate heterogeneity observed across studies (I^2^ = 31.2%) (Figure 4F).

LNG-IUD + hysteroscopic resection

In 3 studies [24,27,56] including 29 patients, the combination of LNG-IUD placement and hysteroscopic resection was associated with a pooled pregnancy rate of 75% (95% CI: 33–100%), although heterogeneity was high (I^2^ = 78%) (Figure 4C).

LNG-IUD + GnRHa

Across 4 studies [20,21,29,84] encompassing 60 women, combined LNG-IUD and GnRHa therapy demonstrated a pooled pregnancy rate of 31% (95% CI: 15–49%), with moderate heterogeneity (I^2^ = 42.8%) (Figure 4G).

#### 4.3.4. Live Birth Rate

**Figure 5:**
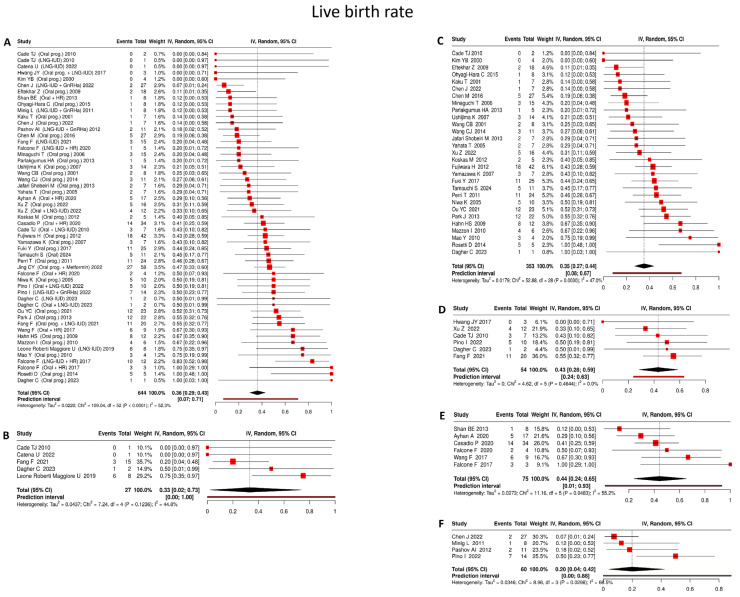
(**A**) Overall, (**B**) LNG-IUD, (**C**) Oral progestin, (**D**) Oral progestin plus LNG-IUD, (**E**) Oral progestin plus hysteroscopic resection, (**F**) LNG-IUD plus GnRHa. Forest plots showing live birth rates in women with early stage endometrial cancer treated with fertility-sparing interventions. Pooled analyses are based on observational cohort studies and a randomized controlled trial. Red squares: Study-specific complete response rate; square size represents study weight. Horizontal lines: 95% confidence intervals for each study. Black diamond: Pooled effect estimate with its 95% confidence interval. Vertical dotted line: Overall pooled effect estimate (reference line). Heterogeneity statistics: I^2^, τ^2^, and *p*-value describe between-study variability [17,18,19,20,21,22,25,27,29,31,35,39,40,42,43,46,47,49,50,52,53,56,60,61,63,65,67,68,70,71,72,74,75,77,78,80,84,87,88,89].

Across studies reporting live birth outcomes following fertility-sparing treatment, the pooled live birth rate was 36% (95% CI: 29–43%), with substantial heterogeneity (I^2^ = 52.3%) (Figure 5A). This demonstrates that although pregnancy is achieved in a considerable proportion of patients, successful progression to live birth occurs in a smaller subset:

Oral progestin

Based on 29 studies [17,22,25,31,35,39,40,42,43,47,49,52,61,63,65,67,68,70,71,74,75,78,80,87,88,89] comprising 272 women, oral progestin therapy was associated with a pooled live birth rate of 35% (95% CI: 27–44%), with moderate between-study heterogeneity (I^2^ = 47.0%) (Figure 5C).

LNG-IUD

In 5 studies [19,53,71,72,89] including 27 patients, LNG-IUD monotherapy resulted in a pooled live birth rate of 33% (95% CI: 2–73%), accompanied by moderate heterogeneity (I^2^ = 44.8%) (Figure 5B).

Oral progestin + LNG-IUD

Across 6 studies [17,19,71,77,84,89] involving 54 women, combined oral progestin and LNG-IUD therapy achieved a pooled live birth rate of 43% (95% CI: 28–59%), with no detectable heterogeneity (I^2^ = 0%) (Figure 5D).

Oral progestin + hysteroscopic resection

Drawing on 6 studies [18,27,46,50,56,60] encompassing 75 patients, the combination of oral progestin therapy and hysteroscopic resection yielded a pooled live birth rate of 44% (95% CI: 24–65%), with moderate heterogeneity observed across studies (I^2^ = 55.2%) (Figure 5E).

LNG-IUD + GnRHa

Across 4 studies [20,21,29,84] including 60 women, combined LNG-IUD and GnRHa treatment was associated with a pooled live birth rate of 20% (95% CI: 4–42%), with moderate heterogeneity (I^2^ = 66.5%) (Figure 5F).

#### 4.3.5. Partial Response Rate

**Figure 6:**
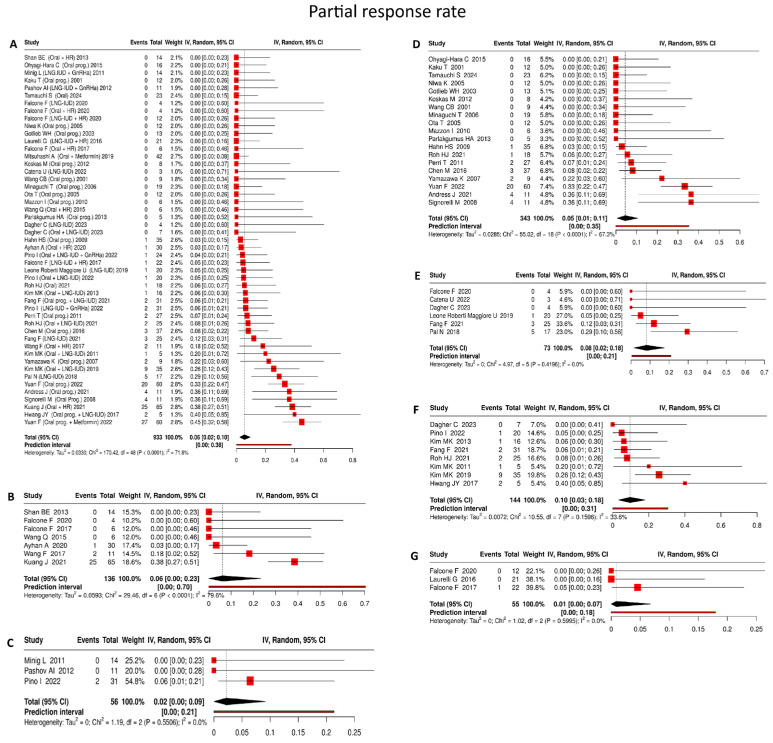
(**A**) Overall, (**B**) Oral progestin plus hysteroscopic, (**C**) LNG-IUD plus GnRHa resection, (**D**) Oral Progestin, (**E**) LNG-IUD, (**F**) Oral progestin plus LNG-IUD, (**G**) LNG-IUD plus hysteroscopic resection. Forest plots showing partial response rates in women with early stage endometrial cancer treated with fertility-sparing interventions. Pooled analyses are based exclusively on cohort studies. Red squares: Study-specific complete response rate; square size represents study weight. Horizontal lines: 95% confidence intervals for each study. Black diamond: Pooled effect estimate with its 95% confidence interval. Vertical dotted line: Overall pooled effect estimate (reference line). Heterogeneity statistics: I^2^, τ^2^, and *p*-value describe between-study variability [18,19,20,21,22,23,24,27,28,36,39,40,41,43,44,45,47,48,49,52,53,55,56,57,58,60,63,68,70,72,77,80,84,85,87,89,90].

Based on 933 patients, the pooled partial response rate was 6% (95% CI: 2–10%), with substantial heterogeneity (I^2^ = 71.8%) (Figure 6A).

Oral progestin

Based on 19 studies [22,36,39,40,43,44,47,48,49,52,55,57,63,68,70,80,85,87] comprising 343 patients, oral progestin monotherapy was associated with a pooled partial response rate of 5% (95% CI: 1–11%), with substantial between-study heterogeneity (I^2^ = 67.3%) (Figure 6D).

LNG-IUD

In 6 studies [19,53,56,72,89,90] including 73 patients, LNG-IUD monotherapy yielded a pooled partial response rate of 8% (95% CI: 2–18%), with no detectable heterogeneity across studies (I^2^ = 0%) (Figure 6E):

Oral progestin + LNG-IUD

Across 8 studies [19,23,28,45,55,77,84,89] involving 144 patients, combined oral progestin and LNG-IUD therapy resulted in a pooled partial response rate of 10% (95% CI: 3–18%), accompanied by moderate heterogeneity (I^2^ = 33.6%) (Figure 6F).

Oral progestin + hysteroscopic resection

Drawing on 7 studies [18,27,41,46,56,58,60] encompassing 133 patients, partial response was observed in 6% (95% CI: 0–23%) of cases following hysteroscopic resection combined with oral progestin therapy, with substantial heterogeneity across studies (I^2^ = 79.6%) (Figure 6B).

LNG-IUD + hysteroscopic resection

In 3 studies [24,27,56] including 55 patients, LNG-IUD placement after hysteroscopic resection was associated with a pooled partial response rate of 1% (95% CI: 0–7%), with no evidence of heterogeneity (I^2^ = 0%) (Figure 6G).

LNG-IUD + GnRHa

Across 3 studies [20,21,84] involving 56 patients, combined LNG-IUD and GnRHa therapy demonstrated a pooled partial response rate of 2% (95% CI: 0–9%), again with no observed heterogeneity (I^2^ = 0%) (Figure 6C).

#### 4.3.6. No Response Rate

**Figure 7:**
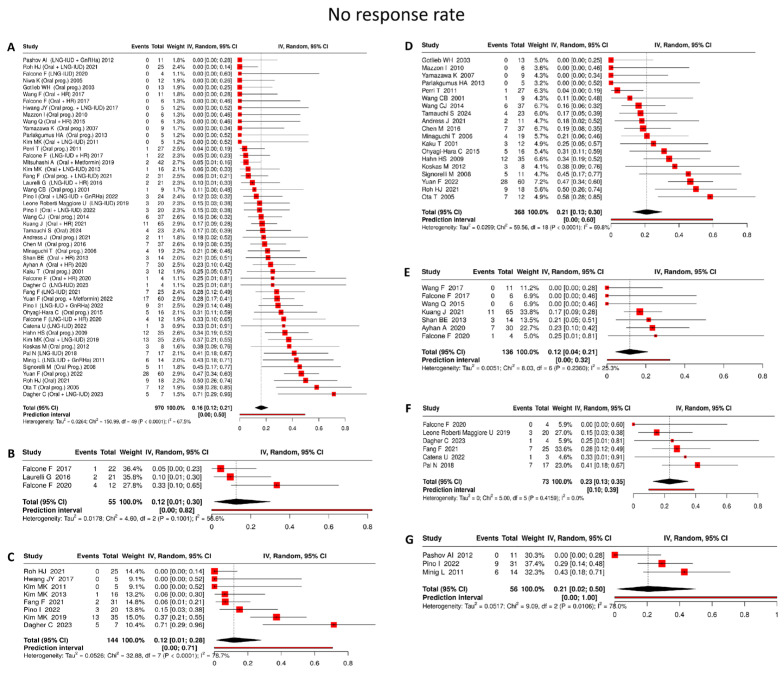
(**A**) Overall, (**B**) LNG-IUD plus hysteroscopic resection, (**C**) Oral progestin plus LNG-IUD, (**D**) Oral Progestin, (**E**) Oral progestin plus hysteroscopic resection, (**F**) LNG-IUD, (**G**) LNG-IUD plus GnRHa. Forest plots showing no response rates in women with early stage endometrial cancer treated with fertility-sparing interventions. Pooled analyses are based exclusively on cohort studies. Red squares: Study-specific complete response rate; square size represents study weight. Horizontal lines: 95% confidence intervals for each study. Black diamond: Pooled effect estimate with its 95% confidence interval. Vertical dotted line: Overall pooled effect estimate (reference line). Heterogeneity statistics: I^2^, τ^2^, and *p*-value describe between-study variability [18,19,20,21,22,23,24,27,28,36,39,40,41,43,44,45,47,48,49,52,53,55,56,57,58,60,63,67,68,70,72,77,80,81,84,85,87,89,90].

Analyzing 970 patients, the pooled no response rate was 16% (95% CI: 12–21%), with moderate heterogeneity (I^2^ = 67.5%) (Figure 7A).

Oral progestin

Based on 20 studies [22,36,39,40,43,44,47,48,49,52,55,57,63,67,68,70,80,81,85,87] comprising 368 patients, oral progestin monotherapy was associated with a pooled no response rate of 21% (95% CI: 13–30%), with substantial between-study heterogeneity (I^2^ = 69.8%) (Figure 7D).

LNG-IUD

In 6 studies [19,53,56,72,89,90] including 73 patients, LNG-IUD monotherapy resulted in a pooled no response rate of 23% (95% CI: 13–35%), with no detectable heterogeneity across studies (I^2^ = 0%) (Figure 7F).

Oral progestin + LNG-IUD

Across 8 studies [19,23,28,45,55,77,84,89] involving 144 patients, combined oral progestin and LNG-IUD therapy yielded a pooled no response rate of 12% (95% CI: 1–28%), accompanied by substantial heterogeneity (I^2^ = 78.7%) (Figure 7C).

Oral progestin + hysteroscopic resection

Drawing on 7 studies [18,27,41,46,56,58,60] encompassing 136 patients, oral progestin therapy following hysteroscopic resection was associated with a pooled no response rate of 12% (95% CI: 4–21%), with low heterogeneity observed across studies (I^2^ = 25.3%) (Figure 7E).

LNG-IUD + hysteroscopic resection

In 3 studies [24,27,56] including 55 patients, LNG-IUD placement after hysteroscopic resection was associated with the lowest pooled no response rate at 12% (95% CI: 1–30%), with no evidence of heterogeneity (I^2^ = 0%) (Figure 7B).

LNG-IUD + GnRHa

Across 3 studies [20,21,84] involving 56 patients, combined LNG-IUD and GnRHa therapy demonstrated a pooled no response rate of 21% (95% CI: 2–50%), with substantial heterogeneity (I^2^ = 78%) (Figure 7G).

#### 4.3.7. Weighted Mean Time to CR and Weighted Mean Time to Recurrence

Across studies reporting time to response, most fertility-sparing treatments achieved histologic remission within a few months, typically between 3 and 10 months. Oral progestin–based regimens, including combinations with LNG-IUD or hysteroscopic resection, generally resulted in remission within 3–6 months, while LNG-IUD–based strategies showed wider variability, with reported response times ranging from 4 to 12 months. More pronounced dispersion was observed for combinations involving hysteroscopic resection or metformin. Overall, the substantial heterogeneity in reported time-to-response metrics reflects differences in study design, treatment protocols, and outcome reporting, precluding meaningful quantitative comparisons across treatment strategies and supporting descriptive interpretation only.

#### 4.3.8. Randomized Controlled Trials

Two randomized controlled trials evaluated fertility-sparing treatment strategies in women with FIGO stage IA, grade 1 endometrioid endometrial carcinoma.

Yang et al. [16] compared oral megestrol acetate monotherapy with combined oral megestrol acetate plus metformin. Complete response was achieved in 6 of 9 patients (67%) receiving oral progestins alone and in 12 of 14 patients (86%) treated with oral progestins plus metformin.

Similarly, Xu et al. [17] compared oral megestrol acetate monotherapy with combined oral megestrol acetate plus levonorgestrel-releasing intrauterine device (LNG-IUD). Complete response was observed in 16 of 28 patients (57%) in the oral progestin group and in 16 of 26 patients (62%) in the combined treatment group. Among patients attempting conception after complete response, pregnancy occurred in 13 of 21 patients (62%) and 9 of 12 patients (75%), with corresponding live birth rates of 5 of 21 (24%) and 4 of 12 patients (33%), respectively.

Randomized controlled trials did not report recurrence, partial response, or no response outcomes suitable for quantitative synthesis.

### 4.4. Assessing the Certainty of Evidence

According to the GRADE framework, the certainty of evidence varied across outcomes and treatment strategies, ranging from high to low. Overall, concerns for risk of bias were present, mainly due to the predominance of non-randomized and retrospective study designs, despite generally acceptable methodological quality. The evidence base was further limited by small study populations. Higher certainty ratings were typically assigned to treatment comparisons supported by a larger number of studies and more frequently reported outcomes, whereas lower ratings were observed when sample sizes were small, effect estimates showed minimal between-group differences, or when substantial statistical heterogeneity was present. In line with GRADE guidance, outcomes demonstrating substantial heterogeneity (typically I^2^ > 50%) were downgraded for inconsistency. Complete response, recurrence, pregnancy, and live birth were predefined as critical outcomes, while partial response and no response were classified as important outcomes. (Supplementary Table S1).

### 4.5. Influencing Factors

Patient age and body mass index showed substantial between-study variability across the included literature. No consistent association was identified between higher or lower age or body mass index and complete response rates at the study level. However, interpretation is limited using aggregated data and heterogeneous reporting formats, and potential patient-level effects cannot be excluded. Almost all included patients had grade 1 endometrioid endometrial carcinoma, with grade 2 tumors representing only a small fraction of included women, making it unlikely that tumor grade meaningfully influenced pooled estimates.

Follow-up duration varied widely and was inconsistently reported across studies. Because recurrence was assessed as a proportion among patients achieving complete response rather than as a time-to-event outcome, incorporating follow-up duration into comparative analyses would have introduced substantial bias. Other potentially relevant clinical modifiers, including metabolic status, polycystic ovary syndrome, and treatment adherence, were inconsistently reported and could not be systematically evaluated.

### 4.6. Assessment of Reporting Bias

Funnel plots showed no clear evidence of publication bias for complete response, recurrence, pregnancy, or live birth, with asymmetry consistent with between-study heterogeneity. Interpretation of partial and no-response outcomes was limited by few studies and low event counts. Egger’s test was non-significant for all outcomes except partial response in the overall analysis, while no asymmetry was observed in oral progestin–only analyses. Supplementary Figure S2.

### 4.7. Sensitivity Analysis

Sensitivity analyses were consistent with the primary results across outcomes. Exclusion of large studies or outliers did not materially change pooled estimates but generally reduced heterogeneity, indicating that between-study variability was driven mainly by extreme study sizes or event rates. Partial-response analyses showed greater variability due to sparse events, though estimates remained within a narrow, clinically plausible range. Overall, these findings support the robustness of the main analyses despite limited data in some subgroups (Supplementary Figures S3–S7).

## 5. Discussion

In this systematic review and meta-analysis, we synthesized oncologic and reproductive outcomes following fertility-sparing treatment in women with early-stage endometrial carcinoma, integrating evidence across contemporary conservative strategies, including oral progestins, levonorgestrel-releasing intrauterine devices, hysteroscopic resection, and combination regimens. Although fertility preservation has become an increasingly accepted option for appropriately selected patients, clinical decision-making remains challenging due to the predominance of non-randomized data, heterogeneity in treatment protocols, and the scarcity of direct comparative evidence, particularly for long-term disease control and reproductive endpoints.

Several previous systematic reviews and meta-analyses have evaluated fertility-sparing management by analyzing patients with atypical endometrial hyperplasia and early-stage EC together, implicitly treating these entities as a single clinical continuum. This approach has been adopted in multiple influential reviews, including those by Gallos et al., Wei et al., and De Rocco et al. [10,92,93], as well as in more recent analyses incorporating hysteroscopic resection–based strategies, such as the meta-analysis by Zhao et al. [94] While these studies have substantially advanced the field, the practice of pooling atypical endometrial hyperplasia with endometrial carcinoma introduces important clinical heterogeneity, as highlighted in systematic evaluations of atypical hyperplasia (Sebok et al.) [95], given the distinct biological behavior, malignant potential, and recurrence risk of these conditions. This carcinoma-specific focus distinguishes the present analysis from prior reviews that combined carcinoma and premalignant disease, but may limit direct comparability with studies reporting pooled AEH/EC outcomes.

Importantly, fertility-sparing treatment has also been examined in systematic reviews restricted to EC populations. Recent meta-analyses by Ogunbiyi et al. [96] and Suzuki et al. [97] focused exclusively on patients with early-stage, low-grade EC and provided disease-specific benchmarks for oncologic and reproductive outcomes.

However, these analyses typically evaluated a more limited range of conservative interventions. Building on this literature, the present review incorporates a larger overall patient population and evaluates a broader spectrum of fertility-sparing strategies within an EC-only framework, thereby offering a comprehensive and clinically relevant synthesis of treatment effectiveness across currently used modalities

Within this EC-restricted and methodologically broader context, our findings allow a more granular examination of how both oncologic and reproductive outcomes vary across currently used fertility-sparing strategies.

Overall, fertility-sparing treatment in early-stage endometrial carcinoma was associated with a high pooled complete response rate of 74% (95% CI: 69–79%), confirming that conservative management is generally effective in achieving initial histologic remission, albeit with substantial heterogeneity. Response rates varied by intervention, with combination strategies, particularly those incorporating hysteroscopic resection, consistently demonstrating complete response rates around 85%. Despite these favorable initial outcomes, oncologic outcomes differed markedly across strategies. While recurrence occurred in 35% of complete responders overall, monotherapy approaches, especially oral progestins and LNG-IUD alone, were associated with substantially higher recurrence rates, whereas combination regimens, including hysteroscopic resection–based strategies, showed lower and more consistent recurrence rates, typically ranging between 12% and 16%.

When interpreted alongside previously published EC-focused meta-analyses, our pooled complete response estimate aligns closely with disease-specific benchmarks, although recurrence estimates differ in magnitude depending on the evidence base and endpoint definition. Ogunbiyi et al. [96] (stage IA grade 1 EC) reported an overall remission rate of 77% (95% CI: 70–84%) with a relapse rate of 20% (95% CI: 13–27%). While their remission estimate is only slightly higher than ours, the more substantial divergence is seen in recurrence (their 20% vs. our 35% among CR responders), likely reflecting the smaller underlying dataset in the comparator analysis and therefore less stable pooled estimates.

When stratified by intervention, our results indicate a clinically meaningful gradient in oncologic durability. Oral progestin monotherapy achieved a pooled CR of 72%, yet recurrence remained substantial at 43%, indicating limited long-term disease control despite acceptable initial response rates. LNG-IUD monotherapy demonstrated a lower pooled CR of 59% and the highest pooled recurrence rate at 64%; however, this estimate is based on a small number of heterogeneous studies and should be interpreted cautiously. Importantly, some cohorts contributing to the LNG-IUD monotherapy subgroup, including Hubbs et al. [76], included older patients and women with less favorable baseline characteristics (e.g., higher comorbidity burden or contraindications to systemic progestins). As a result, LNG-IUD monotherapy was often selected as a pragmatic alternative rather than a first-line fertility-sparing strategy, introducing selection bias that likely inflates recurrence rates and attenuates apparent response.

Endpoint selection appears to be an additional driver of between-study differences. The 2024 meta-analysis by Suzuki et al. [97], which focused on the best complete response within 12 months, reported pooled best CR rates of 66% (95% CI: 55–76%) for oral progestins and 86% (95% CI: 69–95%) for LNG-IUD, with corresponding recurrence rates among responders of 31% (95% CI: 22–41%) and 14% (95% CI: 5–31%), respectively. Compared with that time-anchored approach, our analyses yielded a CR of 72% for oral progestins and 59% for LNG-IUD, suggesting that fixed short-term endpoints may overestimate early response, particularly for LNG-IUD–based strategies, without fully capturing longer-term disease control.

In contrast, combination strategies incorporating hysteroscopic resection (HR) consistently demonstrated more favorable oncologic profiles. Oral progestin plus HR achieved a pooled CR of 85% with a recurrence rate of 16%, while LNG-IUD plus HR yielded a pooled CR of 85% and a recurrence rate of 14%, supporting the hypothesis that surgical cytoreduction improves durability by reducing tumor burden and enhancing local progesterone responsiveness. This pattern is concordant with prior meta-analytic literature: Ogunbiyi et al. [96] reported a pooled remission rate of 84% and a relapse rate of 9.3% for hysteroscopic resection combined with adjuvant progestin, compared with a relapse rate of 28% for oral progestin monotherapy, and Zhao et al. [94] reported a pooled CR of 88.6% (95% CI: 84.8–92.0%) with a pooled recurrence rate of 18.3% (95% CI: 13.7–23.3%) for hysteroscopic resection–based conservative therapy in early-stage EC. Together, these findings suggest that cytoreductive strategies mainly reduce relapse after an initial response, rather than increasing the probability of achieving response per se.

Other combination regimens showed high initial response rates but substantial variability. Oral progestin combined with LNG-IUD resulted in a pooled CR of 72% and a recurrence rate of 12%, although derived from very limited data. LNG-IUD combined with GnRHa achieved a pooled CR of 83% with a recurrence rate of 12%, suggesting potentially favorable oncologic control in selected cohorts, albeit with heterogeneous indications. Oral progestin combined with metformin showed a pooled CR of 80%, but extreme heterogeneity (I^2^ = 96%) limited robustness. Consistent with these findings, Fernandez-Montoli et al. [98] reported low-certainty evidence that metformin combined with progestin may modestly increase complete response (RR 1.85, 95% CI 1.07–3.19) while having little to no effect on live birth (RR 1.80, 95% CI 0.88–3.68).

Beyond complete response and recurrence, early treatment failure was clinically relevant. In early-stage endometrial carcinoma, pooled partial response and no response rates were 6% and 16%, respectively, identifying a subset unlikely to benefit from prolonged fertility-sparing treatment. No response rates were higher with monotherapy (21% for oral progestins, 23% for LNG-IUD) than with combination regimens (around 12%), except for LNG-IUD plus GnRHa (21%), while partial response rates were generally low (1–10%). Overall, these results indicate that combination approaches, particularly those including hysteroscopic resection, primarily reduce treatment failure and relapse rather than substantially increasing complete response rates, enabling earlier identification of ineffective therapy.

From a reproductive perspective, fewer than half of women treated with fertility-sparing strategies achieved pregnancy, with a pooled pregnancy rate of 48%, and only approximately one-third achieved a live birth, reflected by a pooled live birth rate of 36%. This consistent gap between oncologic remission and reproductive success underscores, that histologic response alone does not guarantee favorable fertility outcomes. Pregnancy did not uniformly translate into live birth, emphasizing the divergence between oncologic and reproductive outcomes.

Intervention-stratified analyses revealed consistent and clinically meaningful patterns. Oral progestin monotherapy was associated with pooled pregnancy and live birth rates of 43% and 35%, respectively. LNG-IUD monotherapy demonstrated a higher pooled pregnancy rate of 64%, but a similar live birth rate of 33%, although these estimates were characterized by wide confidence intervals due to small sample sizes. In contrast, combination approaches, including oral progestin combined with LNG-IUD or hysteroscopic resection, were associated with more consistent and generally higher reproductive outcomes, with pregnancy rates around 57–58% and live birth rates of 43–44%. In smaller subgroups, LNG-IUD combined with hysteroscopic resection achieved a pooled pregnancy rate of 75% alongside strong oncologic efficacy (CR 85%), although interpretation is limited by the small number of contributing studies. Conversely, LNG-IUD combined with GnRHa was associated with comparatively poorer reproductive outcomes, with a pooled pregnancy rate of 31% and a live birth rate of 20%, despite high oncologic response rates, suggesting a dissociation between tumor regression and reproductive potential with this regimen.

Although this review incorporated two randomized controlled trials, randomized evidence remains limited in scope. The available trials contributed data only to complete response and reproductive outcomes, while recurrence, partial response, and no response endpoints were not reported in the randomized trials. Consequently, pooled estimates for oncologic durability outcomes were derived exclusively from cohort studies [16,17].

These findings are broadly concordant with prior EC-focused meta-analyses, while also highlighting important methodological differences. Ogunbiyi et al. reported that despite an overall remission rate of 77% (95% CI: 70–84%), the pooled live birth proportion was only 20% (95% CI: 15–25%), whereas Zhao et al. observed live birth rates of 26.0% (95% CI: 17.3–35.5%) among all treated patients and 30.6% (95% CI: 21.0–41.0%) among complete responders undergoing hysteroscopic resection-based conservative therapy. In contrast, in the present analysis, reproductive outcomes were calculated using as denominator women who achieved complete response and subsequently attempted conception, rather than all women initiating fertility-sparing treatment or all complete responders regardless of reproductive intent. This difference in outcome definition likely contributes to discrepancies in reported pregnancy and live birth rates across meta-analyses and underscores the importance of distinguishing treatment efficacy from realized reproductive intent.

Overall, fertility-sparing management in early-stage endometrial carcinoma should be viewed as a time-limited opportunity to achieve pregnancy rather than as a definitive oncologic solution. In this context, monotherapy refers to the use of a single conservative modality, most commonly oral progestins or a levonorgestrel-releasing intrauterine device alone, whereas combination therapies encompass multimodal approaches that integrate hormonal treatment with additional interventions, such as hysteroscopic tumor resection, combined systemic and intrauterine progestins, or the addition of agents such as metformin or gonadotropin-releasing hormone analogs. Although statistically significant differences between treatment strategies were not consistently demonstrated across pooled analyses, the available evidence suggests clinically meaningful directional trends, with combination approaches generally associated with higher complete response rates and greater durability of remission compared with hormonal monotherapy. Nevertheless, the substantial risk of disease recurrence despite initial response underscores the importance of early fertility counseling, close oncologic surveillance, and a timely transition to definitive surgical management once childbearing goals have been achieved.

### 5.1. Strengths and Limitations

This systematic review and meta-analysis provides a focused and disease-specific synthesis of fertility-sparing management in early-stage endometrial carcinoma, deliberately excluding atypical hyperplasia to avoid dilution of oncologic and reproductive outcomes. Although several included studies enrolled mixed populations of endometrial carcinoma and atypical endometrial hyperplasia or endometrial intraepithelial neoplasia, only carcinoma-specific outcomes were included in the quantitative synthesis. This approach was chosen to preserve clinical homogeneity and to avoid conflating biologically and prognostically distinct disease entities. This restriction improves clinical interpretability and ensures that pooled estimates more accurately reflect the biologic behavior, recurrence risk, and fertility trade-offs of malignant disease. A further strength is the comprehensive evaluation of a wide range of fertility-sparing strategies, including progestin-based monotherapies, LNG-IUD-based regimens, hysteroscopic resection, and combination approaches, allowing intervention-specific comparisons across key oncologic and reproductive endpoints. Importantly, the analysis extends beyond complete response and recurrence to include early failure patterns and reproductive outcomes, which are essential for patient counseling and clinical decision-making but remain underreported in prior meta-analyses. Methodological rigor was reinforced through protocol preregistration, adherence to PRISMA guidelines, advanced multilevel modeling, and sensitivity analyses demonstrating that pooled estimates were robust to exclusion of large cohorts or statistical outliers.

The interpretation of these findings requires careful consideration of several limitations related to the nature of the available evidence. First, the majority of included studies were retrospective and non-randomized cohort analyses, and treatment allocation was commonly influenced by physician preference, institutional protocols, and patient characteristics, which limits the ability to infer causal relationships. Although random-effects models were applied to account for between-study variability, substantial statistical heterogeneity was observed across most pooled analyses, reflecting differences in eligibility criteria, baseline risk profiles, therapeutic regimens, surveillance strategies, and follow-up duration among the included cohorts.

In this context, pooled effect estimates should be interpreted as average effects derived from heterogeneous clinical settings rather than as precise comparative measures applicable to individual patients. To improve the clinical interpretability of these findings despite heterogeneity, prespecified subgroup analyses were conducted according to treatment modality, and multiple sensitivity analyses were performed, including the exclusion of very large cohorts and statistical outliers. These additional analyses yielded broadly consistent directions of effect, supporting the robustness of the main findings while acknowledging residual uncertainty.

Meta-regression analyses were not undertaken, as several clinically relevant effect modifiers, including metabolic status, infertility history, reproductive intent, use of assisted reproductive technologies, and molecular tumor characteristics, were inconsistently reported or entirely unavailable across most primary studies, rendering multivariable modeling unreliable and prone to spurious associations.

Reproductive outcomes were associated with further sources of variability, particularly in retrospective cohorts, where documentation of pregnancy intention, duration of attempts to conceive, and utilization of assisted reproductive technologies was often incomplete. When attempt-specific denominators could not be ascertained, reproductive outcomes were calculated among women achieving complete response, a strategy that enhances clinical relevance but limits generalizability to the broader treated population. Moreover, some treatment subgroups comprised relatively small numbers of patients, further reducing statistical precision. Consequently, findings derived from smaller subgroups should be regarded as hypothesis-generating rather than definitive, highlighting the need for larger, prospectively designed comparative studies.

In this context, although pooled analyses suggested favorable outcomes for certain metformin- and GnRHa-based combination therapies, these findings were derived from a limited number of studies and were characterized by substantial heterogeneity. Accordingly, the current evidence is insufficient to support firm clinical recommendations for these strategies, which should be considered exploratory and require confirmation in adequately powered, prospective studies with standardized protocols.

Taken together, these considerations indicate that the present meta-analysis provides a clinically informative synthesis of fertility-sparing management in early-stage endometrial carcinoma, while underscoring the need for prospective, disease-specific studies with standardized protocols and long-term follow-up.

### 5.2. Future Directions

Future research should focus on EC-specific, comparative studies with standardized eligibility, surveillance, and outcome definitions to clarify differences in response induction and durability across fertility-sparing strategies. Consistent reporting of recurrences, reproductive outcomes, and key clinical modifiers, ideally through prospective trials or individual-level analyses, will be essential to generate actionable, patient-centered evidence.

## 6. Conclusions

In this systematic review and meta-analysis focused on early-stage endometrial carcinoma, fertility-sparing treatment achieved high initial histologic remission but showed limited durability and moderate reproductive success. Recurrence among complete responders was frequent, supporting the concept of conservative management as a time-limited strategy requiring close surveillance and definitive surgery after childbearing. While statistically significant differences between approaches were not demonstrated, combination therapies were generally associated with more favorable oncologic outcomes and, to a lesser extent, higher live birth rates than monotherapy. Within these, hysteroscopic resection-based strategies appeared particularly advantageous, whereas monotherapy was more often linked to early treatment failure.

## Figures and Tables

**Table 1:** Basic characteristics of included studies: oral progestin monotherapy and LNG-IUD monotherapy.

Study	No. of Patients	Age ^1^	BMI ^2^	Therapy ^3^	Regimen ^4^	CR ^5^	Recurrence Rate ^6^	Pregnancy Rate ^7^	Live Birth Rate ^8^	Partial Response ^9^	No Response ^10^
**Oral progestin**											
Ohyagi-Hara C. 2015 [49]	16	34.2 (10.1) M	24 (16–47.3) MD	Oral prog.	MPA 400–600 mg	11/16	9/11	1/8	1/8	0/16	5/16
Andress J. 2021 [48]	11	32.2 (30–48) MD	33.1 (21–48) MD	Oral prog.	MA 160 mg, MPA 500 mg	5/11	N/A	N/A	N/A	4/11	2/11
Kaku T. 2001 [47]	12	29.3 (21–42) MD	N/A	Oral prog.	MPA 100–800 mg	9/12	2/9	2/7	1/7	0/12	3/12
Tamauchi S. 2024 [52]	23	34.2 (19.4–44.6) MD	24.5 (18–45) MD	Oral prog.	MPA 600 mg	19/23	11/19	5/11	5/11	0/23	4/23
Baek J.S. 2016 [54]	13	33 M	23.1 M	Oral prog.	MA 80–160 mg, MPA 40–160 mg	7/13	4/7	N/A	N/A	N/A	N/A
Roh H.J. 2021 [55]	18	32.1 (19–40) M	27.2 (14.9–38) M	Oral prog.	MA 40–320 mg, MPA 10–400 mg	8/18	N/A	N/A	N/A	1/18	9/18
Niwa K. 2005 [22]	12	31 (23–34) MD	22 (17–40) MD	Oral prog.	MPA 400–600 mg	12/12	8/12	7/10	5/10	0/12	0/12
Gotlieb W.H. 2003 [57]	13	31 (23–40) MD	N/A	Oral prog.	MA 160 mg, MPA 200–600 mg, NA 5 mg	13/13	6/13	3/13	N/A	0/13	0/13
Xu Z. 2023 [17]	28	30 (21–43) MD	25 (18–52) MD	Oral prog.	MA 160 mg	16/28	N/A	13/16	5/16	N/A	N/A
Chae S.H. 2019 [59]	118	37 (28–45) MD	22.7 (18.5–43.5) MD	Oral prog.	MPA 500–1000 mg	71/118	N/A	22/49	N/A	N/A	N/A
Park J.Y. 2013 [61]	148	31.3 (21–40) M	25 (15–38) M	Oral prog.	MA 40–240 mg, MPA 30–1500 mg	115/148	35/115	44/115	N/A	N/A	N/A
Park H. 2012 [62]	14	30 M	22.3 M	Oral prog.	MA 160–240, MPA 30–500 mg	13/14	2/13	4/7	N/A	N/A	N/A
Hahn H.S. 2009 [63]	35	31 (21–43) MD	N/A	Oral prog.	MA 160 mg, MPA 250–1500 mg	22/35	9/22	10/12	8/12	1/35	12/35
Eftekhar Z. 2009 [25]	21	29 (21–45) MD	29.3 M	Oral prog.	MA 160–320 mg	18/21	3/18	5/18	2/18	N/A	N/A
Ou Y.C. 2021 [65]	45	32.4 (23–42) MD	29 (16–55) MD	Oral prog.	MA, MPA	41/45	N/A	9/23	12/23	N/A	N/A
Park J. 2013 [66]	48	30 (23–40) MD	23.6 (18.57–38.20) MD	Oral prog.	MA 160 (40–240, MPA 30–1500 mg	37/48	16/37	12/22	12/22	N/A	N/A
Shirali E. 2012 [26]	16	32 (24–42) MD	30 (23–34) MD	Oral prog.	MA 160–320 mg	10/16	N/A	4/9	N/A	N/A	N/A
Chen M. 2016 [68]	37	N/A	32 (21–41) MD	Oral prog.	MA 160–480 mg, MPA 250–500 mg, GnRHa	27/37	8/27	8/27	5/27	3/37	7/37
Ouasti S. 2022 [30]	30	35 (19–47) MD	N/A	Oral prog.	CA 10 mg	23/30	N/A	N/A	N/A	N/A	N/A
Ushijima K. 2007 [31]	22	31.7 (20–39) MD	22.8 (16–33) MD	Oral prog.	MPA 600 mg	14/22	8/14	4/14	3/14	N/A	N/A
Yang B.Y. 2020 [16]	9	N/A	N/A	Oral prog.	MA 160 mg,	6/9	N/A	N/A	N/A	N/A	N/A
Chen J. 2022 [29]	22	33.5 (30–46) MD	32 (21–42) MD	Oral prog.	MPA 500 mg, MA 160 mg	15/22	5/15	3/7	1/7	N/A	N/A
Koskas M. 2012 [70]	8	34.4 M	N/A	Oral prog.	MPA, MA, NA, CA	5/8	2/5	2/5	2/5	0/8	3/8
Bokhman J.V. 1985 [34]	19	28.0 M	N/A	Oral prog.	Oxyprogesterone-caproate 500 mg	15/19	0/15	N/A	N/A	N/A	N/A
Cade T.J. 2010 [71]	4	33.5 M	N/A	Oral prog.	MA 200 mg	2/4	N/A	0/2	0/2	N/A	N/A
Duska L.R. 2001 [73]	12	30.5 M	24 M	Oral prog.	N/A	10/12	N/A	4/10	N/A	N/A	N/A
Fujiwara H. 2012 [74]	59	31 (21–42) MD	23.3 (15–38) M	Oral prog.	MPA 400–600 mg	42/59	22/42	18/42	18/42	N/A	N/A
Fukui Y. 2017 [75]	35	33 (19–39) MD	21.5 (17.7–34.9) MD	Oral prog.	MPA 600 mg	25/35	8/25	12/25	11/25	N/A	N/A
Hubbs J. 2013 [76]	16	70 (22–89) MD	40 (23.2–77.6)	Oral prog.	N/A	4/16	3/4	N/A	N/A	N/A	N/A
Jafari Shobeiri M. 2013 [35]	8	30 M	N/A	Oral prog.	MA 320 mg	7/8	3/7	3/7	2/7	N/A	N/A
Yuan F. 2022 [36]	60	35.12 (8.41) M	33.37 (4.5) M	Oral prog.	MPA	12/60	N/A	N/A	N/A	20/60	28/60
Yang Y.C. 2005 [38]	6	32.5 M	22.6 M	Oral prog.	MA 160 mg	2/6	N/A	N/A	N/A	N/A	N/A
Wang C.B. 2002 [39]	9	32.9 M	N/A	Oral prog.	MA 160 mg	8/9	4/8	4/8	2/8	0/9	1/9
Mao Y. 2010 [78]	6	28.3 M	N/A	Oral prog.	MA 160 mg, MPA 250–500 mg	4/6	0/4	3/4	3/4	N/A	N/A
Mentrikoski J. 2012 [79]	2	32 (18–42) MD	N/A	Oral prog.	N/A	0/2	N/A	N/A	N/A	N/A	N/A
Minaguchi T. 2007 [80]	19	31 M	N/A	Oral prog.	MPA 400–600 mg	15/19	5/15	3/15	3/15	0/19	4/19
Ota T. 2005 [81]	12	31 M	N/A	Oral prog.	MPA 600 mg	5/12	2/5	N/A	N/A	0/12	7/12
Mazzon I. 2010 [40]	6	32 M	22.17 M	Oral prog.	N/A	6/6	3/6	4/6	4/6	0/6	0/6
Novikova O.V. 2021 [33]	27	24.2 M	34 (20–46) MD	Oral prog.	MPA 500 mg	22/27	N/A	N/A	N/A	N/A	N/A
Dagher C. 2023 [89]	2	37.5 M	22.8 M	Oral prog.	MA 160 mg	1/2	N/A	1/1	1/1	N/A	N/A
Wang C.J. 2014 [67]	37	32 (18–40) MD	24.9 (17.9 to 44.9) MD	Oral prog.	MA 160 mg	30/37	15/30	4/15	4/15	1/37	6/37
Ushijima K. 2023 [82]	177	35 (19–44) MD	24.5 (15.2–50.8) MD	Oral prog.	MPA	132/177	N/A	N/A	N/A	N/A	N/A
Rossetti D. 2014 [42]	5	30 (27–31) MD	22 (20–23) MD	Oral prog.	MA 160 mg	5/5	2/5	5/5	5/5	N/A	N/A
Wheeler D.T. 2007 [83]	16	N/A	N/A	Oral prog.	N/A	5/16	N/A	N/A	N/A	N/A	N/A
Yamazawa K. 2007 [43]	9	35.5 M	N/A	Oral prog.	MPA 400 mg	7/9	N/A	4/7	3/7	2/9	0/9
Signorelli M. 2009 [44]	11	32 (21–40) MD	27.7 (19–41) MD	Oral prog.	Progesterone 200 mg	2/11	N/A	N/A	N/A	4/11	5/11
Perri T. 2011 [85]	27	33.4 (5.4) M	N/A	Oral prog.	NA 5 mg, Hydroxyprogesterone caproate, MPA 100–200 mg	24/27	15/24	14/24	11/24	2/27	1/27
Yahata T. 2006 [86]	8	31.8 M	25.4 M	Oral prog.	MPA	7/8	7/7	3/7	2/7	N/A	N/A
Parlakgumus H.A. 2014 [87]	5	35 M	N/A	Oral prog.	MA 80–160 mg	5/5	N/A	2/5	1/5	0/5	0/5
Kim Y.B. 1997 [88]	7	32.4 M	N/A	Oral prog.	MA 160 mg	4/7	1/4	0/4	0/4	N/A	N/A
Yamagami W. 2018 [91]	97	35 (19–44) MD	21.9 (15.8–41.4) MD	Oral prog.	MPA 400–600 mg	88/97	55/88	20/88	N/A	N/A	N/A
**LNG-IUD**											
Fang F. 2021 [19]	25	30 M	N/A	LNG-IUD	LNG	15/25	N/A	7/15	3/15	3/25	7/25
Leone Roberti Maggiore U. 2019 [53]	20	33.8 M	24.5 M	LNG-IUD	LNG	16/20	8/16	8/8	6/8	1/20	3/20
Falcone F. 2020 [56]	4	34.75 M	26.25 M	LNG-IUD	LNG	4/4	4/4	N/A	N/A	0/4	0/4
Cade T.J. 2010 [71]	3	44 M	N/A	LNG-IUD	LNG	1/3	N/A	0/1	0/1	N/A	N/A
Catena U. 2022 [72]	3	35.6 M	26.72 M	LNG-IUD	LNG	2/3	2/2	0/1	0/1	0/3	1/3
Hubbs J. 2013 [76]	23	37 (22–83) MD	50.6 (28.8–67.8) MD	LNG-IUD	LNG	10/23	7/10	1/1	N/A	N/A	N/A
Mentrikoski J. 2012 [79]	6	32 (18–42) MD	N/A	LNG-IUD	LNG	4/6	0/4	N/A	N/A	N/A	N/A
Wheeler D.T. 2007 [83]	5	N/A	N/A	LNG-IUD	LNG	2/5	N/A	N/A	N/A	N/A	N/A
Dagher C. 2023 [89]	4	35.5 M	35.6 M	LNG-IUD	LNG	3/4	2/3	1/2	1/2	0/4	1/4
Pal N. 2018 [90]	17	45.6 (18.5–85.2) MD	45 (20–74) MD	LNG-IUD	LNG	5/17	N/A	N/A	N/A	5/17	7/17

Basic characteristics of included studies: oral progestin monotherapy and LNG-IUD monotherapy. ^1^ Age is measured in years, M = mean, m = median, ^2^ BMI is measured as (kg/m^2^), M = mean, MD = median, ^3^ Therapeutic methods are Oral = oral progestins, IUD = intrauterine device, GnRHa = gonadotropin releasing hormone analog, HR = Hysteroscopic resection, Met = metformin, ^4^ Therapeutic regimens are MA = Megestrol acetate, MPA = Medroxyprogesterone acetate, LNG = Levonorgestrel, NA = Norethisterone acetate, CA = Clomiphene acetate, N/A = Data not available ^5^ CR = complete response, ^6^ Recurrence rate, ^7^ Pregnancy rate, ^8^ Live birth rate are calculated from the patients reached CR, ^9^ Partial response, ^10^ No response.

**Table 2:** Basic characteristics of included studies: combination fertility-sparing therapies.

Study	No. of Patients	Age ^1^	BMI ^2^	Therapy ^3^	Regimen ^4^	CR ^5^	Recurrence Rate ^6^	Pregnancy Rate ^7^	Live Birth Rate ^8^	Partial Response ^9^	No Response ^10^
**Oral progestin plus hysteroscopic resection**											
Ayhan A. 2020 [46]	30	32 MD	30.8 M	Oral + HR	MA 160 mg	22/30	5/22	8/17	5/17	1/30	7/30
Shan B.E. 2013 [18]	14	30 (18–38) MD	21.3 (17–38) MD	Oral + HR	MA 160 mg	11/14	3/11	2/8	1/8	0/14	3/14
Casadio P. 2020 [50]	36	33.1 M	29.1 M	Oral + HR	MA 160 mg	34/36	4/34	21/34	14/34	N/A	N/A
Falcone F. 2020 [56]	5	32.6 M	26.44 M	Oral + HR	MA 160 mg, NA 10 mg	4/5	1/4	2/4	2/4	0/4	1/4
Kuang J. 2021 [58]	65	30 (25–39) MD	27 M	Oral + HR	MA 160 mg	29/65	N/A	N/A	N/A	25/65	11/65
Wang F. 2017 [60]	11	27.3 (25–39) MD	23.6 (18–28) MD	Oral + HR	MA 160–320 mg, MPA 250–500 mg	9/11	N/A	6/9	6/9	2/11	0/11
Falcone F. 2017 [27]	6	38 M	26.6 M	Oral + HR	MA 160 mg, NA 10 mg	6/6	N/A	3/3	3/3	0/6	0/6
Yang H.C. 2019 [37]	6	34.5 M	29.1	Oral + HR	MA 160 mg, MPA 500 mg	6/6	N/A	N/A	N/A	N/A	N/A
Wang Q. 2015 [41]	6	29.5 M	20.39 M	Oral + HR	MA 160–320 mg	6/6	N/A	N/A	N/A	0/6	0/6
**Oral progestin plus LNG-IUD**											
Fang F. 2021 [19]	31	30 M	N/A	Oral prog. + LNG-IUD	MPA 10 mg	27/31	N/A	13/20	11/20	2/31	2/31
Hwang J.Y. 2017 [77]	5	30.4 M	24 M	Oral prog. + LNG-IUD	MA 160 mg, MPA 250–1500 mg	3/5	1/3	1/3	0/3	2/5	0/5
Roh H.J. 2021 [55]	25	32.1 (19–40) M	27.2 (14.9–38) M	Oral + LNG-IUD	LNG, MA 40–320 mg, MPA 10–400 mg	23/25	N/A	N/A	N/A	2/25	0/25
Xu Z. 2023 [17]	26	30 (21–43) MD	25 (18–52) MD	Oral + LNG-IUD	MA 160 mg, LNG	16/26	N/A	9/12	4/12	N/A	N/A
Kim M.K. 2019 [23]	35	32.9 (27–40) MD	24.5 (15–37) MD	Oral + LNG-IUD	MPA 500 mg	13/35	N/A	N/A	N/A	9/35	13/35
Kim M.K. 2013 [28]	16	34.8 M	24.4 M	Oral + LNG-IUD	MPA 500 mg	14/16	2/14	3/9	N/A	1/16	1/16
Cade T.J. 2010 [71]	9	32.7 M	N/A	Oral + LNG-IUD	MA 200 mg, LNG-IUD	7/9	N/A	4/7	3/7	N/A	N/A
Pino I. 2022 [84]	20	34.5 (6.4) M	23.8 (4.5) M	Oral + LNG-IUD	LNG, MA 160 mg	16/20	N/A	5/10	5/10	1/20	3/20
Dagher C. 2023 [89]	7	31.2 M	29.4 M	Oral + LNG-IUD	LNG, MA 160 mg	2/7	1/2	1/2	1/2	0/7	5/7
Kim M.K. 2011 [45]	5	38.4 (4.8) M	20.3 (8.9) M	Oral + LNG-IUD	LNG, MPA 500 mg	4/5	0/4	N/A	N/A	1/5	0/5
**LNG-IUD plus hysteroscopic resection**											
Laurelli G. 2016 [24]	21	35.9 M	28.5 M	LNG-IUD + HR	LNG	19/21	2/19	11/12	N/A	0/21	2/21
Falcone F. 2020 [56]	12	34.6 M	30.4 M	LNG-IUD + HR	LNG, MA 160 mg, NA 10 mg	8/12	3/8	1/5	1/5	0/12	4/12
Giampaolino P. 2019 [51]	14	35.1 M	25.9 M	LNG-IUD + HR	LNG	11/14	2/11	N/A	N/A	N/A	N/A
Falcone F. 2017 [27]	22	37.4 M	28.2 M	LNG-IUD + HR	LNG	20/22	2/20	11/12	10/12	1/22	1/22
**LNG-IUD plus GnRHa**											
Minig L. 2011 [20]	14	34 (22–40) MD	21 (17–41) MD	LNG.IUD + GnRHa	LNG, GnRHa	8/14	2/8	1/8	1/8	0/14	6/14
Pashov A.I. 2012 [21]	11	30.2 M	N/A	LNG-IUD + GnRHa	LNG, GnRHa	11/11	N/A	3/11	2/11	0/11	0/11
Chen J. 2022 [29]	40	33.5 (30–46) MD	32 (21–42) MD	LNG-IUD + GnRHa	LNG, GnRHa	37/40	5/37	7/27	2/27	N/A	N/A
Pronin S.M. 2015 [32]	32	33 M	N/A	LNG-IUD + GnRHa	LNG, GnRHa	23/32	2/23	N/A	N/A	N/A	N/A
Novikova O.V. 2021 [33]	139	24.2 M	34 (20–46) MD	LNG-IUD + GnRHa	LNG, GnRHa	128/139	N/A	N/A	N/A	N/A	N/A
Pino I. 2022 [84]	31	32.8 (4.9) M	25.8 (8.1) M	LNG-IUD + GnRHa	LNG, Triptorelin Acetate	20/31	N/A	8/14	7/14	2/31	9/31
**Oral progestin plus Metformin**											
Jing C.Y. 2022 [64]	79	30 (27–33) MD	23.4 (20.0–27.5) MD	Oral prog. + Metformin	MA 160 mg, Met 1500 mg	75/79	26/75	29/58	27/58	N/A	N/A
Yang B.Y. 2020 [16]	14	N/A	N/A	Oral prog. + Metformin	MA 160 mg, Met 1500 mg	12/14	N/A	N/A	N/A	N/A	N/A
Mitsuhashi A. 2019 [69]	42	35 (26–44) MD	31 (15.4–50.8) MD	Oral + Metformin	MA 160 mg, Met 750–2250 mg	40/42	7/40	N/A	N/A	0/42	2/42
Yuan F. 2022 [36]	60	33.73 (7.5) M	34.4 (4.2) M	Oral prog. + Metformin	MPA, Met	16/60	N/A	N/A	N/A	27/60	17/60
Ushijima K. 2023 [82]	24	35 (19–44) MD	24.5 (15.2–50.8) MD	Oral prog. + Metformin	MPA, Met	20/24	N/A	N/A	N/A	N/A	N/A
**Oral progestin plus LNG-IUD plus GnRHa**											
Novikova O.V. 2021 [33]	24	24.2 M	34 (20–46) MD	Oral + LNG-IUD + GnRHa	MPA 500 mg, LNG, GnRHa	17/24	N/A	N/A	N/A	N/A	N/A
Pino I. 2022 [84]	24	36.3 M	25.6 M	Oral + LNG-IUD + GnRHa	MA 160 mg, LNG, Triptorelin Acetate	20/24	N/A	N/A	N/A	1/24	3/24

Basic characteristics of included studies: combination fertility-sparing therapies ^1^ Age is measured in years, M = mean, m = median, ^2^ BMI is measured as (kg/m^2^), M = mean, MD = median, ^3^ Therapeutic methods are Oral = oral progestins, IUD = intrauterine device, GnRHa = gonadotropin releasing hormone analog, HR = Hysteroscopic resection, Met = metformin, ^4^ Therapeutic regimens are MA = Megestrol acetate, MPA = Medroxyprogesterone acetate, LNG = Levonorgestrel, NA = Norethisterone acetate, CA = Clomiphene acetate, N/A = Data not available ^5^ CR = complete response, ^6^ Recurrence rate, ^7^ Pregnancy rate, ^8^ Live birth rate are calculated from the patients reached CR, ^9^ Partial response, ^10^ No response.

## Data Availability

This study is based exclusively on publicly available data; no new data were generated or analyzed in support of this research. Upon reasonable request, we are happy to provide any further information regarding the preparation of this manuscript.

## Supplementary Materials

The following supporting information can be downloaded at: https://www.mdpi.com/article/10.3390/cancers18030399/s1, Figure S1: Risk of Bias assessment; Figure S2: Funnel plots; Figures S3–S7: Sensitivity analyses; Table S1: GRADE Summary of Findings; Table S2: PRISMA 2000 for Abstracts Checklist; Table S3: PRISMA 2000 Checklist; Table S4: List of excluded studies. References [24,71,85,99,100,101,102,103,104,105,106,107,108,109,110,111,112,113,114,115,116,117,118,119,120,121,122,123,124,125,126,127,128,129,130,131,132,133,134,135,136,137,138,139,140,141,142,143,144,145,146,147,148,149,150,151,152,153,154,155,156,157,158,159,160,161,162,163,164,165,166,167,168,169] are cited in Supplementary Materials.

## Abbreviations

The following abbreviations are used in this manuscript:

CR	Complete response
EC	Endometrial carcinoma
FST	Fertility-sparing treatment
GnRHa	Gonadotropin-releasing hormone analog
HR	Hysteroscopic resection
LBR	Live birth rate
LNG-IUD	Levonorgestrel-releasing intrauterine device
MA	Megestrol acetate
MPA	Medroxyprogesterone acetate
NR	No response
OP	Oral progestin
PR	Partial response
RCT	Randomized controlled trial
RR	Recurrence rate

## References

[1] Crosbie E.J., Kitson S.J., McAlpine J.N., Mukhopadhyay A., Powell M.E., Singh N. (2022). Endometrial cancer. Lancet.

[2] Bassette E., Ducie J.A. (2024). Endometrial Cancer in Reproductive-Aged Females: Etiology and Pathogenesis. Biomedicines.

[3] Concin N., Matias-Guiu X., Cibula D., Colombo N., Creutzberg C.L., Ledermann J., Mirza M.R., Vergote I., Abu-Rustum N.R., Bosse T. (2025). ESGO-ESTRO-ESP guidelines for the management of patients with endometrial carcinoma: Update 2025. Lancet Oncol..

[4] Ronsini C., Romeo P., Andreoli G., Palmara V., Palumbo M., Caruso G., De Franciscis P., Vizzielli G., Restaino S., Chiantera V. (2025). Fertility-Sparing Treatments in Endometrial Cancer: A Comprehensive Review on Efficacy, Oncological Outcomes, and Reproductive Potential. Medicina.

[5] Concin N., Matias-Guiu X., Vergote I., Cibula D., Mirza M.R., Marnitz S., Ledermann J., Bosse T., Chargari C., Fagotti A. (2021). ESGO/ESTRO/ESP guidelines for the management of patients with endometrial carcinoma. Int. J. Gynecol. Cancer.

[6] Rodolakis A., Scambia G., Planchamp F., Acien M., Di Spiezio Sardo A., Farrugia M., Grynberg M., Pakiz M., Pavlakis K., Vermeulen N. (2023). ESGO/ESHRE/ESGE Guidelines for the fertility-sparing treatment of patients with endometrial carcinoma. Hum. Reprod. Open.

[7] Kim J.J., Kurita T., Bulun S.E. (2013). Progesterone action in endometrial cancer, endometriosis, uterine fibroids, and breast cancer. Endocr. Rev..

[8] Leipold G., Toth R., Harsfalvi P., Loczi L., Torok M., Keszthelyi A., Acs N., Lintner B., Varbiro S., Keszthelyi M. (2024). Comprehensive Evaluation of a Levonorgestrel Intrauterine Device (LNG-IUD), Metformin, and Liraglutide for Fertility Preservation in Endometrial Cancer: Protocol for a Randomized Clinical Trial. Life.

[9] Emons G., Grundker C. (2021). The Role of Gonadotropin-Releasing Hormone (GnRH) in Endometrial Cancer. Cells.

[10] Gallos I.D., Yap J., Rajkhowa M., Luesley D.M., Coomarasamy A., Gupta J.K. (2012). Regression, relapse, and live birth rates with fertility-sparing therapy for endometrial cancer and atypical complex endometrial hyperplasia: A systematic review and metaanalysis. Am. J. Obstet. Gynecol..

[11] Mitsuhashi A., Kiyokawa T., Sato Y., Shozu M. (2014). Effects of metformin on endometrial cancer cell growth in vivo: A preoperative prospective trial. Cancer.

[12] Page M.J., McKenzie J.E., Bossuyt P.M., Boutron I., Hoffmann T.C., Mulrow C.D., Shamseer L., Tetzlaff J.M., Akl E.A., Brennan S.E. (2021). The PRISMA 2020 statement: An updated guideline for reporting systematic reviews. BMJ.

[13] Higgins J., Thomas J., Chandler J., Cumpston M., Li T., Page M., Welch V. (2024). Cochrane Handbook for Systematic Reviews of Interventions, Version 6.5.

[14] PROSPERO International Prospective Register of Systematic Reviews Efficacy of Fertility-Sparing Treatments in Early-Stage Endometrial Carcinoma: Oncologic and Reproductive Outcomes. https://www.crd.york.ac.uk/PROSPERO/view/CRD420251032161.

[15] Schünemann H., Brożek J., Guyatt G., Oxman A. (2013). GRADE Handbook for Grading Quality of Evidence and Strength of Recommendations.

[16] Yang B.Y., Gulinazi Y., Du Y., Ning C.C., Cheng Y.L., Shan W.W., Luo X.Z., Zhang H.W., Zhu Q., Ma F.H. (2020). Metformin plus megestrol acetate compared with megestrol acetate alone as fertility-sparing treatment in patients with atypical endometrial hyperplasia and well-differentiated endometrial cancer: A randomised controlled trial. BJOG.

[17] Xu Z., Yang B., Guan J., Shan W., Liao J., Shao W., Chen X. (2023). Comparison of the effect of oral megestrol acetate with or without levonorgestrel-intrauterine system on fertility-preserving treatment in patients with early-stage endometrial cancer: A prospective, open-label, randomized controlled phase II trial (ClinicalTrials.gov NCT03241914). J. Gynecol. Oncol..

[18] Shan B.E., Ren Y.L., Sun J.M., Tu X.Y., Jiang Z.X., Ju X.Z., Zang R.Y., Wang H.Y. (2013). A prospective study of fertility-sparing treatment with megestrol acetate following hysteroscopic curettage for well-differentiated endometrioid carcinoma and atypical hyperplasia in young women. Arch. Gynecol. Obstet..

[19] Fang F., Xu H., Wu L., Hu L., Liu Y., Li Y., Zhang C. (2021). LNG-IUS combined with progesterone ameliorates endometrial thickness and pregnancy outcomes of patients with early-stage endometrial cancer or atypical hyperplasia. Am. J. Transl. Res..

[20] Minig L., Franchi D., Boveri S., Casadio C., Bocciolone L., Sideri M. (2011). Progestin intrauterine device and GnRH analogue for uterus-sparing treatment of endometrial precancers and well-differentiated early endometrial carcinoma in young women. Ann. Oncol..

[21] Pashov A.I., Tskhay V.B., Ionouchene S.V. (2012). The combined GnRH-agonist and intrauterine levonorgestrel-releasing system treatment of complicated atypical hyperplasia and endometrial cancer: A pilot study. Gynecol. Endocrinol..

[22] Niwa K., Tagami K., Lian Z., Onogi K., Mori H., Tamaya T. (2005). Outcome of fertility-preserving treatment in young women with endometrial carcinomas. BJOG.

[23] Kim M.K., Seong S.J., Kang S.B., Bae D.S., Kim J.W., Nam J.H., Lim M.C., Lee T.S., Kim S., Paek J. (2019). Six months response rate of combined oral medroxyprogesterone/levonorgestrel-intrauterine system for early-stage endometrial cancer in young women: A Korean Gynecologic-Oncology Group Study. J. Gynecol. Oncol..

[24] Laurelli G., Falcone F., Gallo M.S., Scala F., Losito S., Granata V., Cascella M., Greggi S. (2016). Long-Term Oncologic and Reproductive Outcomes in Young Women with Early Endometrial Cancer Conservatively Treated: A Prospective Study and Literature Update. Int. J. Gynecol. Cancer.

[25] Eftekhar Z., Izadi-Mood N., Yarandi F., Shojaei H., Rezaei Z., Mohagheghi S. (2009). Efficacy of megestrol acetate (megace) in the treatment of patients with early endometrial adenocarcinoma: Our experiences with 21 patients. Int. J. Gynecol. Cancer.

[26] Shirali E., Yarandi F., Eftekhar Z., Shojaei H., Khazaeipour Z. (2012). Pregnancy outcome in patients with stage 1a endometrial adenocarcinoma, who conservatively treated with megestrol acetate. Arch. Gynecol. Obstet..

[27] Falcone F., Laurelli G., Losito S., Di Napoli M., Granata V., Greggi S. (2017). Fertility preserving treatment with hysteroscopic resection followed by progestin therapy in young women with early endometrial cancer. J. Gynecol. Oncol..

[28] Kim M.K., Seong S.J., Kim Y.S., Song T., Kim M.L., Yoon B.S., Jun H.S., Lee Y.H. (2013). Combined medroxyprogesterone acetate/levonorgestrel-intrauterine system treatment in young women with early-stage endometrial cancer. Am. J. Obstet. Gynecol..

[29] Chen J., Cao D., Yang J., Yu M., Zhou H., Cheng N., Wang J., Zhang Y., Peng P., Shen K. (2022). Fertility-Sparing Treatment for Endometrial Cancer or Atypical Endometrial Hyperplasia Patients with Obesity. Front. Oncol..

[30] Ouasti S., Bucau M., Larouzee E., Clement De Givry S., Chabbert-Buffet N., Koskas M. (2022). Prospective study of fertility-sparing treatment with chlormadinone acetate for endometrial carcinoma and atypical hyperplasia in young women. Int. J. Gynaecol. Obstet..

[31] Ushijima K., Yahata H., Yoshikawa H., Konishi I., Yasugi T., Saito T., Nakanishi T., Sasaki H., Saji F., Iwasaka T. (2007). Multicenter phase II study of fertility-sparing treatment with medroxyprogesterone acetate for endometrial carcinoma and atypical hyperplasia in young women. J. Clin. Oncol..

[32] Pronin S.M., Novikova O.V., Andreeva J.Y., Novikova E.G. (2015). Fertility-Sparing Treatment of Early Endometrial Cancer and Complex Atypical Hyperplasia in Young Women of Childbearing Potential. Int. J. Gynecol. Cancer.

[33] Novikova O.V., Nosov V.B., Panov V.A., Novikova E.G., Krasnopolskaya K.V., Andreeva Y.Y., Shevchuk A.S. (2021). Live births and maintenance with levonorgestrel IUD improve disease-free survival after fertility-sparing treatment of atypical hyperplasia and early endometrial cancer. Gynecol. Oncol..

[34] Bokhman J.V., Chepick O.F., Volkova A.T., Vishnevsky A.S. (1985). Can primary endometrial carcinoma stage I be cured without surgery and radiation therapy?. Gynecol. Oncol..

[35] Jafari Shobeiri M., Mostafa Gharabaghi P., Esmaeili H., Ouladsahebmadarek E., Mehrzad-Sadagiani M. (2013). Fertility sparing treatment in young patients with early endometrial adenocarcinoma: Case series. Pak. J. Med. Sci..

[36] Yuan F., Hu Y., Han X., Li Q. (2022). Metformin in Combination with Progesterone Improves the Pregnancy Rate for Patients with Early Endometrial Cancer. Contrast Media Mol. Imaging.

[37] Yang H.C., Liu J.C., Liu F.S. (2019). Fertility-preserving treatment of stage IA, well-differentiated endometrial carcinoma in young women with hysteroscopic resection and high-dose progesterone therapy. Taiwan J. Obstet. Gynecol..

[38] Yang Y.C., Wu C.C., Chen C.P., Chang C.L., Wang K.L. (2005). Reevaluating the safety of fertility-sparing hormonal therapy for early endometrial cancer. Gynecol. Oncol..

[39] Wang C.B., Wang C.J., Huang H.J., Hsueh S., Chou H.H., Soong Y.K., Lai C.H. (2002). Fertility-preserving treatment in young patients with endometrial adenocarcinoma. Cancer.

[40] Mazzon I., Corrado G., Masciullo V., Morricone D., Ferrandina G., Scambia G. (2010). Conservative surgical management of stage IA endometrial carcinoma for fertility preservation. Fertil. Steril..

[41] Wang Q., Guo Q., Gao S., Xie F., Du M., Dong J., Sui L., Xie K. (2015). Fertility-conservation combined therapy with hysteroscopic resection and oral progesterone for local early stage endometrial carcinoma in young women. Int. J. Clin. Exp. Med..

[42] Rossetti D., Bogani G., Carnelli M., Vitale S.G., Grosso G., Frigerio L. (2014). Efficacy of IVF following conservative management of endometrial cancer. Gynecol. Endocrinol..

[43] Yamazawa K., Hirai M., Fujito A., Nishi H., Terauchi F., Ishikura H., Shozu M., Isaka K. (2007). Fertility-preserving treatment with progestin, and pathological criteria to predict responses, in young women with endometrial cancer. Hum. Reprod..

[44] Signorelli M., Caspani G., Bonazzi C., Chiappa V., Perego P., Mangioni C. (2009). Fertility-sparing treatment in young women with endometrial cancer or atypical complex hyperplasia: A prospective single-institution experience of 21 cases. BJOG.

[45] Kim M.K., Yoon B.S., Park H., Seong S.J., Chung H.H., Kim J.W., Kang S.B. (2011). Conservative treatment with medroxyprogesterone acetate plus levonorgestrel intrauterine system for early-stage endometrial cancer in young women: Pilot study. Int. J. Gynecol. Cancer.

[46] Ayhan A., Tohma Y.A., Tunc M. (2020). Fertility preservation in early-stage endometrial cancer and endometrial intraepithelial neoplasia: A single-center experience. Taiwan J. Obstet. Gynecol..

[47] Kaku T., Yoshikawa H., Tsuda H., Sakamoto A., Fukunaga M., Kuwabara Y., Hataeg M., Kodama S., Kuzuya K., Sato S. (2001). Conservative therapy for adenocarcinoma and atypical endometrial hyperplasia of the endometrium in young women: Central pathologic review and treatment outcome. Cancer Lett..

[48] Andress J., Pasternak J., Walter C., Kommoss S., Kramer B., Hartkopf A., Brucker S.Y., Schonfisch B., Steinmacher S. (2021). Fertility preserving management of early endometrial cancer in a patient cohort at the department of women’s health at the university of Tuebingen. Arch. Gynecol. Obstet..

[49] Ohyagi-Hara C., Sawada K., Aki I., Mabuchi S., Kobayashi E., Ueda Y., Yoshino K., Fujita M., Tsutsui T., Kimura T. (2015). Efficacies and pregnant outcomes of fertility-sparing treatment with medroxyprogesterone acetate for endometrioid adenocarcinoma and complex atypical hyperplasia: Our experience and a review of the literature. Arch. Gynecol. Obstet..

[50] Casadio P., La Rosa M., Alletto A., Magnarelli G., Arena A., Fontana E., Fabbri M., Giovannico K., Virgilio A., Raimondo D. (2020). Fertility Sparing Treatment of Endometrial Cancer with and without Initial Infiltration of Myometrium: A Single Center Experience. Cancers.

[51] Giampaolino P., Di Spiezio Sardo A., Mollo A., Raffone A., Travaglino A., Boccellino A., Zizolfi B., Insabato L., Zullo F., De Placido G. (2019). Hysteroscopic Endometrial Focal Resection followed by Levonorgestrel Intrauterine Device Insertion as a Fertility-Sparing Treatment of Atypical Endometrial Hyperplasia and Early Endometrial Cancer: A Retrospective Study. J. Minim. Invasive Gynecol..

[52] Tamauchi S., Nakagawa A., Yoshida K., Yoshihara M., Yokoi A., Yoshikawa N., Niimi K., Kajiyama H. (2024). Update on the oncologic and obstetric outcomes of medroxyprogesterone acetate treatment for atypical endometrial hyperplasia and endometrial cancer. J. Obstet. Gynaecol. Res..

[53] Maggiore U.L.R., Martinelli F., Dondi G., Bogani G., Chiappa V., Evangelista M.T., Liberale V., Ditto A., Ferrero S., Raspagliesi F. (2019). Efficacy and fertility outcomes of levonorgestrel-releasing intra-uterine system treatment for patients with atypical complex hyperplasia or endometrial cancer: A retrospective study. J. Gynecol. Oncol..

[54] Baek J.S., Lee W.H., Kang W.D., Kim S.M. (2016). Fertility-preserving treatment in complex atypical hyperplasia and early endometrial cancer in young women with oral progestin: Is it effective?. Obstet. Gynecol. Sci..

[55] Roh H.J., Yoon H.J., Jeong D.H., Lee T.H., Kwon B.S., Suh D.S., Kim K.H. (2021). Prognostic factors of oncologic outcomes after fertility-preservative management with progestin in early-stage of endometrial cancer. J. Res. Med. Sci..

[56] Falcone F., Leone Roberti Maggiore U., Di Donato V., Perrone A.M., Frigerio L., Bifulco G., Polterauer S., Casadio P., Cormio G., Masciullo V. (2020). Fertility-sparing treatment for intramucous, moderately differentiated, endometrioid endometrial cancer: A Gynecologic Cancer Inter-Group (GCIG) study. J. Gynecol. Oncol..

[57] Gotlieb W.H., Beiner M.E., Shalmon B., Korach Y., Segal Y., Zmira N., Koupolovic J., Ben-Baruch G. (2003). Outcome of fertility-sparing treatment with progestins in young patients with endometrial cancer. Obstet. Gynecol..

[58] Kuang J., Sun S., Xu H., Ni R., Ke F. (2021). Curative effects of hysteroscopic resection combined with progesterone on early-stage endometrial cancer and its prognosis. J. BUON.

[59] Chae S.H., Shim S.H., Lee S.J., Lee J.Y., Kim S.N., Kang S.B. (2019). Pregnancy and oncologic outcomes after fertility-sparing management for early stage endometrioid endometrial cancer. Int. J. Gynecol. Cancer.

[60] Wang F., Yu A., Xu H., Zhang X., Li L., Lou H., Yu H., Lin J. (2017). Fertility Preserved Hysteroscopic Approach for the Treatment of Stage Ia Endometrioid Carcinoma. Int. J. Gynecol. Cancer.

[61] Park J.Y., Kim D.Y., Kim J.H., Kim Y.M., Kim K.R., Kim Y.T., Seong S.J., Kim T.J., Kim J.W., Kim S.M. (2013). Long-term oncologic outcomes after fertility-sparing management using oral progestin for young women with endometrial cancer (KGOG 2002). Eur. J. Cancer.

[62] Park H., Seok J.M., Yoon B.S., Seong S.J., Kim J.Y., Shim J.Y., Park C.T. (2012). Effectiveness of high-dose progestin and long-term outcomes in young women with early-stage, well-differentiated endometrioid adenocarcinoma of uterine endometrium. Arch. Gynecol. Obstet..

[63] Hahn H.S., Yoon S.G., Hong J.S., Hong S.R., Park S.J., Lim J.Y., Kwon Y.S., Lee I.H., Lim K.T., Lee K.H. (2009). Conservative treatment with progestin and pregnancy outcomes in endometrial cancer. Int. J. Gynecol. Cancer.

[64] Jing C.Y., Li S.N., Shan B.E., Zhang W., Tian W.J., Ren Y.L., Wang H.Y. (2022). Hysteroscopic Curettage Followed by Megestrol Acetate Plus Metformin as a Fertility-Sparing Treatment for Women with Atypical Endometrial Hyperplasia or Well-Differentiated Endometrioid Endometrial Carcinoma. Clin. Med. Insights Oncol..

[65] Ou Y.C., Fu H.C., Lan J., Wu C.H., Kung F.T., Lan K.C., Tsai Y.C., Lin H. (2021). The role of prolonged progestin treatment and factors predicting successful fertility-sparing treatment for early endometrial endometrioid adenocarcinoma. Eur. J. Obstet. Gynecol. Reprod. Biol..

[66] Park J.Y., Kim D.Y., Kim T.J., Kim J.W., Kim J.H., Kim Y.M., Kim Y.T., Bae D.S., Nam J.H. (2013). Hormonal therapy for women with stage IA endometrial cancer of all grades. Obstet. Gynecol..

[67] Wang C.J., Chao A., Yang L.Y., Hsueh S., Huang Y.T., Chou H.H., Chang T.C., Lai C.H. (2014). Fertility-preserving treatment in young women with endometrial adenocarcinoma: A long-term cohort study. Int. J. Gynecol. Cancer.

[68] Chen M., Jin Y., Li Y., Bi Y., Shan Y., Pan L. (2016). Oncologic and reproductive outcomes after fertility-sparing management with oral progestin for women with complex endometrial hyperplasia and endometrial cancer. Int. J. Gynaecol. Obstet..

[69] Mitsuhashi A., Habu Y., Kobayashi T., Kawarai Y., Ishikawa H., Usui H., Shozu M. (2019). Long-term outcomes of progestin plus metformin as a fertility-sparing treatment for atypical endometrial hyperplasia and endometrial cancer patients. J. Gynecol. Oncol..

[70] Koskas M., Azria E., Walker F., Luton D., Madelenat P., Yazbeck C. (2012). Progestin treatment of atypical hyperplasia and well-differentiated adenocarcinoma of the endometrium to preserve fertility. Anticancer Res..

[71] Cade T.J., Quinn M.A., Rome R.M., Neesham D. (2010). Progestogen treatment options for early endometrial cancer. BJOG.

[72] Catena U., Della Corte L., Raffone A., Travaglino A., Lucci Cordisco E., Teodorico E., Masciullo V., Bifulco G., Di Spiezio Sardo A., Scambia G. (2022). Fertility-sparing treatment for endometrial cancer and atypical endometrial hyperplasia in patients with Lynch Syndrome: Molecular diagnosis after immunohistochemistry of MMR proteins. Front. Med..

[73] Duska L.R., Garrett A., Rueda B.R., Haas J., Chang Y., Fuller A.F. (2001). Endometrial cancer in women 40 years old or younger. Gynecol. Oncol..

[74] Fujiwara H., Jobo T., Takei Y., Saga Y., Imai M., Arai T., Taneichi A., Machida S., Takahashi Y., Suzuki M. (2012). Fertility-sparing treatment using medroxyprogesterone acetate for endometrial carcinoma. Oncol. Lett..

[75] Fukui Y., Taguchi A., Adachi K., Sato M., Kawata A., Tanikawa M., Sone K., Mori M., Nagasaka K., Matsumoto Y. (2017). Polycystic Ovarian Morphology may be a Positive Prognostic Factor in Patients with Endometrial Cancer who Achieved Complete Remission after Fertility-Sparing Therapy with Progestin. Asian Pac. J. Cancer Prev..

[76] Hubbs J.L., Saig R.M., Abaid L.N., Bae-Jump V.L., Gehrig P.A. (2013). Systemic and local hormone therapy for endometrial hyperplasia and early adenocarcinoma. Obstet. Gynecol..

[77] Hwang J.Y., Kim D.H., Bae H.S., Kim M.L., Jung Y.W., Yun B.S., Seong S.J., Shin E., Kim M.K. (2017). Combined Oral Medroxyprogesterone/Levonorgestrel-Intrauterine System Treatment for Women with Grade 2 Stage IA Endometrial Cancer. Int. J. Gynecol. Cancer.

[78] Mao Y., Wan X., Chen Y., Lv W., Xie X. (2010). Outcomes of conservative therapy for young women with early endometrial adenocarcinoma. Fertil. Steril..

[79] Mentrikoski M.J., Shah A.A., Hanley K.Z., Atkins K.A. (2012). Assessing endometrial hyperplasia and carcinoma treated with progestin therapy. Am. J. Clin. Pathol..

[80] Minaguchi T., Nakagawa S., Takazawa Y., Nei T., Horie K., Fujiwara T., Osuga Y., Yasugi T., Kugu K., Yano T. (2007). Combined phospho-Akt and PTEN expressions associated with post-treatment hysterectomy after conservative progestin therapy in complex atypical hyperplasia and stage Ia, G1 adenocarcinoma of the endometrium. Cancer Lett..

[81] Ota T., Yoshida M., Kimura M., Kinoshita K. (2005). Clinicopathologic study of uterine endometrial carcinoma in young women aged 40 years and younger. Int. J. Gynecol. Cancer.

[82] Ushijima K., Tsuda N., Yamagami W., Mitsuhashi A., Mikami M., Yaegashi N., Enomoto T. (2023). Trends and characteristics of fertility-sparing treatment for atypical endometrial hyperplasia and endometrial cancer in Japan: A survey by the Gynecologic Oncology Committee of the Japan Society of Obstetrics and Gynecology. J. Gynecol. Oncol..

[83] Wheeler D.T., Bristow R.E., Kurman R.J. (2007). Histologic alterations in endometrial hyperplasia and well-differentiated carcinoma treated with progestins. Am. J. Surg. Pathol..

[84] Pino I., Iacobone A.D., Vidal Urbinati A.M., Di Giminiani M., Radice D., Guerrieri M.E., Preti E.P., Martella S., Franchi D. (2022). Fertility-Sparing Treatment for Endometrial Cancer: Oncological and Obstetric Outcomes in Combined Therapies with Levonorgestrel Intrauterine Device. Cancers.

[85] Perri T., Korach J., Gotlieb W.H., Beiner M., Meirow D., Friedman E., Ferenczy A., Ben-Baruch G. (2011). Prolonged conservative treatment of endometrial cancer patients: More than 1 pregnancy can be achieved. Int. J. Gynecol. Cancer.

[86] Yahata T., Fujita K., Aoki Y., Tanaka K. (2006). Long-term conservative therapy for endometrial adenocarcinoma in young women. Hum. Reprod..

[87] Parlakgumus H.A., Kilicdag E.B., Simsek E., Haydardedeoglu B., Cok T., Aytac P.C., Bagis T., Erkanli S. (2014). Fertility outcomes of patients with early stage endometrial carcinoma. J. Obstet. Gynaecol. Res..

[88] Kim Y.B., Holschneider C.H., Ghosh K., Nieberg R.K., Montz F.J. (1997). Progestin alone as primary treatment of endometrial carcinoma in premenopausal women. Report of seven cases and review of the literature. Cancer.

[89] Dagher C., Manning-Geist B., Ellenson L.H., Weigelt B., Rios-Doria E., Barry D., Abu-Rustum N.R., Leitao M.M., Mueller J.J. (2023). Molecular subtyping in endometrial cancer: A promising strategy to guide fertility preservation. Gynecol. Oncol..

[90] Pal N., Broaddus R.R., Urbauer D.L., Balakrishnan N., Milbourne A., Schmeler K.M., Meyer L.A., Soliman P.T., Lu K.H., Ramirez P.T. (2018). Treatment of Low-Risk Endometrial Cancer and Complex Atypical Hyperplasia with the Levonorgestrel-Releasing Intrauterine Device. Obstet. Gynecol..

[91] Yamagami W., Susumu N., Makabe T., Sakai K., Nomura H., Kataoka F., Hirasawa A., Banno K., Aoki D. (2018). Is repeated high-dose medroxyprogesterone acetate (MPA) therapy permissible for patients with early stage endometrial cancer or atypical endometrial hyperplasia who desire preserving fertility?. J. Gynecol. Oncol..

[92] De Rocco S., Buca D., Oronzii L., Petrillo M., Fanfani F., Nappi L., Liberati M., D’Antonio F., Scambia G., Leombroni M. (2022). Reproductive and pregnancy outcomes of fertility-sparing treatments for early-stage endometrial cancer or atypical hyperplasia: A systematic review and meta-analysis. Eur. J. Obstet. Gynecol. Reprod. Biol..

[93] Wei J., Zhang W., Feng L., Gao W. (2017). Comparison of fertility-sparing treatments in patients with early endometrial cancer and atypical complex hyperplasia: A meta-analysis and systematic review. Medicine.

[94] Zhao S., Zhang J., Yan Y., Tian L., Chen L., Zheng X., Sun Y., Tian W., Xue F., Wang Y. (2024). Oncological and reproductive outcomes of endometrial atypical hyperplasia and endometrial cancer patients undergoing conservative therapy with hysteroscopic resection: A systematic review and meta-analysis. Acta Obstet. Gynecol. Scand..

[95] Sebok P., Keszthelyi M., Vida B., Loczi L., Sebok B., Merkely P., Acs N., Keszthelyi A., Varbiro S., Lintner B. (2025). Oncologic and Reproductive Outcomes After Fertility-Sparing Treatments for Endometrial Hyperplasia with Atypia: A Systematic Review and Meta-Analysis. Cancers.

[96] Ogunbiyi M.O., Oxley S., Graham R., Olaitan A. (2024). The oncological and reproductive outcomes of fertility-preserving treatments for stage 1 grade 1 endometrial carcinoma: A systematic review and meta-analysis. J. Obstet. Gynaecol..

[97] Suzuki Y., Ferris J.S., Chen L., Dioun S., Usseglio J., Matsuo K., Xu X., Hershman D.L., Wright J.D. (2024). Fertility-preserving treatment for stage IA endometrial cancer: A systematic review and meta-analysis. Am. J. Obstet. Gynecol..

[98] Fernandez-Montoli M.E., Sabadell J., Contreras Perez N.A., Verdaguer Menendez-Arango P., Julia Torres C., Lleberia J. (2025). Fertility-sparing treatment for atypical endometrial hyperplasia and endometrial cancer. Cochrane Database Syst. Rev..

[99] Yang B., Xu Y., Zhu Q., Xie L., Shan W., Ning C., Xie B., Shi Y., Luo X., Zhang H. (2019). Treatment efficiency of comprehensive hysteroscopic evaluation and lesion resection combined with progestin therapy in young women with endometrial atypical hyperplasia and endometrial cancer. Gynecol. Oncol..

[100] De Marzi P., Bergamini A., Luchini S., Petrone M., Taccagni G.L., Mangili G., Colombo G., Candiani M. (2015). Hysteroscopic resection in fertility-sparing surgery for atypical hyperplasia and endometrial cancer: Safety and efficacy. J. Minim. Invasive Gynecol..

[101] Chen J., Cao D., Yang J., Yu M., Zhou H., Cheng N., Wang J., Zhang Y., Peng P., Shen K. (2022). Oncological and reproductive outcomes for gonadotropin-releasing hormone agonist combined with aromatase inhibitors or levonorgestrel-releasing intrauterine system in women with endometrial cancer or atypical endometrial hyperplasia. Int. J. Gynecol. Cancer.

[102] Tamauchi S., Kajiyama H., Utsumi F., Suzuki S., Niimi K., Sakata J., Mizuno M., Shibata K., Kikkawa F. (2018). Efficacy of medroxyprogesterone acetate treatment and retreatment for atypical endometrial hyperplasia and endometrial cancer. J. Obstet. Gynaecol. Res..

[103] Atallah D., El Kassis N., Safi J., El Hachem H., Chahine G., Moubarak M. (2021). The use of hysteroscopic endometrectomy in the conservative treatment of early endometrial cancer and atypical hyperplasia in fertile women. Arch. Gynecol. Obstet..

[104] Baker W.D., Pierce S.R., Mills A.M., Gehrig P.A., Duska L.R. (2017). Nonoperative management of atypical endometrial hyperplasia and grade 1 endometrial cancer with the levonorgestrel intrauterine device in medically ill postmenopausal women. Gynecol. Oncol..

[105] Fan Y., Li X., Wang J., Wang Y., Tian L., Wang J. (2021). Analysis of pregnancy-associated factors after fertility-sparing therapy in young women with early-stage endometrial cancer or atypical endometrial hyperplasia. Reprod. Biol. Endocrinol..

[106] He Y., Wang Y., Zhou R., Wang J. (2020). Oncologic and obstetrical outcomes after fertility-preserving retreatment in patients with recurrent atypical endometrial hyperplasia and endometrial cancer. Int. J. Gynecol. Cancer.

[107] Jadoul P., Donnez J. (2003). Conservative treatment may be beneficial for young women with atypical endometrial hyperplasia or endometrial adenocarcinoma. Fertil. Steril..

[108] Janda M., Robledo K.P., Gebski V., Armes J.E., Alizart M., Brennan D., Cummings M., Chen C., Leung Y., Sykes P. (2021). Complete pathological response following levonorgestrel intrauterine device in clinically stage I endometrial adenocarcinoma: Results of a randomized clinical trial. Gynecol. Oncol..

[109] Le Digabel J.F., Gariel C., Catala L., Dhainaut C., Madelenat P., Descamps P. (2006). Young women with atypical endometrial hyperplasia or endometrial adenocarcinoma stage I: Will conservative treatment allow pregnancy? Results of a French multicentric survey. Gynecol. Obstet. Fertil..

[110] Marnach M.L., Butler K.A., Henry M.R., Hutz C.E., Langstraat C.L., Lohse C.M., Casey P.M. (2017). Oral progestogens versus levonorgestrel-releasing intrauterine system for treatment of endometrial intraepithelial neoplasia. J. Womens Health.

[111] Masciullo V., Trivellizzi N., Zannoni G., Catena U., Moroni R., Fanfani F., Scambia G. (2021). Prognostic impact of hysteroscopic resection of endometrial atypical hyperplasia–endometrioid intraepithelial neoplasia and early-stage cancer in combination with megestrol acetate. Am. J. Obstet. Gynecol..

[112] Tock S., Jadoul P., Squifflet J.L., Marbaix E., Baurain J.F., Luyckx M. (2018). Fertility-sparing treatment in patients with early-stage endometrial cancer using a combination of surgery and GnRH agonist: A monocentric retrospective study and review of the literature. Front. Med..

[113] van Gent M.D., Nicolae-Cristea A.R., de Kroon C.D., Osse E.M., Kagie M.J., Trimbos J.B., Hazelbag H.M., Smit V.T., Bosse T. (2016). Exploring morphologic and molecular aspects of endometrial cancer under progesterone treatment in the context of fertility preservation. Int. J. Gynecol. Cancer.

[114] Wang Y.Q., Kang N., Li L.W., Wang Z.Q., Zhou R., Shen D.H., Wang J.L. (2022). Significance of molecular classification in fertility-sparing treatment of endometrial carcinoma and atypical endometrial hyperplasia. Zhonghua Fu Chan Ke Za Zhi.

[115] Yang Y.F., Liao Y.Y., Liu X.L., Su S.G., Li L.Z., Peng N.F. (2015). Prognostic factors of regression and relapse of complex atypical hyperplasia and well-differentiated endometrioid carcinoma with conservative treatment. Gynecol. Oncol..

[116] Varma R., Soneja H., Bhatia K., Ganesan R., Rollason T., Clark T.J., Gupta J.K. (2008). The effectiveness of a levonorgestrel-releasing intrauterine system (LNG-IUS) in the treatment of endometrial hyperplasia: A long-term follow-up study. Eur. J. Obstet. Gynecol. Reprod. Biol..

[117] Ørbo A., Arnes M., Lyså L.M., Borgfeldt C., Straume B. (2016). HE4 is a novel tissue marker for therapy response and progestin resistance in medium- and low-risk endometrial hyperplasia. Br. J. Cancer.

[118] von Minckwitz G., Loibl S., Brunnert K., Kreienberg R., Melchert F., Mösch R., Neises M., Schermann J., Seufert R., Stiglmayer R. (2002). Adjuvant endocrine treatment with medroxyprogesterone acetate or tamoxifen in stage I and II endometrial cancer: A multicentre, open, controlled, prospectively randomised trial. Eur. J. Cancer.

[119] Sletten E.T., Arnes M., Lyså L.M., Larsen M., Ørbo A. (2019). Significance of progesterone receptors (PR-A and PR-B) expression as predictors for relapse after successful therapy of endometrial hyperplasia: A retrospective cohort study. BJOG.

[120] Wu P., Lv Q., Guan J., Shan W., Chen X., Zhu Q., Luo X. (2022). Clinical implications of morular metaplasia in fertility-preserving treatment for atypical endometrial hyperplasia and early endometrial carcinoma patients. Arch. Gynecol. Obstet..

[121] Yoshimura T., Yamagami W., Takahashi M., Hirano T., Sakai K., Makabe T., Chiyoda T., Banno K., Aoki D. (2022). Clinical usefulness of endometrial cytology in determining the therapeutic effect of fertility-preserving therapy. Acta Cytol..

[122] Liu J., Wang S., Li S., Liu X. (2022). Efficacy of levonorgestrel-intrauterine releasing system combined with goserelin in treatment of atypical endometrial hyperplasia. Lat. Am. J. Pharm..

[123] He Y., Wang J., Wang Y., Zhou R., Lu Q., Liu G., Tang H., Guo H., He M., Wu G. (2021). Maintenance therapy can improve the oncologic prognosis and obstetrical outcome of patients with atypical endometrial hyperplasia and endometrial cancer after fertility-preserving treatment: A multicenter retrospective study. Front. Oncol..

[124] Wang Y., Zhou R., Zhang X., Liu H., Shen D., Wang J. (2021). Significance of serum and pathological biomarkers in fertility-sparing treatment for endometrial cancer or atypical hyperplasia: A retrospective cohort study. BMC Womens Health.

[125] Li X., Fan Y., Wang J., Zhou R., Tian L., Wang Y., Wang J. (2021). Insulin resistance and metabolic syndrome increase the risk of relapse for fertility-preserving treatment in atypical endometrial hyperplasia and early endometrial cancer patients. Front. Oncol..

[126] Shan Y., Qin M., Yin J., Cai Y., Li Y., Gu Y., Wang W., Wang Y.X., Chen J.Y., Jin Y. (2021). Effect and management of excess weight in the context of fertility-sparing treatments in patients with atypical endometrial hyperplasia and endometrial cancer: Eight-year experience of 227 cases. Front. Oncol..

[127] Wang L., Luo X., Wang Q., Lv Q., Wu P., Liu W., Chen X. (2021). Fertility-preserving treatment outcome in endometrial cancer or atypical hyperplasia patients with polycystic ovary syndrome. J. Gynecol. Oncol..

[128] Piatek S., Michalski W., Sobiczewski P., Bidzinski M., Szewczyk G. (2021). The results of different fertility-sparing treatment modalities and obstetric outcomes in patients with early endometrial cancer and atypical endometrial hyperplasia: Case series of 30 patients and systematic review. Eur. J. Obstet. Gynecol. Reprod. Biol..

[129] Sengal A.T., Smith D., Rogers R., Snell C.E., Williams E.D., Pollock P.M. (2021). Fibroblast growth factor receptor 2 isoforms detected via novel RNA ISH as predictive biomarkers for progestin therapy in atypical hyperplasia and low-grade endometrial cancer. Cancers.

[130] Westin S.N., Fellman B., Sun C.C., Broaddus R.R., Woodall M.L., Pal N., Urbauer D.L., Ramondetta L.M., Schmeler K.M., Soliman P.T. (2021). Prospective phase II trial of levonorgestrel intrauterine device: Nonsurgical approach for complex atypical hyperplasia and early-stage endometrial cancer. Am. J. Obstet. Gynecol..

[131] Matsuo K., Mandelbaum R.S., Ciccone M., Khoshchehreh M., Pursuwani H., Morocco E.B., Matsuzaki S., Dancz C.E., Ozel B., Paulson R.J. (2020). Route-specific association of progestin therapy and concurrent metformin use in obese women with complex atypical hyperplasia. Int. J. Gynecol. Cancer.

[132] Kim M.K., Seong S.J. (2020). Response to comment on: Comparison of diagnostic accuracy between endometrial curettage and aspiration biopsy in patients treated with progestin for endometrial hyperplasia: A Korean gynecologic oncology group study. J. Gynecol. Oncol..

[133] Mandelbaum R.S., Ciccone M.A., Nusbaum D.J., Khoshchehreh M., Purswani H., Morocco E.B., Smith M.B., Matsuzaki S., Dancz C.E., Ozel B. (2020). Progestin therapy for obese women with complex atypical hyperplasia: Levonorgestrel-releasing intrauterine device versus systemic therapy. Am. J. Obstet. Gynecol..

[134] Behrouzi R., Ryan N.A.J., Barr C.E., Derbyshire A.E., Wan Y.L., Maskell Z., Stocking K., Pemberton P.W., Bolton J., McVey R.J. (2020). Baseline serum HE4 but not tissue HE4 expression predicts response to the levonorgestrel-releasing intrauterine system in atypical hyperplasia and early-stage endometrial cancer. Cancers.

[135] Wang Y., Zhou R., Wang H., Liu H., Wang J. (2019). Impact of treatment duration in fertility-preserving management of endometrial cancer or atypical endometrial hyperplasia. Int. J. Gynecol. Cancer.

[136] Kim S.R., van der Zanden C., Ikiz H., Kuzelijevic B., Havelock J., Kwon J.S. (2018). Fertility-Sparing Management Using Progestin for Young Women with Endometrial Cancer From a Population-Based Study. J. Obstet. Gynaecol. Can..

[137] Zhou H., Cao D., Yang J., Shen K., Lang J. (2017). Gonadotropin-releasing hormone agonist combined with a levonorgestrel-releasing intrauterine system or letrozole for fertility-preserving treatment of endometrial carcinoma and complex atypical hyperplasia in young women. Int. J. Gynecol. Cancer.

[138] Zhang H., Yan L., Bai Y., Li C., Guo Q., Wang C., Zhao X., Li M. (2015). Dual-specificity phosphatase 6 predicts the sensitivity of progestin therapy for atypical endometrial hyperplasia. Gynecol. Oncol..

[139] Gonthier C., Walker F., Luton D., Yazbeck C., Madelenat P., Koskas M. (2014). Impact of obesity on the results of fertility-sparing management for atypical hyperplasia and grade 1 endometrial cancer. Gynecol. Oncol..

[140] Gunderson C.C., Dutta S., Fader A.N., Maniar K.P., Nasseri-Nik N., Bristow R.E., Diaz-Montes T.P., Palermo R., Kurman R.J. (2014). Pathologic features associated with resolution of complex atypical hyperplasia and grade 1 endometrial adenocarcinoma after progestin therapy. Gynecol. Oncol..

[141] Simpson A.N., Feigenberg T., Clarke B.A., Gien L.T., Ismiil N., Laframboise S., Massey C., Ferguson S.E. (2014). Fertility-sparing treatment of complex atypical hyperplasia and low-grade endometrial cancer using oral progestin. Gynecol. Oncol..

[142] Cade T.J., Quinn M.A., Rome R.M., Neesham D. (2013). Long-term outcomes after progestogen treatment for early endometrial cancer. Aust. N. Z. J. Obstet. Gynaecol..

[143] Goncharenko V.M., Beniuk V.A., Kalenska O.V., Demchenko O.M., Spivak M.Y., Bubnov R.V. (2013). Predictive diagnosis of endometrial hyperplasia and personalized therapeutic strategy in women of fertile age. EPMA J..

[144] Kim M.K., Seong S.J., Song T., Kim M.L., Yoon B.S., Jun H.S., Lee G.H., Lee Y.H. (2013). Comparison of dilatation and curettage and endometrial aspiration biopsy accuracy in patients treated with high-dose oral progestin plus levonorgestrel intrauterine system for early-stage endometrial cancer. Gynecol. Oncol..

[145] Gallos I.D., Devey J., Ganesan R., Gupta J.K. (2013). Predictive ability of estrogen receptor (ER), progesterone receptor (PR), COX-2, Mlh1, and Bcl-2 expressions for regression and relapse of endometrial hyperplasia treated with LNG-IUS: A prospective cohort study. Gynecol. Oncol..

[146] Bakkum-Gamez J.N., Kalogera E., Keeney G.L., Mariani A., Podratz K.C., Dowdy S.C. (2012). Conservative management of atypical hyperplasia and grade I endometrial carcinoma: Review of the literature and presentation of a series. J. Gynecol. Surg..

[147] Upson K., Allison K.H., Reed S.D., Jordan C.D., Newton K.M., Swisher E.M., Doherty J.A., Garcia R.L. (2012). Biomarkers of progestin therapy resistance and endometrial hyperplasia progression. Am. J. Obstet. Gynecol..

[148] Haoula Z.J., Walker K.F., Powell M.C. (2011). Levonorgestrel intrauterine system as a treatment option for complex endometrial hyperplasia. Eur. J. Obstet. Gynecol. Reprod. Biol..

[149] Ørbo A., Arnes M., Pettersen I., Larsen K., Hanssen K., Moe B. (2010). Down-regulated progesterone receptor A and B coinciding with successful treatment of endometrial hyperplasia by the levonorgestrel-impregnated intrauterine system. Acta Obstet. Gynecol. Scand..

[150] Vereide A.B., Kaino T., Sager G., Ørbo A., Scottish Gynaecological Clinical Trials Group (2005). Bcl-2, BAX, and apoptosis in endometrial hyperplasia after high-dose gestagen therapy: A comparison of responses in patients treated with intrauterine levonorgestrel and systemic medroxyprogesterone. Gynecol. Oncol..

[151] Montz F.J., Bristow R.E., Bovicelli A., Tomacruz R., Kurman R.J. (2002). Intrauterine progesterone treatment of early endometrial cancer. Am. J. Obstet. Gynecol..

[152] Lago V., Marina T., Laseca Modrego M., Gil-Ibañez B., Rodriguez J.R., Domingo J., Minig L., Padilla-Iserte P., Arencibia Sánchez O., Sala Ferichola M. (2022). Fertility-sparing treatment in patients with endometrial cancer (FERT-ENC): A multicentric retrospective study from the Spanish Investigational Network Gynecologic Oncology Group (SPAIN-GOG). Arch. Gynecol. Obstet..

[153] Chung Y.S., Woo H.Y., Lee J.Y., Park E., Nam E.J., Kim S., Kim S.W., Kim Y.T. (2021). Mismatch repair status influences response to fertility-sparing treatment of endometrial cancer. Am. J. Obstet. Gynecol..

[154] Kudesia R., Singer T., Caputo T.A., Holcomb K.M., Kligman I., Rosenwaks Z., Gupta D. (2014). Reproductive and oncologic outcomes after progestin therapy for endometrial complex atypical hyperplasia or carcinoma. Am. J. Obstet. Gynecol..

[155] Greenwald Z.R., Huang L.N., Wissing M.D., Franco E.L., Gotlieb W.H. (2017). Does hormonal therapy for fertility preservation affect the survival of young women with early-stage endometrial cancer?. Cancer.

[156] Yin J., Li Y., Wang H., Wang W., Gu Y., Jin Y., Deng C., Pan L. (2023). Clinical outcomes of levonorgestrel-releasing intrauterine device present during controlled ovarian stimulation in patients with early stage endometrioid adenocarcinoma and atypical endometrial hyperplasia after fertility-sparing treatments: 10-year experience in one tertiary hospital in China. Eur. J. Obstet. Gynecol. Reprod. Biol..

[157] Vereide A.B., Kaino T., Sager G., Arnes M., Ørbo A. (2006). Effect of levonorgestrel IUD and oral medroxyprogesterone acetate on glandular and stromal progesterone receptors (PRA and PRB), and estrogen receptors (ER-alpha and ER-beta) in human endometrial hyperplasia. Gynecol. Oncol..

[158] Wu P., Shan W., Xue Y., Wang L., Liu S., Chen X., Luo X. (2024). Impacts of ovarian reserve on conservative treatment for endometrial cancer and atypical hyperplasia. Front. Endocrinol..

[159] Barr C.E., Sergeant J.C., Agnew H.J., Bolton J., McVey R.J., Crosbie E.J. (2023). Serum HE4 predicts progestin treatment response in endometrial cancer and atypical hyperplasia: A prognostic study. BJOG.

[160] Chaudhari S.R., Lai T.S., Zakhour M., Shin S.M., Baltayan A., Tan H., Cohen J.G. (2023). Comparison of Mirena and Liletta levonorgestrel intrauterine devices for the treatment of endometrial intraepithelial neoplasia and grade 1 endometrioid endometrial cancer. Gynecol. Oncol. Rep..

[161] Xue Y., Dong Y., Lou Y., Lv Q., Shan W., Wang C., Chen X. (2023). PTEN mutation predicts unfavorable fertility preserving treatment outcome in the young patients with endometrioid endometrial cancer and atypical hyperplasia. J. Gynecol. Oncol..

[162] Xi Y., Liu G., Liu D., Jiang J., Gong R. (2023). Efficacy and pregnancy outcomes of hysteroscopic surgery combined with progestin as fertility-sparing therapy in patients with early-stage endometrial cancer and atypical hyperplasia. Arch. Gynecol. Obstet..

[163] Wang Y., Bo L., Fan X., Kang N., Zhang X., Tian L., Zhou R., Wang J. (2025). Molecular classification guides fertility-sparing treatment for endometrial cancer and atypical hyperplasia patients. Curr. Oncol..

[164] Ga H., Taguchi A., Honjoh H., Nishijima A., Eguchi S., Miyamoto Y., Sone K., Mori M., Osuga Y. (2023). Prognosis of patients with endometrial cancer or atypical endometrial hyperplasia after complete remission with fertility-sparing therapy. Arch. Gynecol. Obstet..

[165] Fu P., Sun H., Zhou T., Cui P., Wang S., Liu R. (2023). Postoperative adjuvant treatment in women with stage I endometrial cancer: A retrospective study. Int. J. Clin. Pract..

[166] Chung Y.S., Lee J.Y., Nam E.J., Kim S., Kim S.W., Kim Y.T. (2019). EP498 Oncologic and pregnancy outcomes with fertility-sparing management for early endometrial cancer in young women. Int. J. Gynecol. Cancer.

[167] Milishkevich A.G., Mavrichev S.A., Matylevich O.P., Dalamanava A.V., Shelkovich S.Y. (2022). 2022-RA-427-ESGO The results of fertility-sparing treatment and obstetric outcomes in patients with atypical endometrial hyperplasia andearly endometrial cancer: A case series from belarus. Int. J. Gynecol. Cancer.

[168] Lv X., Guo L., Wang C. (2024). Efficacy of fertility-sparing treatment with LNG-IUS is associated with different ProMisE subtypes of endometrial carcinoma or atypical endometrial hyperplasia. J. Gynecol. Oncol..

[169] Lin L.H., Founta K., Chambwe N., DeLair D.F. (2024). DNA methylation profiling identifies a subset of low-grade endometrial neoplasms with poor response to progestin therapy. Clin. Cancer Res..

