# Supplementary material for: Oncologic and Reproductive Outcomes of Fertility-Sparing Management in Early-Stage Endometrial Carcinoma: A Systematic Review and Meta-Analysis
Source: Cancers (Basel). 2026 Jan 27;18(3):399. doi: 10.3390/cancers18030399 (PMC12897394; doi:10.3390/cancers18030399)
Supplement: Supplementary file 1 [file cancers-18-00399-s001.zip › Supplemental Table S1.pdf]

Supplementary Table S1. GRADE Summary of Findings tables for fertility-sparing treatments in early stage endometrial carcinoma. This table presents the certainty of evidence assessments for primary and secondary outcomes, including complete response, recurrence, pregnancy, live birth, partial response, and no response. For each intervention comparison, the table reports the number of studies, study design, risk of bias, inconsistency, indirectness, imprecision, and other considerations, alongside the total number of patients, relative effect estimates (with 95% confidence intervals), absolute risks, and final certainty rating. Critical and important outcomes are labeled according to their clinical relevance.

	Certainty assessment							Effect				
Intervention	№ of studies	Study design	Risk of bias	Inconsistency	Indirectness	Imprecision	Other considerations	No. of patients	Relative (95% CI)	Absolute (95% CI)	Certainty	Importance
**Complete Response rate**												
Oral progestin vs LNG-IUD	56	Randomised and nonrandomised studies	Not serious	Not serious	Not serious	Not serious	No additional considerations; the pooled effect estimate was consistent across studies with adequate precision.	1519	1.22 (0.88–1.79)	72% vs 59%	Moderate	Critical
Oral progestin vs Oral + LNG-IUD	57	Randomised and nonrandomised studies	Not serious	Serious	Not serious	Not serious	Downgraded due to unexplained heterogeneity across studies, with inconsistent effect estimates and overlapping confidence intervals.	1588	1.00 (0.76–1.38)	72% vs 72%	Low	Critical

Intervention	Certainty assessment							Effect			Certainty	Importance
	№ of studies	Study design	Risk of bias	Inconsistency	Indirectness	Imprecision	Other considerations	No. of patients	Relative (95% CI)	Absolute (95% CI)		
Oral + Hysteroscopy vs Oral progestin	60	Randomised and nonrandomised studies	Not serious	Serious	Not serious	Not serious	Downgraded due to unexplained heterogeneity across studies, with inconsistent effect estimates and overlapping confidence intervals.	1588	1.18 (0.87–1.51)	85% vs 72%	Low	Critical
LNG-IUD + Hysteroscopy vs Oral progestin	55	Randomised and nonrandomised studies	Not serious	Not serious	Not serious	Not serious	No additional considerations; the pooled effect estimate was consistent across studies with adequate precision.	1478	1.18 (0.95–1.45)	85% vs 72%	Moderate	Critical
LNG-IUD + GnRHa vs Oral progestin	56	Randomised and nonrandomised studies	Not serious	Serious	Not serious	Not serious	Downgraded due to unexplained heterogeneity across studies, with inconsistent effect estimates and overlapping confidence intervals.	1676	1.15 (0.90–1.45)	83% vs 72%	Low	Critical
Oral + Metformin vs Oral progestin	53	Randomised and nonrandomised studies	Not serious	Serious	Not serious	Not serious	Downgraded due to unexplained heterogeneity across studies, with inconsistent effect estimates and overlapping confidence intervals.	1628	1.11 (0.61–1.54)	80% vs 72%	Low	Critical

Intervention	Certainty assessment							Effect			Certainty	Importance
	№ of studies	Study design	Risk of bias	Inconsistency	Indirectness	Imprecision	Other considerations	No. of patients	Relative (95% CI)	Absolute (95% CI)		
Oral + LNG-IUD vs LNG-IUD	17	Randomised and nonrandomised studies	Not serious	Not serious	Not serious	Not serious	No additional considerations; the pooled effect estimate was consistent across studies with adequate precision.	289	1.22 (0.76–2.00)	72% vs 59%	Moderate	Critical
Oral + Hysteroscopy vs LNG-IUD	18	Randomised and nonrandomised studies	Not serious	Not serious	Not serious	Not serious	No additional considerations; the pooled effect estimate was consistent across studies with adequate precision.	289	1.44 (0.91–2.28)	85% vs 59%	Moderate	Critical
LNG-IUD + Hysteroscopy vs LNG-IUD	13	Randomised and nonrandomised studies	Not serious	Not serious	Not serious	Not serious	No additional considerations; the pooled effect estimate was consistent across studies with adequate precision.	179	1.44 (0.99–2.19)	85% vs 59%	Low	Critical
LNG-IUD + GnRHa vs LNG-IUD	16	Randomised and nonrandomised studies	Not serious	Not serious	Not serious	Not serious	No additional considerations; the pooled effect estimate was consistent across studies with adequate precision.	377	1.41 (0.93–2.19)	83% vs 59%	Moderate	Critical

Intervention	Certainty assessment							Effect			Certainty	Importance
	№ of studies	Study design	Risk of bias	Inconsistency	Indirectness	Imprecision	Other considerations	No. of patients	Relative (95% CI)	Absolute (95% CI)		
Oral + Metformin vs LNG-IUD	15	Randomised and nonrandomised studies	Not serious	Serious	Not serious	Not serious	Downgraded due to unexplained heterogeneity across studies, with inconsistent effect estimates and overlapping confidence intervals.	329	1.36 (0.64–2.33)	80% vs 59%	Low	Critical
Oral + Hysteroscopy vs Oral + LNG-IUD	14	Randomised and nonrandomised studies	Not serious	Serious	Not serious	Not serious	Downgraded due to unexplained heterogeneity across studies, with inconsistent effect estimates and overlapping confidence intervals.	358	1.18 (0.78–1.75)	85% vs 72%	Low	Critical
LNG-IUD + Hysteroscopy vs Oral + LNG-IUD	14	Randomised and nonrandomised studies	Not serious	Not serious	Not serious	Not serious	No additional considerations; the pooled effect estimate was consistent across studies with adequate precision.	248	1.18 (0.85–1.68)	85% vs 72%	Moderate	Critical
LNG-IUD + GnRHa vs Oral + LNG-IUD	15	Randomised and nonrandomised studies	Not serious	Serious	Not serious	Not serious	Downgraded due to unexplained heterogeneity across studies, with inconsistent effect estimates and overlapping confidence intervals.	446	1.15 (0.80–1.68)	83% vs 72%	Low	Critical

Intervention	Certainty assessment							Effect			Certainty	Importance
	№ of studies	Study design	Risk of bias	Inconsistency	Indirectness	Imprecision	Other considerations	No. of patients	Relative (95% CI)	Absolute (95% CI)		
Oral + Metformin vs Oral + LNG-IUD	15	Randomised and nonrandomised studies	Not serious	Serious	Not serious	Not serious	Downgraded due to unexplained heterogeneity across studies, with inconsistent effect estimates and overlapping confidence intervals.	398	1.11 (0.55–1.79)	80% vs 72%	Low	Critical
Oral + Hysteroscopy vs LNG-IUD + Hysteroscopy	11	Randomised and nonrandomised studies	Not serious	Not serious	Not serious	Not serious	Downgraded due to minimal differences between intervention effects, with relative effect estimates close to unity, limiting clinical interpretability.	248	1.00 (0.71–1.34)	85% vs 85%	Low	Critical
Oral + Hysteroscopy vs LNG-IUD + GnRHa	15	Randomised and nonrandomised studies	Not serious	Serious	Not serious	Not serious	Downgraded due to unexplained heterogeneity across studies, with inconsistent effect estimates and overlapping confidence intervals.	446	1.02 (0.71–1.42)	85% vs 83%	Low	Critical

Intervention	Certainty assessment							Effect			Certainty	Importance
	№ of studies	Study design	Risk of bias	Inconsistency	Indirectness	Imprecision	Other considerations	No. of patients	Relative (95% CI)	Absolute (95% CI)		
Oral + Hysteroscopy vs Oral + Metformin	14	Randomised and nonrandomised studies	Not serious	Serious	Not serious	Not serious	Downgraded due to unexplained heterogeneity across studies, with inconsistent effect estimates and overlapping confidence intervals.	398	1.06 (0.67–2.09)	85% vs 80%	Low	Critical
LNG-IUD + Hysteroscopy vs LNG-IUD + GnRHa	10	Randomised and nonrandomised studies	Not serious	Not serious	Not serious	Not serious	Downgraded due to minimal differences between intervention effects, with relative effect estimates close to unity, limiting clinical interpretability.	336	1.02 (0.78–1.36)	85% vs 83%	Low	Critical
LNG-IUD + Hysteroscopy vs Oral + Metformin	9	Randomised and nonrandomised studies	Not serious	Not serious	Not serious	Not serious	No additional considerations; the pooled effect estimate was consistent across studies with adequate precision.	288	1.06 (0.73–2.00)	85% vs 80%	Low	Critical
LNG-IUD + GnRHa vs Oral + Metformin	11	Randomised and nonrandomised studies	Not serious	Serious	Not serious	Not serious	Downgraded due to unexplained heterogeneity across studies, with inconsistent effect estimates and overlapping confidence intervals.	486	1.04 (0.69–2.00)	83% vs 80%	Low	Critical

Intervention	Certainty assessment							Effect			Certainty	Importance
	№ of studies	Study design	Risk of bias	Inconsistency	Indirectness	Imprecision	Other considerations	No. of patients	Relative (95% CI)	Absolute (95% CI)		

Recurrence rate

LNG-IUD vs Oral progestin	36	Randomised and nonrandomised studies	Not serious	Serious	Not serious	Not serious	Downgraded due to unexplained heterogeneity across studies, with inconsistent effect estimates and overlapping confidence intervals.	665	1.49 (0.61–2.71)	64% vs 43%	Moderate	Critical
Oral progestin vs Oral + LNG-IUD	35	Randomised and nonrandomised studies	Not serious	Serious	Not serious	Serious	Downgraded due to unexplained heterogeneity across studies, with inconsistent effect estimates and overlapping confidence intervals.	649	3.5833	43% vs 12%	Moderate	Critical

Intervention	Certainty assessment							Effect			Certainty	Importance
	№ of studies	Study design	Risk of bias	Inconsistency	Indirectness	Imprecision	Other considerations	No. of patients	Relative (95% CI)	Absolute (95% CI)		
Oral progestin vs Oral + Hysteroscopy	35	Randomised and nonrandomised studies	Not serious	Serious	Not serious	Not serious	Downgraded due to unexplained heterogeneity across studies, with inconsistent effect estimates and overlapping confidence intervals.	697	2.69 (1.26–6.38)	43% vs 16%	Moderate	Critical
Oral progestin vs LNG-IUD + Hysteroscopy	35	Randomised and nonrandomised studies	Not serious	Serious	Not serious	Not serious	Downgraded due to unexplained heterogeneity across studies, with inconsistent effect estimates and overlapping confidence intervals.	684	3.07 (1.31–8.50)	43% vs 14%	Low	Critical
Oral progestin vs LNG-IUD + GnRHa	33	Randomised and nonrandomised studies	Not serious	Serious	Not serious	Not serious	Downgraded due to unexplained heterogeneity across studies, with inconsistent effect estimates and overlapping confidence intervals.	694	3.58 (1.55–10.20)	43% vs 12%	Moderate	Critical
LNG-IUD vs Oral + LNG-IUD	9	Randomised and nonrandomised studies	Not serious	Not serious	Not serious	Serious	Downgraded due to wide confidence intervals around the pooled effect estimate, limiting precision.	62	5.3333	64% vs 12%	Low	Critical

Intervention	Certainty assessment							Effect			Certainty	Importance
	№ of studies	Study design	Risk of bias	Inconsistency	Indirectness	Imprecision	Other considerations	No. of patients	Relative (95% CI)	Absolute (95% CI)		
LNG-IUD vs Oral + Hysteroscopy	9	Randomised and nonrandomised studies	Not serious	Not serious	Not serious	Not serious	No additional considerations; the pooled effect estimate was consistent across studies with adequate precision.	110	4.00 (1.15–11.50)	64% vs 16%	Low	Critical
LNG-IUD vs LNG-IUD + Hysteroscopy	9	Randomised and nonrandomised studies	Not serious	Not serious	Not serious	Not serious	No additional considerations; the pooled effect estimate was consistent across studies with adequate precision.	97	4.57 (1.19–15.33)	64% vs 14%	Low	Critical
LNG-IUD vs LNG-IUD + GnRHa	9	Randomised and nonrandomised studies	Not serious	Not serious	Not serious	Not serious	No additional considerations; the pooled effect estimate was consistent across studies with adequate precision.	107	5.33 (1.41–18.40)	64% vs 12%	Low	Critical
Oral + Hysteroscopy vs Oral + LNG-IUD	8	Randomised and nonrandomised studies	Not serious	Not serious	Not serious	Serious	Downgraded due to wide confidence intervals around the pooled effect estimate, limiting precision.	94	1.3333	16% vs 12%	Low	Critical
LNG-IUD + Hysteroscopy vs Oral + LNG-IUD	8	Randomised and nonrandomised studies	Not serious	Not serious	Not serious	Serious	Downgraded due to wide confidence intervals around the pooled effect estimate, limiting precision.	81	1.1667	14% vs 12%	Low	Critical

Intervention	Certainty assessment							Effect			Certainty	Importance
	№ of studies	Study design	Risk of bias	Inconsistency	Indirectness	Imprecision	Other considerations	No. of patients	Relative (95% CI)	Absolute (95% CI)		
Oral + LNG-IUD vs LNG-IUD + GnRHa	7	Randomised and nonrandomised studies	Not serious	Not serious	Not serious	Not serious	Downgraded due to minimal differences between intervention effects, with relative effect estimates close to unity, limiting clinical interpretability.	91	1.00 (0.00–6.60)	12% vs 12%	Low	Critical
Oral + Hysteroscopy vs LNG-IUD + Hysteroscopy	7	Randomised and nonrandomised studies	Not serious	Not serious	Not serious	Not serious	Downgraded due to limited number of included studies and participants, reducing the precision and generalisability of the effect estimate.	129	1.14 (0.31–4.50)	16% vs 14%	Low	Critical
Oral + Hysteroscopy vs LNG-IUD + GnRHa	7	Randomised and nonrandomised studies	Not serious	Not serious	Not serious	Not serious	Downgraded due to limited number of included studies and participants, reducing the precision and generalisability of the effect estimate.	139	1.33 (0.36–5.40)	16% vs 12%	Low	Critical
LNG-IUD + Hysteroscopy vs LNG-IUD + GnRHa	7	Randomised and nonrandomised studies	Not serious	Not serious	Not serious	Not serious	Downgraded due to limited number of included studies and participants, reducing the precision and generalisability of the effect estimate.	126	1.17 (0.27–5.20)	14% vs 12%	Low	Critical

Intervention	Certainty assessment							Effect			Certainty	Importance
	№ of studies	Study design	Risk of bias	Inconsistency	Indirectness	Imprecision	Other considerations	No. of patients	Relative (95% CI)	Absolute (95% CI)		
**Pregnancy rate**												
LNG-IUD vs Oral progestin	41	Randomised and nonrandomised studies	Not serious	Serious	Not serious	Not serious	Downgraded due to unexplained heterogeneity across studies, with inconsistent effect estimates and overlapping confidence intervals.	681	1.49 (0.28–2.78)	64% vs 43%	Moderate	Critical
Oral + LNG-IUD vs Oral progestin	41	Randomised and nonrandomised studies	Not serious	Not serious	Not serious	Not serious	No additional considerations; the pooled effect estimate was consistent across studies with adequate precision.	716	1.35 (0.88–2.00)	58% vs 43%	Moderate	Critical
Oral + Hysteroscopy vs Oral progestin	43	Randomised and nonrandomised studies	Not serious	Not serious	Not serious	Not serious	No additional considerations; the pooled effect estimate was consistent across studies with adequate precision.	728	1.33 (0.80–2.03)	57% vs 43%	Moderate	Critical

Intervention	Certainty assessment							Effect			Certainty	Importance
	№ of studies	Study design	Risk of bias	Inconsistency	Indirectness	Imprecision	Other considerations	No. of patients	Relative (95% CI)	Absolute (95% CI)		
LNG-IUD + Hysteroscopy vs Oral progestin	40	Randomised and nonrandomised studies	Not serious	Serious	Not serious	Not serious	Downgraded due to unexplained heterogeneity across studies, with inconsistent effect estimates and overlapping confidence intervals.	682	1.74 (0.66–2.78)	75% vs 43%	Moderate	Critical
Oral progestin vs LNG-IUD + GnRHa	32	Randomised and nonrandomised studies	Not serious	Not serious	Not serious	Not serious	No additional considerations; the pooled effect estimate was consistent across studies with adequate precision.	713	1.39 (0.73–3.33)	43% vs 31%	Low	Critical
LNG-IUD vs Oral + LNG-IUD	10	Randomised and nonrandomised studies	Not serious	Not serious	Not serious	Not serious	No additional considerations; the pooled effect estimate was consistent across studies with adequate precision.	91	1.10 (0.19–2.27)	64% vs 58%	Low	Critical
LNG-IUD vs Oral + Hysteroscopy	12	Randomised and nonrandomised studies	Not serious	Not serious	Not serious	Not serious	No additional considerations; the pooled effect estimate was consistent across studies with adequate precision.	103	1.12 (0.19–2.50)	64% vs 57%	Low	Critical

Intervention	Certainty assessment							Effect			Certainty	Importance
	№ of studies	Study design	Risk of bias	Inconsistency	Indirectness	Imprecision	Other considerations	No. of patients	Relative (95% CI)	Absolute (95% CI)		
LNG-IUD + Hysteroscopy vs LNG-IUD	9	Randomised and nonrandomised studies	Not serious	Serious	Not serious	Not serious	Downgraded due to unexplained heterogeneity across studies, with inconsistent effect estimates and overlapping confidence intervals.	57	1.17 (0.33–7.14)	75% vs 64%	Low	Critical
LNG-IUD vs LNG-IUD + GnRHa	10	Randomised and nonrandomised studies	Not serious	Not serious	Not serious	Not serious	No additional considerations; the pooled effect estimate was consistent across studies with adequate precision.	88	2.06 (0.29–6.67)	64% vs 31%	Low	Critical
Oral + LNG-IUD vs Oral + Hysteroscopy	13	Randomised and nonrandomised studies	Not serious	Not serious	Not serious	Not serious	Downgraded due to minimal differences between intervention effects, with relative effect estimates close to unity, limiting clinical interpretability.	138	1.02 (0.60–1.80)	58% vs 57%	Low	Critical

Live birth rate

Intervention	Certainty assessment							Effect			Certainty	Importance
	№ of studies	Study design	Risk of bias	Inconsistency	Indirectness	Imprecision	Other considerations	No. of patients	Relative (95% CI)	Absolute (95% CI)		
Oral progestin vs LNG-IUD	32	Randomised and nonrandomised studies	Not serious	Not serious	Not serious	Not serious	No additional considerations; the pooled effect estimate was consistent across studies with adequate precision.	299	1.06 (0.37–22.00)	35% vs 33%	Moderate	Critical
Oral + LNG-IUD vs Oral progestin	32	Randomised and nonrandomised studies	Not serious	Not serious	Not serious	Not serious	No additional considerations; the pooled effect estimate was consistent across studies with adequate precision.	326	1.23 (0.64–2.19)	43% vs 35%	Moderate	Critical
Oral + Hysteroscopy vs Oral progestin	35	Randomised and nonrandomised studies	Not serious	Serious	Not serious	Not serious	Downgraded due to unexplained heterogeneity across studies, with inconsistent effect estimates and overlapping confidence intervals.	347	1.26 (0.55–2.41)	44% vs 35%	Moderate	Critical
Oral progestin vs LNG-IUD + GnRHa	32	Randomised and nonrandomised studies	Not serious	Serious	Not serious	Not serious	Downgraded due to unexplained heterogeneity across studies, with inconsistent effect estimates and overlapping confidence intervals.	332	1.75 (0.64–11.00)	35% vs 20%	Moderate	Critical

Intervention	Certainty assessment							Effect			Certainty	Importance
	№ of studies	Study design	Risk of bias	Inconsistency	Indirectness	Imprecision	Other considerations	No. of patients	Relative (95% CI)	Absolute (95% CI)		
Oral + LNG-IUD vs LNG-IUD	8	Randomised and nonrandomised studies	Not serious	Not serious	Not serious	Not serious	Downgraded due to limited number of included studies and participants, reducing the precision and generalisability of the effect estimate.	81	1.30 (0.38–29.50)	43% vs 33%	Low	Critical
Oral + Hysteroscopy vs LNG-IUD	11	Randomised and nonrandomised studies	Not serious	Not serious	Not serious	Not serious	No additional considerations; the pooled effect estimate was consistent across studies with adequate precision.	102	1.33 (0.33–32.50)	44% vs 33%	Low	Critical
LNG-IUD vs LNG-IUD + GnRHa	9	Randomised and nonrandomised studies	Not serious	Not serious	Not serious	Not serious	No additional considerations; the pooled effect estimate was consistent across studies with adequate precision.	87	1.65 (0.05–18.25)	33% vs 20%	Low	Critical
Oral + Hysteroscopy vs Oral + LNG-IUD	12	Randomised and nonrandomised studies	Not serious	Not serious	Not serious	Not serious	Downgraded due to minimal differences between intervention effects, with relative effect estimates close to unity, limiting clinical interpretability.	129	1.02 (0.41–2.32)	44% vs 43%	Low	Critical

Intervention	Certainty assessment							Effect			Certainty	Importance
	№ of studies	Study design	Risk of bias	Inconsistency	Indirectness	Imprecision	Other considerations	No. of patients	Relative (95% CI)	Absolute (95% CI)		
Oral + LNG-IUD vs LNG-IUD + GnRHa	9	Randomised and nonrandomised studies	Not serious	Not serious	Not serious	Not serious	No additional considerations; the pooled effect estimate was consistent across studies with adequate precision.	114	2.15 (0.67–14.75)	43% vs 20%	Low	Critical

Partial response rate

LNG-IUD vs Oral progestin	25	Randomised and nonrandomised studies	Not serious	Not serious	Not serious	Not serious	No additional considerations; the pooled effect estimate was consistent across studies with adequate precision.	416	1.60 (0.18–18.00)	8% vs 5%	Low	Important
Oral + LNG-IUD vs Oral progestin	26	Randomised and nonrandomised studies	Not serious	Serious	Not serious	Not serious	Downgraded due to unexplained heterogeneity across studies, with inconsistent effect estimates and overlapping confidence intervals.	487	2.00 (0.27–18.00)	10% vs 5%	Moderate	Important

Intervention	Certainty assessment							Effect			Certainty	Importance
	№ of studies	Study design	Risk of bias	Inconsistency	Indirectness	Imprecision	Other considerations	No. of patients	Relative (95% CI)	Absolute (95% CI)		
Oral + Hysteroscopy vs Oral progestin	26	Randomised and nonrandomised studies	Not serious	Serious	Not serious	Not serious	Downgraded due to unexplained heterogeneity across studies, with inconsistent effect estimates and overlapping confidence intervals.	476	1.20 (0.00–23.00)	6% vs 5%	Moderate	Important
Oral progestin vs LNG-IUD + Hysteroscopy	22	Randomised and nonrandomised studies	Not serious	Not serious	Not serious	Serious	Downgraded due to low number of events, resulting in wide confidence intervals and reduced certainty of the effect estimate.	398	5	5% vs 1%	Moderate	Important
Oral progestin vs LNG-IUD + GnRHa	22	Randomised and nonrandomised studies	Not serious	Not serious	Not serious	Serious	Downgraded due to low number of events, resulting in wide confidence intervals and reduced certainty of the effect estimate.	399	2.5	5% vs 2%	Low	Important
Oral + LNG-IUD vs LNG-IUD	12	Randomised and nonrandomised studies	Not serious	Not serious	Not serious	Not serious	No additional considerations; the pooled effect estimate was consistent across studies with adequate precision.	217	1.25 (0.17–9.00)	10% vs 8%	Low	Important

Intervention	Certainty assessment							Effect			Certainty	Importance
	№ of studies	Study design	Risk of bias	Inconsistency	Indirectness	Imprecision	Other considerations	No. of patients	Relative (95% CI)	Absolute (95% CI)		
LNG-IUD vs Oral + Hysteroscopy	12	Randomised and nonrandomised studies	Not serious	Not serious	Not serious	Serious	Downgraded due to low number of events, resulting in wide confidence intervals and reduced certainty of the effect estimate.	206	1.3333	8% vs 6%	Low	Important
LNG-IUD vs LNG-IUD + Hysteroscopy	8	Randomised and nonrandomised studies	Not serious	Not serious	Not serious	Serious	Downgraded due to low number of events, resulting in wide confidence intervals and reduced certainty of the effect estimate.	128	8	8% vs 1%	Low	Important
LNG-IUD vs LNG-IUD + GnRHa	9	Randomised and nonrandomised studies	Not serious	Not serious	Not serious	Serious	Downgraded due to low number of events, resulting in wide confidence intervals and reduced certainty of the effect estimate.	129	4	8% vs 2%	Low	Important
Oral + LNG-IUD vs Oral + Hysteroscopy	15	Randomised and nonrandomised studies	Not serious	Not serious	Not serious	Serious	Downgraded due to low number of events, resulting in wide confidence intervals and reduced certainty of the effect estimate.	277	1.6667	10% vs 6%	Low	Important

Intervention	Certainty assessment							Effect			Certainty	Importance
	№ of studies	Study design	Risk of bias	Inconsistency	Indirectness	Imprecision	Other considerations	No. of patients	Relative (95% CI)	Absolute (95% CI)		
Oral + LNG-IUD vs LNG-IUD + Hysteroscopy	11	Randomised and nonrandomised studies	Not serious	Not serious	Not serious	Serious	Downgraded due to low number of events, resulting in wide confidence intervals and reduced certainty of the effect estimate.	199	10	10% vs 1%	Low	Important
Oral + LNG-IUD vs LNG-IUD + GnRHa	10	Randomised and nonrandomised studies	Not serious	Not serious	Not serious	Serious	Downgraded due to low number of events, resulting in wide confidence intervals and reduced certainty of the effect estimate.	200	5	10% vs 2%	Low	Important
Oral + Hysteroscopy vs LNG-IUD + Hysteroscopy	8	Randomised and nonrandomised studies	Not serious	Not serious	Not serious	Serious	Downgraded due to low number of events, resulting in wide confidence intervals and reduced certainty of the effect estimate.	188	6	6% vs 1%	Low	Important
Oral + Hysteroscopy vs LNG-IUD + GnRHa	10	Randomised and nonrandomised studies	Not serious	Not serious	Not serious	Serious	Downgraded due to low number of events, resulting in wide confidence intervals and reduced certainty of the effect estimate.	189	3	6% vs 2%	Low	Important

Intervention	Certainty assessment							Effect			Certainty	Importance
	№ of studies	Study design	Risk of bias	Inconsistency	Indirectness	Imprecision	Other considerations	No. of patients	Relative (95% CI)	Absolute (95% CI)		
LNG-IUD + GnRHa vs LNG-IUD + Hysteroscopy	6	Randomised and nonrandomised studies	Not serious	Not serious	Not serious	Serious	Downgraded due to low number of events, resulting in wide confidence intervals and reduced certainty of the effect estimate.	111	2	2% vs 1%	Low	Important

No response rate

LNG-IUD vs Oral progestin	26	Randomised and nonrandomised studies	Not serious	Not serious	Not serious	Not serious	No additional considerations; the pooled effect estimate was consistent across studies with adequate precision.	441	1.10 (0.43–2.69)	23% vs 21%	Moderate	Important
Oral progestin vs Oral + LNG-IUD	27	Randomised and nonrandomised studies	Not serious	Serious	Not serious	Not serious	Downgraded due to unexplained heterogeneity across studies, with inconsistent effect estimates and overlapping confidence intervals.	512	1.75 (0.46–30.00)	21% vs 12%	Moderate	Important

Intervention	Certainty assessment							Effect			Certainty	Importance
	№ of studies	Study design	Risk of bias	Inconsistency	Indirectness	Imprecision	Other considerations	No. of patients	Relative (95% CI)	Absolute (95% CI)		
Oral progestin vs Oral + Hysteroscopy	27	Randomised and nonrandomised studies	Not serious	Not serious	Not serious	Not serious	No additional considerations; the pooled effect estimate was consistent across studies with adequate precision.	504	1.75 (0.62–7.50)	21% vs 12%	Moderate	Important
Oral progestin vs LNG-IUD + Hysteroscopy	23	Randomised and nonrandomised studies	Not serious	Not serious	Not serious	Not serious	No additional considerations; the pooled effect estimate was consistent across studies with adequate precision.	423	1.75 (0.43–30.00)	21% vs 12%	Low	Important
Oral progestin vs LNG-IUD + GnRHa	23	Randomised and nonrandomised studies	Not serious	Serious	Not serious	Not serious	Downgraded due to unexplained heterogeneity across studies, with inconsistent effect estimates and overlapping confidence intervals.	424	1.00 (0.26–15.00)	21% vs 21%	Low	Important
LNG-IUD vs Oral + LNG-IUD	12	Randomised and nonrandomised studies	Not serious	Not serious	Not serious	Not serious	No additional considerations; the pooled effect estimate was consistent across studies with adequate precision.	217	1.92 (0.46–35.00)	23% vs 12%	Low	Important

Intervention	Certainty assessment							Effect			Certainty	Importance
	№ of studies	Study design	Risk of bias	Inconsistency	Indirectness	Imprecision	Other considerations	No. of patients	Relative (95% CI)	Absolute (95% CI)		
LNG-IUD vs Oral + Hysteroscopy	12	Randomised and nonrandomised studies	Not serious	Not serious	Not serious	Not serious	No additional considerations; the pooled effect estimate was consistent across studies with adequate precision.	209	1.92 (0.62–8.75)	23% vs 12%	Low	Important
LNG-IUD vs LNG-IUD + Hysteroscopy	8	Randomised and nonrandomised studies	Not serious	Not serious	Not serious	Not serious	Downgraded due to limited number of included studies and participants, reducing the precision and generalisability of the effect estimate.	128	1.92 (0.43–35.00)	23% vs 12%	Low	Important
LNG-IUD vs LNG-IUD + GnRHa	9	Randomised and nonrandomised studies	Not serious	Not serious	Not serious	Not serious	No additional considerations; the pooled effect estimate was consistent across studies with adequate precision.	129	1.10 (0.26–17.50)	23% vs 21%	Low	Important
Oral + LNG-IUD vs Oral + Hysteroscopy	15	Randomised and nonrandomised studies	Not serious	Not serious	Not serious	Not serious	Downgraded due to minimal differences between intervention effects, with relative effect estimates close to unity, limiting clinical interpretability.	280	1.00 (0.05–7.00)	12% vs 12%	Low	Important

Intervention	Certainty assessment							Effect			Certainty	Importance
	№ of studies	Study design	Risk of bias	Inconsistency	Indirectness	Imprecision	Other considerations	No. of patients	Relative (95% CI)	Absolute (95% CI)		
Oral + LNG-IUD vs LNG-IUD + Hysteroscopy	11	Randomised and nonrandomised studies	Not serious	Not serious	Not serious	Not serious	Downgraded due to minimal differences between intervention effects, with relative effect estimates close to unity, limiting clinical interpretability.	199	1.00 (0.03–28.00)	12% vs 12%	Low	Important
LNG-IUD + GnRHa vs Oral + LNG-IUD	10	Randomised and nonrandomised studies	Not serious	Serious	Not serious	Not serious	Downgraded due to unexplained heterogeneity across studies, with inconsistent effect estimates and overlapping confidence intervals.	200	1.75 (0.07–50.00)	21% vs 12%	Low	Important
Oral + Hysteroscopy vs LNG-IUD + Hysteroscopy	8	Randomised and nonrandomised studies	Not serious	Not serious	Not serious	Not serious	Downgraded due to minimal differences between intervention effects, with relative effect estimates close to unity, limiting clinical interpretability.	191	1.00 (0.13–21.00)	12% vs 12%	Low	Important
