# Supplementary material for: Oncologic and Reproductive Outcomes of Fertility-Sparing Management in Early-Stage Endometrial Carcinoma: A Systematic Review and Meta-Analysis
Source: Cancers (Basel). 2026 Jan 27;18(3):399. doi: 10.3390/cancers18030399 (PMC12897394; doi:10.3390/cancers18030399)
Supplement: Supplementary file 1 [file cancers-18-00399-s001.zip › Supplemental Table S4.pdf]

Supplementary Table S4. List of excluded studies with reasons for exclusion. This table provides an overview of all articles that were assessed in full text but excluded from the systematic review, along with the specific reasons for exclusion (e.g., wrong intervention, wrong study design, no differentiation between interventions).

Author	Title	Reason for exclusion
**Yang 2019**	Treatment efficiency of comprehensive hysteroscopic evaluation and lesion resection combined with progestin therapy in young women with endometrial atypical hyperplasia and endometrial cancer.	Wrong intervention
**De Marzi 2015**	Hysteroscopic Resection in Fertility-Sparing Surgery for Atypical Hyperplasia and Endometrial Cancer: Safety and Efficacy.	Wrong population
**Chen 2022**	Oncological and reproductive outcomes for gonadotropin-releasing hormone agonist combined with aromatase inhibitors or levonorgestrel-releasing intra-uterine system in women with endometrial cancer or atypical endometrial hyperplasia	Wrong intervention
**Tamauchi 2017**	Efficacy of medroxyprogesterone acetate treatment and retreatment for atypical endometrial hyperplasia and endometrial cancer	Duplication of results
**Atallah 2021**	The use of hysteroscopic endometrectomy in the conservative treatment of early endometrial cancer and atypical hyperplasia in fertile women	Wrong intervention
**Baker 2017**	Nonoperative management of atypical endometrial hyperplasia and grade 1 endometrial cancer with the levonorgestrel intrauterine device in medically ill post-menopausal women	Wrong population
**Fan 2021**	Analysis of pregnancy-associated factors after fertility-sparing therapy in young women with early stage endometrial cancer or atypical endometrial hyperplasia	Wrong study design
**He 2020**	Oncologic and obstetrical outcomes after fertility-preserving retreatment	Wrong population

	in patients with recurrent atypical endometrial hyperplasia and endometrial cancer	
**Donnez 2003**	Conservative treatment may be beneficial for young women with atypical endometrial hyperplasia or endometrial adenocarcinoma	Wrong intervention
**Janda 2021**	Complete pathological response following levonorgestrel intrauterine device in clinically stage 1 endometrial adenocarcinoma: Results of a randomized clinical trial	Wrong study design
**Le Digabel 2006**	Hyperplasies atypiques et carcinomes de l'endomètre de stade I chez la femme jeune désirant une grossesse : le traitement conservateur est-il envisageable ? Résultats d'une étude multicentrique françaiseYoung women with atypical endometrial hyperplasia or endometrial adenocarcinoma stage I: will conservative treatment allow pregnancy? Results of a French multicentric survey	Wrong language
**Marnach 2017**	Oral Progestogens Versus Levonorgestrel-Releasing Intrauterine System for Treatment of Endometrial Intraepithelial Neoplasia	Wrong population
**Masciullo 2021**	Prognostic impact of hysteroscopic resection of endometrial atypical hyperplasia-endometrioid intraepithelial neoplasia and early-stage cancer in combination with megestrol acetate	Wrong population
**Tock 2018**	Fertility Sparing Treatment in Patients With Early Stage Endometrial Cancer, Using a Combination of Surgery and GnRH Agonist: A Monocentric Retrospective Study and Review of the Literature	Wrong intervention
**van Gent MDJM 2016**	Exploring Morphologic and Molecular Aspects of Endometrial Cancer Under Progesterone Treatment in the Context of Fertility Preservation	Wrong study design
**Wang 2022**	[Significance of molecular classification in fertility-sparing treatment of endometrial carcinoma and atypical endometrial hyperplasia]	Wrong language
**Yang 2015**	Prognostic factors of regression and relapse of complex atypical	Wrong study design

	hyperplasia and well-differentiated endometrioid carcinoma with conservative treatment	
**Varma 2007**	The effectiveness of a levonorgestrel-releasing intrauterine system (LNG-IUS) in the treatment of endometrial hyperplasia—A long-term follow-up study	Wrong population
**òrbo 2016**	HE4 is a novel tissue marker for therapy response and progestin resistance in medium- and low-risk endometrial hyperplasia	Wrong study design
**Von Minckwitz 2002**	Adjuvant endocrine treatment with medroxyprogesterone acetate or tamoxifen in stage I and II endometrial cancer—a multicentre, open, controlled, prospectively randomised trial	Wrong population
**Sletten 2019**	Significance of progesterone receptors (PR-A and PR-B) expression as predictors for relapse after successful therapy of endometrial hyperplasia: a retrospective cohort study	Wrong study design
**Wu 2022**	Clinical implications of morular metaplasia in fertility-preserving treatment for atypical endometrial hyperplasia and early endometrial carcinoma patients	Wrong population
**Yoshimura 2022**	Clinical Usefulness of Endometrial Cytology in Determining the Therapeutic Effect of Fertility Preserving Therapy	Wrong study design
**Liu 2022**	Efficacy of Levonorgestrel-intrauterine Releasing System Combined with Goserelin in Treatment of Atypical Endometrial Hyperplasia	Full text not available
**He 2021**	Maintenance Therapy Can Improve the Oncologic Prognosis and Obstetrical Outcome of Patients With Atypical Endometrial Hyperplasia and Endometrial Cancer After Fertility-Preserving Treatment: A Multicenter Retrospective Study	Wrong intervention
**Wang 2021**	Significance of serum and pathological biomarkers in fertility-sparing treatment for endometrial cancer or atypical hyperplasia: a retrospective cohort study	Wrong intervention

**Li 2021**	Insulin Resistance and Metabolic Syndrome Increase the Risk of Relapse For Fertility Preserving Treatment in Atypical Endometrial Hyperplasia and Early Endometrial Cancer Patients	Wrong study design
**Shan 2021**	Effect and Management of Excess Weight in the Context of Fertility-Sparing Treatments in Patients With Atypical Endometrial Hyperplasia and Endometrial Cancer: 8-Year Experience of 227 Cases	Wrong outcomes
**Wang 2021**	Fertility-preserving treatment outcome in endometrial cancer or atypical hyperplasia patients with polycystic ovary syndrome	Wrong population
**Piatek 2021**	The results of different fertility-sparing treatment modalities and obstetric outcomes in patients with early endometrial cancer and atypical endometrial hyperplasia: Case series of 30 patients and systematic review	Wrong population
**Sengal 2021**	Fibroblast growth factor receptor 2 isoforms detected via novel rna ish as predictive biomarkers for progestin therapy in atypical hyperplasia and low-grade endometrial cancer	Wrong outcomes
**Westin 2021**	Prospective phase II trial of levonorgestrel intrauterine device: nonsurgical approach for complex atypical hyperplasia and early-stage endometrial cancer	Wrong population
**Matsuo 2020**	Route-specific association of progestin therapy and concurrent metformin use in obese women with complex atypical hyperplasia	Wrong population
**Kim 2020**	Comparison of diagnostic accuracy between endometrial curettage and aspiration biopsy in patients treated with progestin for endometrial hyperplasia: A Korean gynecologic oncology group study	Wrong outcomes
**Mandelbaum 2020**	Progestin therapy for obese women with complex atypical hyperplasia: levonorgestrel-releasing intrauterine device vs systemic therapy	Wrong population
**Behrouzi 2020**	Baseline serum HE4 but not tissue HE4 expression predicts response to the levonorgestrel-releasing intrauterine system in atypical	Wrong population

	hyperplasia and early stage endometrial cancer	
**Wang 2019**	Impact of treatment duration in fertility-preserving management of endometrial cancer or atypical endometrial hyperplasia	Wrong study design
**Kim 2018**	Fertility-Sparing Management Using Progestin for Young Women with Endometrial Cancer From a Population-Based Study	Wrong population
**Zhou 2017**	Gonadotropin-releasing hormone agonist combined with a levonorgestrel-releasing intrauterine system or letrozole for fertility-preserving treatment of endometrial carcinoma and complex atypical hyperplasia in young women	Wrong study design
**Laurelli 2016**	Long-Term Oncologic and Reproductive Outcomes in Young Women with Early Endometrial Cancer Conservatively Treated: A Prospective Study and Literature Update	Wrong population
**Zhang 2015**	Dual-specificity phosphatase 6 predicts the sensitivity of progestin therapy for atypical endometrial hyperplasia	Wrong outcomes
**Gonthier 2014**	Impact of obesity on the results of fertility-sparing management for atypical hyperplasia and grade 1 endometrial cancer	Wrong intervention
**Gunderson 2014**	Pathologic features associated with resolution of complex atypical hyperplasia and grade 1 endometrial adenocarcinoma after progestin therapy	Wrong study design
**Simpson 2014**	Fertility sparing treatment of complex atypical hyperplasia and low grade endometrial cancer using oral progestin	Wrong population
**Cade 2013**	Long-term outcomes after progestogen treatment for early endometrial cancer	Wrong population
**Goncharenko 2013**	Predictive diagnosis of endometrial hyperplasia and personalized therapeutic strategy in women of fertile age	Wrong outcomes
**Kim 2013**	Comparison of dilatation & curettage and endometrial aspiration biopsy accuracy in patients treated with high-	Wrong population

	dose oral progestin plus levonorgestrel intrauterine system for early-stage endometrial cancer	
**Gallos 2013**	Predictive ability of estrogen receptor (ER), progesterone receptor (PR), COX-2, Mlh1, and Bcl-2 expressions for regression and relapse of endometrial hyperplasia treated with LNG-IUS: A prospective cohort study	Wrong outcomes
**Gallos 2013**	LNG-IUS vs oral progestogen treatment for endometrial hyperplasia: A long-term comparative cohort study	Wrong population
**Bakkum-Gamez 2012**	Conservative management of atypical hyperplasia and grade i endometrial carcinoma: Review of the literature and presentation of a series	Wrong intervention
**Upson 2012**	Biomarkers of progestin therapy resistance and endometrial hyperplasia progression	Wrong outcomes
**Dursun 2012**	A Turkish Gynecologic Oncology Group study of fertility-sparing treatment for early-stage endometrial cancer	Wrong population
**Haoula 2011**	Levonorgestrel intra-uterine system as a treatment option for complex endometrial hyperplasia	Wrong population
**òrbo 2010**	Down-regulated progesterone receptor A and B coinciding with successful treatment of endometrial hyperplasia by the levonorgestrel impregnated intrauterine system	Wrong outcomes
**Cade 2010**	Progestogen treatment options for early endometrial cancer	Wrong population
**Vereide 2005**	Bcl-2, BAX, and apoptosis in endometrial hyperplasia after high dose gestagen therapy: A comparison of responses in patients treated with intrauterine levonorgestrel and systemic medroxyprogesterone	Wrong population
**Montz 2002**	Intrauterine progesterone treatment of early endometrial cancer	Wrong population
**Lago 2022**	Fertility sparing treatment in patients with endometrial cancer (FERT-ENC): a multicentric retrospective study from the Spanish Investigational Network Gynecologic Oncology Group (SPAIN-GOG)	Wrong population

**Chung 2021**	Mismatch repair status influences response to fertility-sparing treatment of endometrial cancer	Wrong population
**Kudesia 2013**	Reproductive and oncologic outcomes after progestin therapy forendometrial complex atypical hyperplasia or carcinoma	Wrong study design
**Greenwald 2016**	Does hormonal therapy for fertility preservation affect the survival ofyoung women with early-stage endometrial cancer?	Wrong population
**Yin 2022**	Clinical outcomes of levonorgestrel-releasing intrauterine device present during controlled ovarian stimulation in patients with early stage endometrioid adenocarcinoma and atypical endometrial hyperplasia after fertility-sparing treatments: 10-year experience in 1 tertiary hospital in China	Wrong study design
**Vereide 2006**	Effect of levonorgestrel IUD and oral medroxyprogesterone acetate on glandular and stromal progesterone receptors (PRA and PRB), and estrogen receptors (ER-alpha and ER-beta) in human endometrial hyperplasia	Wrong population
**Marnach 2017**	Oral Progestogens vs Levonorgestrel-Releasing Intrauterine System for Treatment of Endometrial Intraepithelial Neoplasia<sup/>	Wrong population
**Wu 2023**	Impacts of ovarian reserve on conservative treatment for endometrial cancer and atypical hyperplasia	Wrong study design
**Oishi 2023**	Obstetric outcomes after medroxyprogesterone acetate treatment for early stage endometrial cancer or atypical endometrial hyperplasia: a single hospital-based study	Wrong outcomes
**Barr 2023**	Serum HE4 predicts progestin treatment response in endometrial cancer and atypical hyperplasia: A prognostic study	Wrong outcomes
**Chaudhari 2023**	Comparison of Mirena and Liletta levonorgestrel intrauterine devices for the treatment of endometrial intraepithelial neoplasia and grade 1 endometrioid endometrial cancer	Wrong study design

**Xue 2023**	PTEN mutation predicts unfavorable fertility preserving treatment outcome in the young patients with endometrioid endometrial cancer and atypical hyperplasia	Wrong outcomes
**Xi 2023**	Efficacy and pregnancy outcomes of hysteroscopic surgery combined with progestin as fertility-sparing therapy in patients with early stage endometrial cancer and atypical hyperplasia	Wrong study design
**Wang 2023**	Characteristics of molecular classification in 52 endometrial cancer and atypical hyperplasia patients receiving fertility-sparing treatment	Wrong population
**Ga 2023**	Prognosis of patients with endometrial cancer or atypical endometrial hyperplasia after complete remission with fertility-sparing therapy	Wrong outcomes
**Fu 2023**	Postoperative Adjuvant Treatment in Women with Stage i Endometrial Cancer: A Retrospective Study	Wrong population
**Kudesia 2014**	Reproductive and oncologic outcomes after progestin therapy for endometrial complex atypical hyperplasia or carcinoma	Wrong study design
**Chung 2019**	Oncologic and pregnancy outcomes with fertility-sparing management for early endometrial cancer in young women	Wrong population
**Milishkevich 2022**	The results of fertility-sparing treatment and obstetric outcomes in patients with atypical endometrial hyperplasia and early endometrial cancer: a case series from belarus	Wrong study design
**Lv 2023**	Efficacy of fertility-sparing treatment with LNG-IUS is associated with different ProMisE subtypes of endometrial carcinoma or atypical endometrial hyperplasia	Wrong outcomes
**Lin 2024**	DNA methylation profiling identifies subset of lowgrade endometrial neoplasms with poor response to progestin therapy	Wrong population
**Perri 2011**	Prolonged Conservative Treatment of Endometrial Cancer Patients More Than 1 Pregnancy Can Be Achieved	Wrong study design

Yang B, Xu Y, Zhu Q, Xie L, Shan W, Ning C, Xie B, Shi Y, Luo X, Zhang H, Chen X. Treatment efficiency of comprehensive hysteroscopic evaluation and lesion resection combined with progestin therapy in young women with endometrial atypical hyperplasia and endometrial cancer. Gynecol Oncol. 2019 Apr;153(1):55-62. doi: 10.1016/j.ygyno.2019.01.014. Epub 2019 Jan 21. PMID: 30674421.

De Marzi P, Bergamini A, Luchini S, Petrone M, Taccagni GL, Mangili G, Colombo G, Candiani M. Hysteroscopic Resection in Fertility-Sparing Surgery for Atypical Hyperplasia and Endometrial Cancer: Safety and Efficacy. J Minim Invasive Gynecol. 2015 Nov-Dec;22(7):1178-82. doi: 10.1016/j.jmig.2015.06.004. Epub 2015 Jun 16. PMID: 26092080.

Chen J, Cao D, Yang J, Yu M, Zhou H, Cheng N, Wang J, Zhang Y, Peng P, Shen K. Oncological and reproductive outcomes for gonadotropin-releasing hormone agonist combined with aromatase inhibitors or levonorgestrel-releasing intra-uterine system in women with endometrial cancer or atypical endometrial hyperplasia. Int J Gynecol Cancer. 2022 Dec 5;32(12):1561-1567. doi: 10.1136/ijgc-2022-003882. PMID: 36368706; PMCID: PMC9763228.

Tamauchi S, Kajiyama H, Utsumi F, Suzuki S, Niimi K, Sakata J, Mizuno M, Shibata K, Kikkawa F. Efficacy of medroxyprogesterone acetate treatment and retreatment for atypical endometrial hyperplasia and endometrial cancer. J Obstet Gynaecol Res. 2018 Jan;44(1):151-156. doi: 10.1111/jog.13473. Epub 2017 Nov 9. PMID: 29121428.

Atallah D, El Kassis N, Safi J, El Hachem H, Chahine G, Moubarak M. The use of hysteroscopic endometrectomy in the conservative treatment of early endometrial cancer and atypical hyperplasia in fertile women. Arch Gynecol Obstet. 2021 Nov;304(5):1299-1305. doi: 10.1007/s00404-021-06048-0. Epub 2021 Apr 8. PMID: 33830345.

Baker WD, Pierce SR, Mills AM, Gehrig PA, Duska LR. Nonoperative management of atypical endometrial hyperplasia and grade 1 endometrial cancer with the levonorgestrel intrauterine device in medically ill post-menopausal women. Gynecol Oncol. 2017 Jul;146(1):34-38. doi: 10.1016/j.ygyno.2017.04.006. Epub 2017 Apr 18. PMID: 28427775.

Fan Y, Li X, Wang J, Wang Y, Tian L, Wang J. Analysis of pregnancy-associated factors after fertility-sparing therapy in young women with early stage endometrial cancer or atypical endometrial hyperplasia. Reprod Biol Endocrinol. 2021 Aug 3;19(1):118. doi: 10.1186/s12958-021-00808-y. PMID: 34344384; PMCID: PMC8330111.

He Y, Wang Y, Zhou R, Wang J. Oncologic and obstetrical outcomes after fertility-preserving retreatment in patients with recurrent atypical endometrial hyperplasia and endometrial cancer. Int J Gynecol Cancer. 2020 Dec;30(12):1902-1907. doi: 10.1136/ijgc-2020-001570. Epub 2020 Oct 13. PMID: 33051245.

Jadoul P, Donnez J. Conservative treatment may be beneficial for young women with atypical endometrial hyperplasia or endometrial adenocarcinoma. Fertil Steril. 2003 Dec;80(6):1315-24. doi: 10.1016/s0015-0282(03)01183-x. PMID: 14667859.

Janda M, Robledo KP, Gebski V, Armes JE, Alizart M, Brennan D, Cummings M, Chen C, Leung Y, Sykes P, McNally O, Oehler MK, GraemeWalker, Garrett A, Tang A, Land R, Nicklin JL, Chetty N, Perrin LC, Hoet G, Sowden K, Eva L, Tristram A, Obermair A. Corrigendum to " Complete pathological response following levonorgestrel intrauterine device in clinically stage 1 endometrial adenocarcinoma: Results of a randomized clinical trial" [Gynecologic Oncology 161 (2021) 143-151]. Gynecol Oncol. 2021 Aug;162(2):526. doi: 10.1016/j.ygyno.2021.05.016. Epub 2021 May 28. Erratum for: Gynecol Oncol. 2021 Apr;161(1):143-151. doi: 10.1016/j.ygyno.2021.01.029. PMID: 34053746.

Le Digabel JF, Gariel C, Catala L, Dhainaut C, Madelenat P, Descamps P. Hyperplasies atypiques et carcinomes de l'endomètre de stade I chez la femme jeune désirant une grossesse: le traitement conservateur est-il envisageable? Résultats d'une étude multicentrique française [Young women with atypical endometrial hyperplasia or endometrial adenocarcinoma stage I: will conservative treatment allow pregnancy? Results of a French multicentric survey]. Gynecol Obstet Fertil. 2006 Jan;34(1):27-33. French. doi: 10.1016/j.gyobfe.2005.11.005. Epub 2006 Jan 6. PMID: 16406735.

Marnach ML, Butler KA, Henry MR, Hutz CE, Langstraat CL, Lohse CM, Casey PM. Oral Progestogens Versus Levonorgestrel-Releasing Intrauterine System for Treatment of Endometrial Intraepithelial Neoplasia<sup/>. J Womens Health (Larchmt). 2017 Apr;26(4):368-373. doi: 10.1089/jwh.2016.5774. Epub 2016 Nov 30. PMID: 27901412.

Masciullo V, Trivellizzi N, Zannoni G, Catena U, Moroni R, Fanfani F, Scambia G. Prognostic impact of hysteroscopic resection of endometrial atypical hyperplasia-endometrioid intraepithelial neoplasia and early-stage cancer in combination with megestrol acetate. Am J Obstet Gynecol. 2021 Apr;224(4):408-410. doi: 10.1016/j.ajog.2020.12.1210. Epub 2020 Dec 30. PMID: 33385345.

Tock S, Jadoul P, Squifflet JL, Marbaix E, Baurain JF, Luyckx M. Fertility Sparing Treatment in Patients With Early Stage Endometrial Cancer, Using a Combination of Surgery and GnRH Agonist: A Monocentric Retrospective Study and Review of the Literature. Front Med (Lausanne). 2018 Aug 27;5:240. doi: 10.3389/fmed.2018.00240. PMID: 30211167; PMCID: PMC6119688.

van Gent MD, Nicolae-Cristea AR, de Kroon CD, Osse EM, Kagie MJ, Trimbos JB, Hazelbag HM, Smit VT, Bosse T. Exploring Morphologic and Molecular Aspects of Endometrial Cancer Under Progesterone Treatment in the Context of Fertility Preservation. Int J Gynecol Cancer. 2016 Mar;26(3):483-90. doi: 10.1097/IGC.0000000000000629. PMID: 26825822.

Wang YQ, Kang N, Li LW, Wang ZQ, Zhou R, Shen DH, Wang JL. [Significance of molecular classification in fertility-sparing treatment of endometrial carcinoma and atypical endometrial hyperplasia]. Zhonghua Fu Chan Ke Za Zhi. 2022 Sep 25;57(9):692-700. Chinese. doi: 10.3760/cma.j.cn112141-20220628-00419. PMID: 36177581.

Yang YF, Liao YY, Liu XL, Su SG, Li LZ, Peng NF. Prognostic factors of regression and relapse of complex atypical hyperplasia and well-differentiated endometrioid carcinoma with

conservative treatment. Gynecol Oncol. 2015 Dec;139(3):419-23. doi: 10.1016/j.ygyno.2015.10.015. Epub 2015 Oct 19. PMID: 26494424.

Varma R, Soneja H, Bhatia K, Ganesan R, Rollason T, Clark TJ, Gupta JK. The effectiveness of a levonorgestrel-releasing intrauterine system (LNG-IUS) in the treatment of endometrial hyperplasia--a long-term follow-up study. Eur J Obstet Gynecol Reprod Biol. 2008 Aug;139(2):169-75. doi: 10.1016/j.ejogrb.2008.02.022. Epub 2008 Apr 28. PMID: 18440693.

Ørbo A, Arnes M, Lyså LM, Borgfeldt C, Straume B. HE4 is a novel tissue marker for therapy response and progestin resistance in medium- and low-risk endometrial hyperplasia. Br J Cancer. 2016 Oct 25;115(9):e15. doi: 10.1038/bjc.2016.330. Epub 2016 Oct 6. Erratum for: Br J Cancer. 2016 Sep 6;115(6):725-30. doi: 10.1038/bjc.2016.247. PMID: 27711084; PMCID: PMC5117800.

von Minckwitz G, Loibl S, Brunnert K, Kreienberg R, Melchert F, Mösch R, Neises M, Schermann J, Seufert R, Stiglmayer R, Stosiek U, Kaufmann M. Adjuvant endocrine treatment with medroxyprogesterone acetate or tamoxifen in stage I and II endometrial cancer--a multicentre, open, controlled, prospectively randomised trial. Eur J Cancer. 2002 Nov;38(17):2265-71. doi: 10.1016/s0959-8049(02)00378-7. PMID: 12441263.

Sletten ET, Arnes M, Lyså LM, Larsen M, Ørbo A. Significance of progesterone receptors (PR-A and PR-B) expression as predictors for relapse after successful therapy of endometrial hyperplasia: a retrospective cohort study. BJOG. 2019 Jun;126(7):936-943. doi: 10.1111/1471-0528.15579. Epub 2019 Jan 25. PMID: 30548528.

Wu P, Lv Q, Guan J, Shan W, Chen X, Zhu Q, Luo X. Clinical implications of morular metaplasia in fertility-preserving treatment for atypical endometrial hyperplasia and early endometrial carcinoma patients. Arch Gynecol Obstet. 2022 Oct;306(4):1135-1146. doi: 10.1007/s00404-021-06382-3. Epub 2022 Mar 4. PMID: 35246715; PMCID: PMC9470654.

Yoshimura T, Yamagami W, Takahashi M, Hirano T, Sakai K, Makabe T, Chiyoda T, Banno K, Aoki D. Clinical Usefulness of Endometrial Cytology in Determining the Therapeutic Effect of Fertility Preserving Therapy. Acta Cytol. 2022;66(2):106-113. doi: 10.1159/000520701. Epub 2021 Dec 15. PMID: 34915476; PMCID: PMC8985004.

Jie LIU, Shanshan WANG, Shanshan LI & Xuan LIU. Efficacy of Levonorgestrel-intrauterine Releasing System Combined with Goserelin in Treatment of Atypical Endometrial Hyperplasia. Lat. Am. J. Pharm. 41 (8): 1548-53 (2022)

He Y, Wang J, Wang Y, Zhou R, Lu Q, Liu G, Tang H, Guo H, He M, Wu G. Maintenance Therapy Can Improve the Oncologic Prognosis and Obstetrical Outcome of Patients With Atypical Endometrial Hyperplasia and Endometrial Cancer After Fertility-Preserving Treatment: A Multicenter Retrospective Study. Front Oncol. 2021 Dec 17;11:808881. doi: 10.3389/fonc.2021.808881. PMID: 34976844; PMCID: PMC8718436.

Wang Y, Zhou R, Zhang X, Liu H, Shen D, Wang J. Significance of serum and pathological biomarkers in fertility-sparing treatment for endometrial cancer or atypical hyperplasia: a retrospective cohort study. BMC Womens Health. 2021 Jun 23;21(1):252. doi: 10.1186/s12905-021-01383-5. PMID: 34162378; PMCID: PMC8223344.

Li X, Fan Y, Wang J, Zhou R, Tian L, Wang Y, Wang J. Insulin Resistance and Metabolic Syndrome Increase the Risk of Relapse For Fertility Preserving Treatment in Atypical Endometrial Hyperplasia and Early Endometrial Cancer Patients. Front Oncol. 2021 Nov 30;11:744689. doi: 10.3389/fonc.2021.744689. PMID: 34917501; PMCID: PMC8670892.

Shan Y, Qin M, Yin J, Cai Y, Li Y, Gu Y, Wang W, Wang YX, Chen JY, Jin Y, Pan LY. Effect and Management of Excess Weight in the Context of Fertility-Sparing Treatments in Patients With Atypical Endometrial Hyperplasia and Endometrial Cancer: Eight-Year Experience of 227 Cases. Front Oncol. 2021 Nov 5;11:749881. doi: 10.3389/fonc.2021.749881. PMID: 34804936; PMCID: PMC8602817.

Wang L, Luo X, Wang Q, Lv Q, Wu P, Liu W, Chen X. Fertility-preserving treatment outcome in endometrial cancer or atypical hyperplasia patients with polycystic ovary syndrome. J Gynecol Oncol. 2021 Sep;32(5):e70. doi: 10.3802/jgo.2021.32.e70. Epub 2021 May 25. PMID: 34132069; PMCID: PMC8362812.

Piatek S, Michalski W, Sobiczewski P, Bidzinski M, Szewczyk G. The results of different fertility-sparing treatment modalities and obstetric outcomes in patients with early endometrial cancer and atypical endometrial hyperplasia: Case series of 30 patients and systematic review. Eur J Obstet Gynecol Reprod Biol. 2021 Aug;263:139-147. doi: 10.1016/j.ejogrb.2021.06.007. Epub 2021 Jun 12. PMID: 34214800.

Sengal AT, Smith D, Rogers R, Snell CE, Williams ED, Pollock PM. Fibroblast Growth Factor Receptor 2 Isoforms Detected via Novel RNA ISH as Predictive Biomarkers for Progestin Therapy in Atypical Hyperplasia and Low-Grade Endometrial Cancer. Cancers (Basel). 2021 Apr 3;13(7):1703. doi: 10.3390/cancers13071703. PMID: 33916719; PMCID: PMC8038411.

Westin SN, Fellman B, Sun CC, Broaddus RR, Woodall ML, Pal N, Urbauer DL, Ramondetta LM, Schmeler KM, Soliman PT, Fleming ND, Burzawa JK, Nick AM, Milbourne AM, Yuan Y, Lu KH, Bodurka DC, Coleman RL, Yates MS. Prospective phase II trial of levonorgestrel intrauterine device: nonsurgical approach for complex atypical hyperplasia and early-stage endometrial cancer. Am J Obstet Gynecol. 2021 Feb;224(2):191.e1-191.e15. doi: 10.1016/j.ajog.2020.08.032. Epub 2020 Aug 15. PMID: 32805208; PMCID: PMC7855308.

Matsuo K, Mandelbaum RS, Ciccone M, Khoshchehreh M, Pursuwani H, Morocco EB, Matsuzaki S, Dancz CE, Ozel B, Paulson RJ, Roman L. Route-specific association of progestin therapy and concurrent metformin use in obese women with complex atypical hyperplasia. Int J Gynecol Cancer. 2020 Sep;30(9):1331-1339. doi: 10.1136/ijgc-2020-001362. Epub 2020 May 5. Erratum in: Int J Gynecol Cancer. 2020 Dec;30(12):2022. doi: 10.1136/ijgc-2020-001362corr1. PMID: 32376736; PMCID: PMC7521080.

Kim MK, Seong SJ. Response to comment on: Comparison of diagnostic accuracy between endometrial curettage and aspiration biopsy in patients treated with progestin for endometrial hyperplasia: a Korean Gynecologic Oncology Group study. J Gynecol Oncol. 2020 Sep;31(5):e89. doi: 10.3802/jgo.2020.31.e89. PMID: 32808507; PMCID: PMC7440991.

Mandelbaum RS, Ciccone MA, Nusbaum DJ, Khoshchehreh M, Purswani H, Morocco EB, Smith MB, Matsuzaki S, Dancz CE, Ozel B, Roman LD, Paulson RJ, Matsuo K. Progestin therapy for obese women with complex atypical hyperplasia: levonorgestrel-releasing intrauterine device vs systemic therapy. Am J Obstet Gynecol. 2020 Jul;223(1):103.e1-103.e13. doi: 10.1016/j.ajog.2019.12.273. Epub 2020 Jan 21. PMID: 31978437; PMCID: PMC7751571.

Behrouzi R, Ryan NAJ, Barr CE, Derbyshire AE, Wan YL, Maskell Z, Stocking K, Pemberton PW, Bolton J, McVey RJ, Crosbie EJ. Baseline Serum HE4 But Not Tissue HE4 Expression Predicts Response to the Levonorgestrel-Releasing Intrauterine System in Atypical Hyperplasia and Early Stage Endometrial Cancer. Cancers (Basel). 2020 Jan 23;12(2):276. doi: 10.3390/cancers12020276. PMID: 31979212; PMCID: PMC7073190.

Wang Y, Zhou R, Wang H, Liu H, Wang J. Impact of treatment duration in fertility-preserving management of endometrial cancer or atypical endometrial hyperplasia. Int J Gynecol Cancer. 2019 May;29(4):699-704. doi: 10.1136/ijgc-2018-000081. Epub 2019 Mar 1. PMID: 30826750.

Kim SR, van der Zanden C, Ikiz H, Kuzelijevic B, Havelock J, Kwon JS. Fertility-Sparing Management Using Progestin for Young Women with Endometrial Cancer From a Population-Based Study. J Obstet Gynaecol Can. 2018 Mar;40(3):328-333. doi: 10.1016/j.jogc.2017.06.037. Epub 2017 Oct 3. PMID: 28986185.

Zhou H, Cao D, Yang J, Shen K, Lang J. Gonadotropin-Releasing Hormone Agonist Combined With a Levonorgestrel-Releasing Intrauterine System or Letrozole for Fertility-Preserving Treatment of Endometrial Carcinoma and Complex Atypical Hyperplasia in Young Women. Int J Gynecol Cancer. 2017 Jul;27(6):1178-1182. doi: 10.1097/IGC.0000000000001008. PMID: 28562472.

Laurelli G, Falcone F, Gallo MS, Scala F, Losito S, Granata V, Cascella M, Greggi S. Long-Term Oncologic and Reproductive Outcomes in Young Women With Early Endometrial Cancer Conservatively Treated: A Prospective Study and Literature Update. Int J Gynecol Cancer. 2016 Nov;26(9):1650-1657. doi: 10.1097/IGC.0000000000000825. PMID: 27654262.

Zhang H, Yan L, Bai Y, Li C, Guo Q, Wang C, Zhao X, Li M. Dual-specificity phosphatase 6 predicts the sensitivity of progestin therapy for atypical endometrial hyperplasia. Gynecol Oncol. 2015 Mar;136(3):549-53. doi: 10.1016/j.ygyno.2014.11.008. Epub 2014 Nov 14. PMID: 25451692.

Gonthier C, Walker F, Luton D, Yazbeck C, Madelenat P, Koskas M. Impact of obesity on the results of fertility-sparing management for atypical hyperplasia and grade 1 endometrial

cancer. Gynecol Oncol. 2014 Apr;133(1):33-7. doi: 10.1016/j.ygyno.2013.11.007. PMID: 24680589.

Gunderson CC, Dutta S, Fader AN, Maniar KP, Nasseri-Nik N, Bristow RE, Diaz-Montes TP, Palermo R, Kurman RJ. Pathologic features associated with resolution of complex atypical hyperplasia and grade 1 endometrial adenocarcinoma after progestin therapy. Gynecol Oncol. 2014 Jan;132(1):33-7. doi: 10.1016/j.ygyno.2013.11.033. Epub 2013 Dec 4. PMID: 24316307.

Simpson AN, Feigenberg T, Clarke BA, Gien LT, Ismiil N, Laframboise S, Massey C, Ferguson SE. Fertility sparing treatment of complex atypical hyperplasia and low grade endometrial cancer using oral progestin. Gynecol Oncol. 2014 May;133(2):229-33. doi: 10.1016/j.ygyno.2014.02.020. Epub 2014 Feb 19. PMID: 24561246.

Cade TJ, Quinn MA, Rome RM, Neesham D. Long-term outcomes after progestogen treatment for early endometrial cancer. Aust N Z J Obstet Gynaecol. 2013 Dec;53(6):566-70. doi: 10.1111/ajo.12142. Epub 2013 Oct 19. PMID: 24138444.

Goncharenko VM, Beniuk VA, Kalenska OV, Demchenko OM, Spivak MY, Bubnov RV. Predictive diagnosis of endometrial hyperplasia and personalized therapeutic strategy in women of fertile age. EPMA J. 2013 Dec 6;4(1):24. doi: 10.1186/1878-5085-4-24. PMID: 24314145; PMCID: PMC3866390.

Kim MK, Seong SJ, Song T, Kim ML, Yoon BS, Jun HS, Lee GH, Lee YH. Comparison of dilatation & curettage and endometrial aspiration biopsy accuracy in patients treated with high-dose oral progestin plus levonorgestrel intrauterine system for early-stage endometrial cancer. Gynecol Oncol. 2013 Sep;130(3):470-3. doi: 10.1016/j.ygyno.2013.06.035. Epub 2013 Jun 30. PMID: 23822890.

Gallos ID, Devey J, Ganesan R, Gupta JK. Predictive ability of estrogen receptor (ER), progesterone receptor (PR), COX-2, Mlh1, and Bcl-2 expressions for regression and relapse of endometrial hyperplasia treated with LNG-IUS: a prospective cohort study. Gynecol Oncol. 2013 Jul;130(1):58-63. doi: 10.1016/j.ygyno.2013.04.016. Epub 2013 Apr 17. PMID: 23603367.

Jamie N. Bakkum-Gamez, Eleftheria Kalogera, Gary L. Keeney, Andrea Mariani, Karl C. Podratz, Sean C. Dowdy. Conservative Management of Atypical Hyperplasia and Grade I Endometrial Carcinoma: Review of the Literature and Presentation of a Series. Journal of Gynecologic Surgery. 14 August 2012. (28)4 https://doi.org/10.1089/gyn.2012.0011

Upson K, Allison KH, Reed SD, Jordan CD, Newton KM, Swisher EM, Doherty JA, Garcia RL. Biomarkers of progestin therapy resistance and endometrial hyperplasia progression. Am J Obstet Gynecol. 2012 Jul;207(1):36.e1-8. doi: 10.1016/j.ajog.2012.05.012. Epub 2012 May 16. PMID: 22727345; PMCID: PMC3398620.

Haoula ZJ, Walker KF, Powell MC. Levonorgestrel intra-uterine system as a treatment option for complex endometrial hyperplasia. Eur J Obstet Gynecol Reprod Biol. 2011 Nov;159(1):176-9. doi: 10.1016/j.ejogrb.2011.06.025. Epub 2011 Jul 7. PMID: 21741152.

Orbo A, Arnes M, Pettersen I, Larsen K, Hanssen K, Moe B. Down-regulated progesterone receptor A and B coinciding with successful treatment of endometrial hyperplasia by the levonorgestrel impregnated intrauterine system. Acta Obstet Gynecol Scand. 2010 Nov;89(11):1438-46. doi: 10.3109/00016349.2010.512068. PMID: 20955098.

Cade TJ, Quinn MA, Rome RM, Neesham D. Progestogen treatment options for early endometrial cancer. BJOG. 2010 Jun;117(7):879-84. doi: 10.1111/j.1471-0528.2010.02552.x. Epub 2010 Apr 12. PMID: 20394609.

Vereide AB, Kaino T, Sager G, Ørbo A; Scottish Gynaecological Clinical Trials Group. Bcl-2, BAX, and apoptosis in endometrial hyperplasia after high dose gestagen therapy: a comparison of responses in patients treated with intrauterine levonorgestrel and systemic medroxyprogesterone. Gynecol Oncol. 2005 Jun;97(3):740-50. doi: 10.1016/j.ygyno.2005.02.030. PMID: 15885761.

Montz FJ, Bristow RE, Bovicelli A, Tomacruz R, Kurman RJ. Intrauterine progesterone treatment of early endometrial cancer. Am J Obstet Gynecol. 2002 Apr;186(4):651-7. doi: 10.1067/mob.2002.122130. PMID: 11967486.

Lago V, Marina T, Laseca Modrego M, Gil-Ibañez B, Rodriguez JR, Domingo J, Minig L, Padilla-Iserte P, Arencibia Sánchez O, Sala Ferichola M, Munmann M, Martín Salamanca B, Iacoponi S, Cabrera S, Coronado P, Utrilla-Layna J, Bataller Á, Fiol G, Corbalán S, Espinosa E, Gil-Moreno A, Domingo S; Spanish Society of Gynecology and Obstetrics Spanish Investigational Network Gynecologic Oncology Group (SPAIN-GOG). Fertility sparing treatment in patients with endometrial cancer (FERT-ENC): a multicentric retrospective study from the Spanish Investigational Network Gynecologic Oncology Group (SPAIN-GOG). Arch Gynecol Obstet. 2022 Sep;306(3):821-828. doi: 10.1007/s00404-021-06375-2. Epub 2022 Feb 4. PMID:35122158.

Chung YS, Woo HY, Lee JY, Park E, Nam EJ, Kim S, Kim SW, Kim YT. Mismatch repair status influences response to fertility-sparing treatment of endometrial cancer. Am J Obstet Gynecol. 2021 Apr;224(4):370.e1-370.e13. doi: 10.1016/j.ajog.2020.10.003. Epub 2020 Oct 9. PMID: 33039397.

Kudesia R, Singer T, Caputo TA, Holcomb KM, Kligman I, Rosenwaks Z, Gupta D. Reproductive and oncologic outcomes after progestin therapy for endometrial complex atypical hyperplasia or carcinoma. Am J Obstet Gynecol. 2014 Mar;210(3):255.e1-4. doi: 10.1016/j.ajog.2013.11.001. Epub 2013 Nov 8. PMID: 24211482.

Greenwald ZR, Huang LN, Wissing MD, Franco EL, Gotlieb WH. Does hormonal therapy for fertility preservation affect the survival of young women with early-stage endometrial

cancer? Cancer. 2017 May 1;123(9):1545-1554. doi: 10.1002/cncr.30529. Epub 2016 Dec 27. PMID: 28026855.

Yin J, Li Y, Wang H, Wang W, Gu Y, Jin Y, Deng C, Pan L. Clinical outcomes of levonorgestrel-releasing intrauterine device present during controlled ovarian stimulation in patients with early stage endometrioid adenocarcinoma and atypical endometrial hyperplasia after fertility-sparing treatments: 10-year experience in one tertiary hospital in China. Eur J Obstet Gynecol Reprod Biol. 2023 Jan;280:83-88. doi: 10.1016/j.ejogrb.2022.11.009. Epub 2022 Nov 11. PMID: 36436458.

Vereide AB, Kaino T, Sager G, Arnes M, Ørbo A. Effect of levonorgestrel IUD and oral medroxyprogesterone acetate on glandular and stromal progesterone receptors (PRA and PRB), and estrogen receptors (ER-alpha and ER-beta) in human endometrial hyperplasia. Gynecol Oncol. 2006 May;101(2):214-23. doi: 10.1016/j.ygyno.2005.10.030. Epub 2005 Dec 1. PMID: 16325240.

74. Marnach ML, Butler KA, Henry MR, Hutz CE, Langstraat CL, Lohse CM, Casey PM. Oral Progestogens Versus Levonorgestrel-Releasing Intrauterine System for Treatment of Endometrial Intraepithelial Neoplasia<sup/>;. J Womens Health (Larchmt). 2017 Apr;26(4):368-373. doi: 10.1089/jwh.2016.5774. Epub 2016 Nov 30. PMID: 27901412.

Wu P, Shan W, Xue Y, Wang L, Liu S, Chen X, Luo X. Impacts of ovarian reserve on conservative treatment for endometrial cancer and atypical hyperplasia. Front Endocrinol (Lausanne). 2024 Jan 5;14:1286724. doi: 10.3389/fendo.2023.1286724. PMID: 38250737;

Barr CE, Sergeant JC, Agnew HJ, Bolton J, McVey RJ, Crosbie EJ. Serum HE4 predicts progestin treatment response in endometrial cancer and atypical hyperplasia: A prognostic study. BJOG. 2023 Jul;130(8):941-948. doi: 10.1111/1471-0528.17417. Epub 2023 Feb 15. PMID: 36715558.

Chaudhari SR, Lai TS, Zakhour M, Myung Shin S, Baltayan A, Tan H, Cohen JG. Comparison of Mirena and Liletta levonorgestrel intrauterine devices for the treatment of endometrial intraepithelial neoplasia and grade 1 endometrioid endometrial cancer. Gynecol Oncol Rep. 2023 Aug 19;49:101257. doi: 10.1016/j.gore.2023.101257. PMID: 37691755; PMCID: PMC10485590.

Xue Y, Dong Y, Lou Y, Lv Q, Shan W, Wang C, Chen X. PTEN mutation predicts unfavorable fertility preserving treatment outcome in the young patients with endometrioid endometrial cancer and atypical hyperplasia. J Gynecol Oncol. 2023 Jul;34(4):e53. doi: 10.3802/jgo.2023.34.e53. Epub 2023 Mar 10. PMID: 36929579; PMCID: PMC10323299.

Xi Y, Liu G, Liu D, Jiang J, Gong R. Efficacy and pregnancy outcomes of hysteroscopic surgery combined with progestin as fertility-sparing therapy in patients with early stage endometrial cancer and atypical hyperplasia. Arch Gynecol Obstet. 2023 Feb;307(2):583-590. doi: 10.1007/s00404-022-06626-w. Epub 2022 Jun 8. PMID: 35674831.

Wang Y, Bo L, Fan X, Kang N, Zhang X, Tian L, Zhou R, Wang J. Molecular Classification Guides Fertility-Sparing Treatment for Endometrial Cancer and Atypical Hyperplasia Patients. Curr Oncol. 2025 May 30;32(6):317. doi: 10.3390/curroncol32060317. PMID: 40558260; PMCID: PMC12192213.

Ga H, Taguchi A, Honjoh H, Nishijima A, Eguchi S, Miyamoto Y, Sone K, Mori M, Osuga Y. Prognosis of patients with endometrial cancer or atypical endometrial hyperplasia after complete remission with fertility-sparing therapy. Arch Gynecol Obstet. 2023 Nov;308(5):1629-1634. doi: 10.1007/s00404-023-07077-7. Epub 2023 Jun 13. PMID: 37310452; PMCID: PMC10520125.

Fu P, Sun H, Zhou T, Cui P, Wang S, Liu R. Postoperative Adjuvant Treatment in Women with Stage I Endometrial Cancer: A Retrospective Study. Int J Clin Pract. 2023 Mar 31;2023:4007616. doi: 10.1155/2023/4007616. PMID: 37035519; PMCID: PMC10081899.

Kudesia R, Singer T, Caputo TA, Holcomb KM, Kligman I, Rosenwaks Z, Gupta D. Reproductive and oncologic outcomes after progestin therapy for endometrial complex atypical hyperplasia or carcinoma. Am J Obstet Gynecol. 2014 Mar;210(3):255.e1-4. doi: 10.1016/j.ajog.2013.11.001. Epub 2013 Nov 8. PMID: 24211482.

Y.S. Chung, J.Y. Lee, E.J. Nam, S. Kim, S.W. Kim, Y.T. Kim. EP498 Oncologic and pregnancy outcomes with fertility-sparing management for early endometrial cancer in young women. November 2019. Volume 29, Supplement 4, A311

Alena G Milishkevich, Siarhei A Mavrichev, Olga P Matylevich, Alena V Dalamanava, Sviatlana Y Shelkovich, 2022-RA-427-ESGO The results of fertility-sparing treatment and obstetric outcomes in patients with atypical endometrial hyperplasia andearly endometrial cancer: a case series from belarus, International Journal of Gynecological Cancer, Volume 32, Supplement 2, 2022, Page A170, ISSN 1048-891X, https://doi.org/10.1136/ijgc-2022-ESGO.365.

Lv X, Guo L, Wang C. Efficacy of fertility-sparing treatment with LNG-IUS is associated with different ProMisE subtypes of endometrial carcinoma or atypical endometrial hyperplasia. J Gynecol Oncol. 2024 May;35(3):e27. doi: 10.3802/jgo.2024.35.e27. Epub 2023 Dec 18. PMID: 38216133; PMCID: PMC11107283.

Lawrence H. Lin, Kyriaki Founta, Nyasha Chambwe, Deborah F. DeLair; Abstract B030: DNA methylation profiling identifies subset of low-grade endometrial neoplasms with poor response to progestin therapy. Clin Cancer Res 1 March 2024; 30 (5_Supplement): B030. https://doi.org/10.1158/1557-3265.ENDO24-B030

Perri T, Korach J, Gotlieb WH, Beiner M, Meirow D, Friedman E, Ferenczy A, Ben-Baruch G. Prolonged conservative treatment of endometrial cancer patients: more than 1 pregnancy can be achieved. Int J Gynecol Cancer. 2011 Jan;21(1):72-8. doi: 10.1097/IGC.0b013e31820003de. PMID: 21178572.
