# Supplementary figures and images for: Oncologic and Reproductive Outcomes of Fertility-Sparing Management in Early-Stage Endometrial Carcinoma: A Systematic Review and Meta-Analysis
Source: Cancers (Basel). 2026 Jan 27;18(3):399. doi: 10.3390/cancers18030399 (PMC12897394; doi:10.3390/cancers18030399)

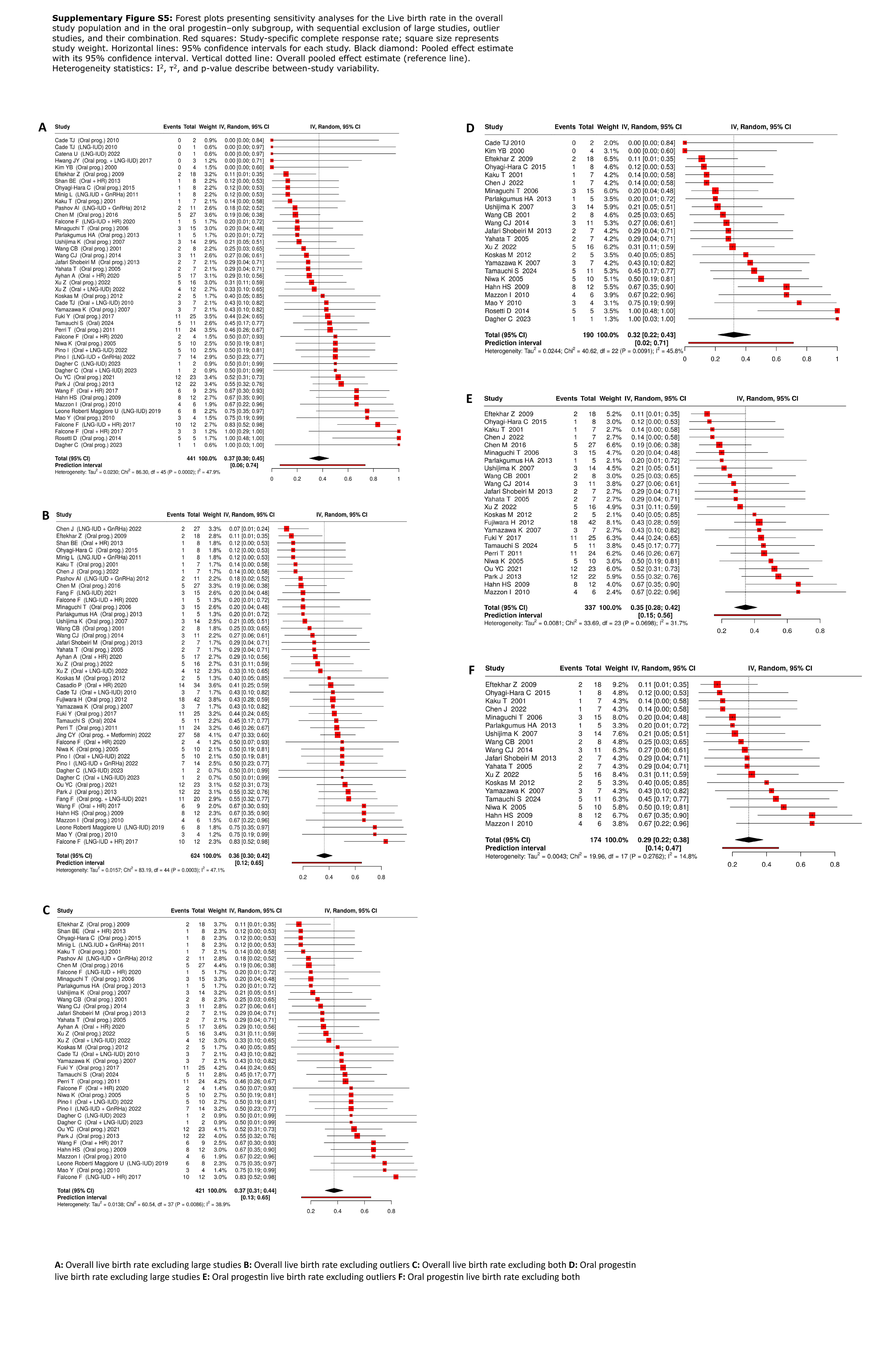

Supplement: Supplementary file 1 [file cancers-18-00399-s001.zip › Supplementary Figure S5.png]

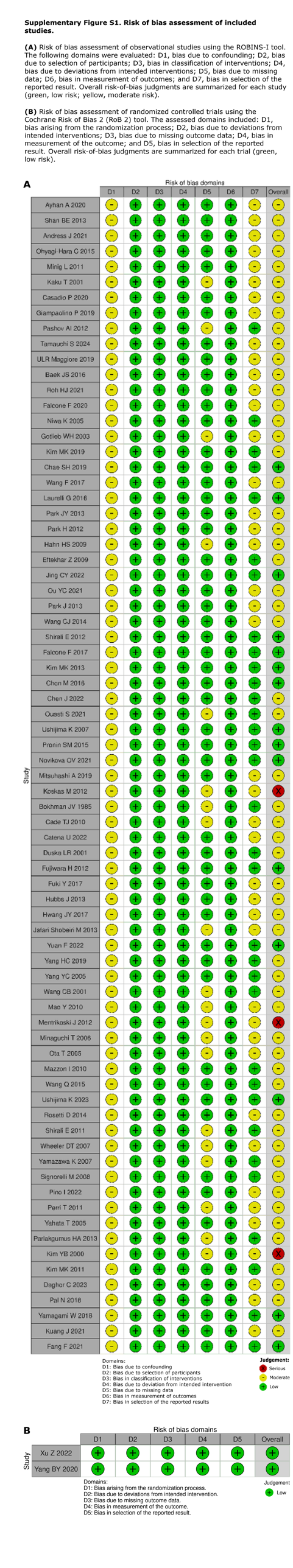

Supplement: Supplementary file 1 [file cancers-18-00399-s001.zip › Supplementary Figure S1.png]

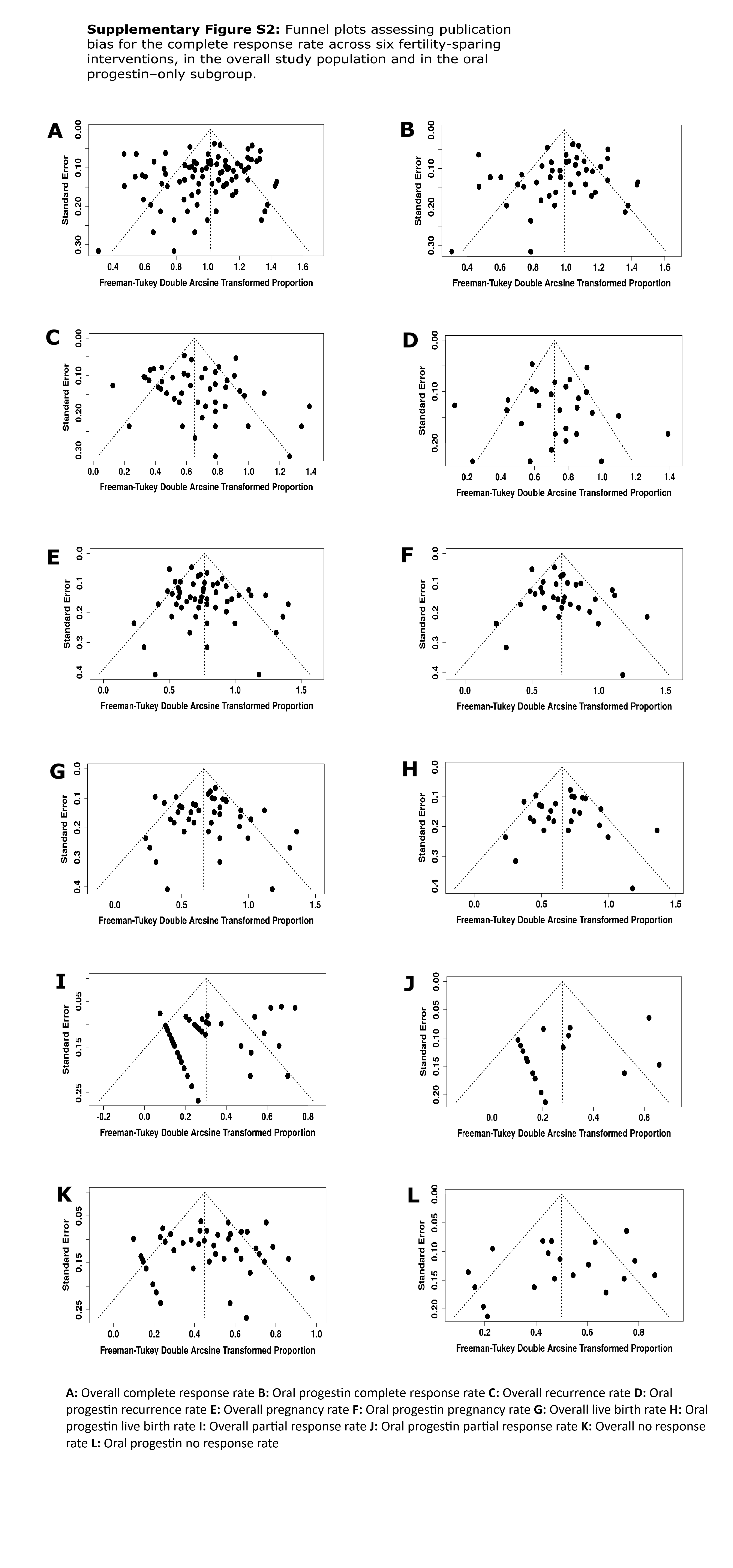

Supplement: Supplementary file 1 [file cancers-18-00399-s001.zip › Supplementary Figure S2.png]

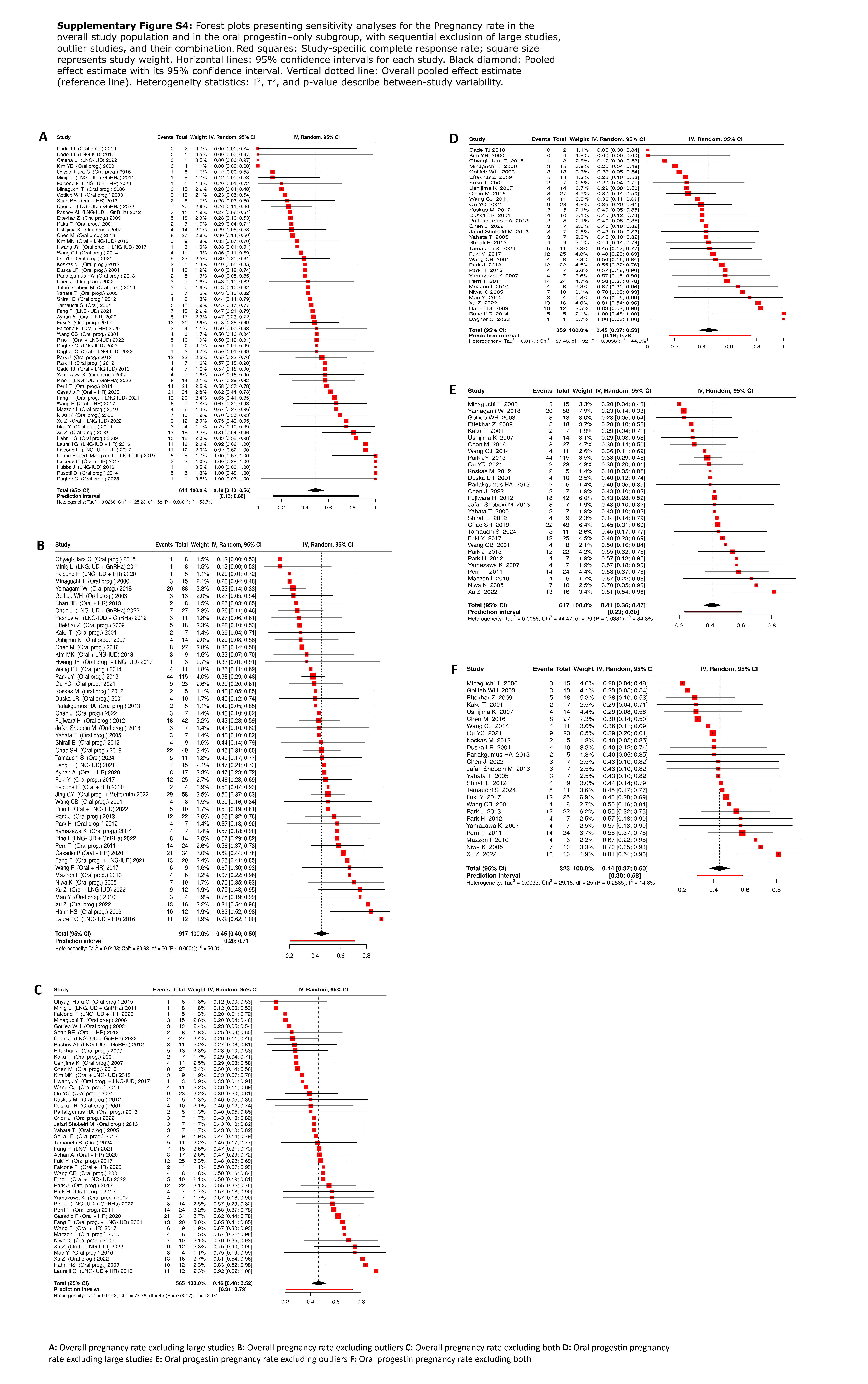

Supplement: Supplementary file 1 [file cancers-18-00399-s001.zip › Supplementary Figure S4.png]

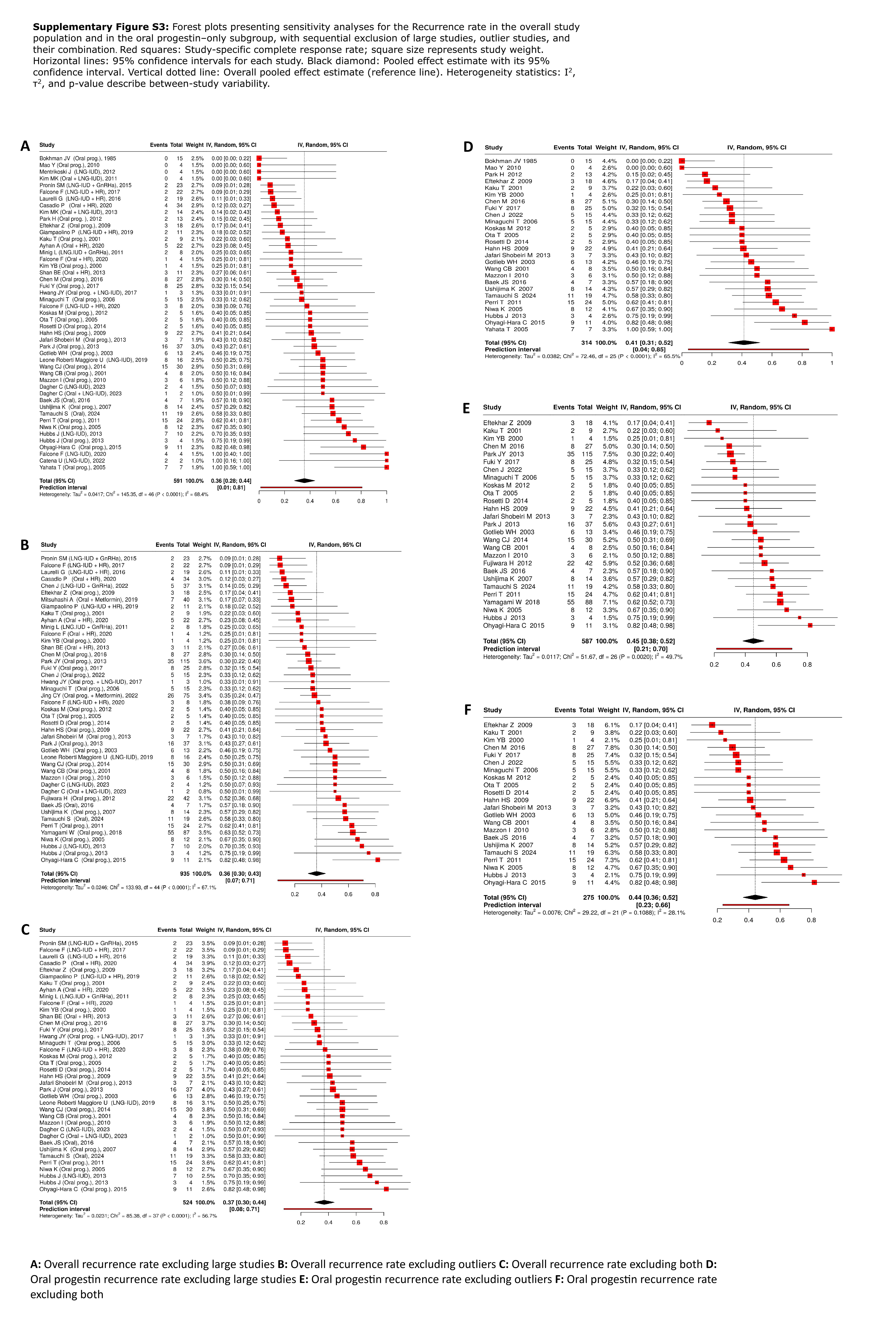

Supplement: Supplementary file 1 [file cancers-18-00399-s001.zip › Supplementary Figure S3.png]
